# Development of a Stereoselective Synthesis of Isomers of (+)-Disorazole Z1’s Lateral Chain

**DOI:** 10.1021/acs.joc.5c01789

**Published:** 2025-10-29

**Authors:** Thomas J. Bauer, Phil Köhler, Oliver Spieß, Dieter Schinzer

**Affiliations:** † Institute for Chemistry, 9376 Otto-von-Guericke-University Magdeburg, Universitätsplatz 2, 39106 Magdeburg, Germany; ‡ Institute of Inorganic and Analytical Chemistry, 9378 Friedrich Schiller University Jena, Humboldtstraße 8, 07743 Jena, Germany

## Abstract

(+)-Disorazole Z1 (**1**) bears two lateral chains, each including a chiral quaternary carbon center with an ester moiety and a methyl group surrounded by two chiral secondary alcohols. Herein, we present different routes to synthesize the lateral chain, unveiling details of their development. The strategies, which include two consecutive aldol steps with a lactone intermediate, led to natural **2** and non-natural **3** lateral chain precursors with very good stereoselectivity. The stereochemistry was confirmed by X-ray crystallography. The results show an alternative lactonization procedure as well as the impact of detailed fine adjustments of the transesterification of a δ-lactone ring on retaining a sensitive triethylsilyl protecting group and avoiding relactonization. Furthermore, the influence of polysubstituted lactones on the stereoselectivity of the subsequent aldol reaction is shown. Vicinal hydroxy groups around the chiral quaternary center were protected in a sequence that allows orthogonal deprotection if a lateral chain should be incorporated in a more complicated structure. This work was crucial for our total synthesis of (+)-disorazole Z1 and could also be helpful for syntheses of other chiral acyclic α-quaternary-β,β′-hydroxy esters.

## Graphical abstract

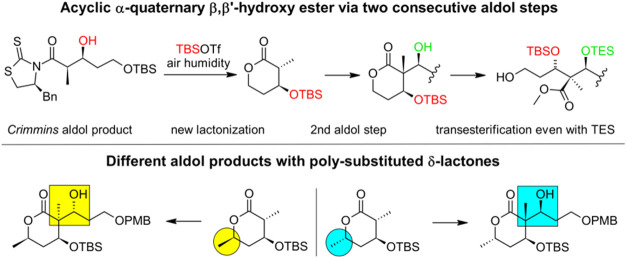

## Introduction

Chiral quaternary carbon centers are ubiquitous in natural products and drugs derived thereof.^1,2^ A special type of chiral quaternary carbon centers are those bearing four different carbon substituents.^3^ Syntheses of these centers could be of great interest for the development of new drugs or other organic compounds. However, it is problematic because of steric repulsion between the carbon substituents and structural instability.^4^ Although in recent years a number of chemical procedures have been developed to overcome problems of synthesis, the construction of vicinal stereocenters and the enantioselective synthesis of chiral all-carbon quaternary centers in acyclic systems are still important topics of current research.^5,6^

A well-known example of an acyclic system containing a chiral all-carbon quaternary center together with vicinal stereocenters is the lateral chain of (+)-disorazole Z1 (**1**).^7^ Although this compound, which is naturally produced by the myxobacterium *Sorangium cellulosum* strain So ce 1875, is of outstanding interest for pharmaceutical applications because of its highly potent cytotoxic activity, its total synthesis took a long time, despite various studies addressing this challenge.^8–10^ The major reason for this delay was the difficulty to synthesize the lateral chain’s chiral all-carbon quaternary center together with two vicinal chiral centers bearing a hydroxy group, respectively. Consequently, very recently, the first total synthesis of (+)-disorazole Z1 (**1**) has been disclosed from our lab.^11^ During that endeavor, the correct stereochemistry of synthetic **1** was proven by comparing spectroscopic data (^1^H NMR and ^13^C­{^1^H}-NMR) and the specific rotation value of synthetic **1** with natural (+)-disorazole Z1.

In this paper, we describe key reactions leading to our successful strategy and show how changes of reaction conditions influence the stereoselectivity.^11^ Although recent crystallographic studies suggested that all three stereocenters of each lateral chain of **1** have an (*S*)-configuration as shown in Figure 1, we focused on a principle approach of synthesizing a chiral all-carbon quaternary center via diastereoselective aldol reactions.^7,12^ We analyzed the product of each aldol reaction in detail using X-ray diffraction and NMR techniques in parallel for proofing the stereoselectivity of all aldol addition steps performed. Hence, we gained information which enforces the NMR proof,^11^ demonstrates the advantage of lactone intermediates in the case of two consecutive aldol steps, and extends the knowledge of how substituents of a lactone induce the diastereoselectivity of an aldol addition. This work may provide more information about the synthesis of other compounds with chiral triads containing quaternary carbon centers.

**Figure 1:**
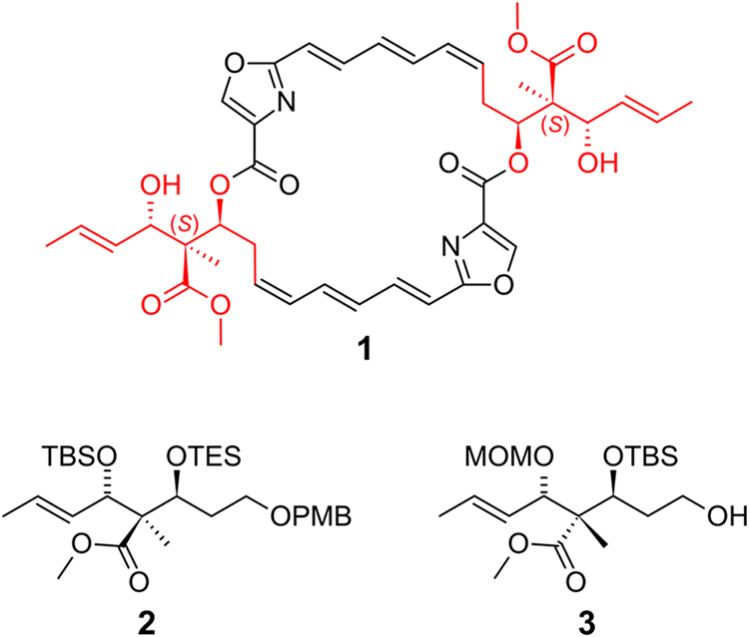
(+)-Disorazole Z1 (**1**) with red-marked lateral chains; synthesized natural **2** and non-natural **3** lateral chain precursors.

## Results and Discussion

Recently, we described the synthesis of the lateral chain of (+)-disorazole Z1 via two consecutive aldol steps.^11^ We started our strategy according to Crimmins et al. and prepared *N*-propionyl-thiazolidinethione **4** by the use of readily available (*S*)-phenylalanine.^13^ This compound was used also in this study as the starting material for all first aldol steps described in scheme 1–3. The chiral quaternary carbon center was created by a second aldol reaction with a lactone because the stereochemical outcome of this reaction could be better controlled by the rigid ring system. Brückner’s findings on aldol additions with boron enolates of δ-lactones were applied in this study.^14^

As a first approach, we combined compound **4** with a *tert*-butyldimethylsilyl (TBS)-protected propanal **5** to get a non-Evans *syn* aldol product **6**.^11^ Before running the second aldol step, we protected the secondary alcohol of **6** as a TBS ether prior to cleaving the primary TBS ether needed for the lactone formation. Accidentally added TBS triflate without a base at −78 °C in dichloromethane yielded the TBS-protected δ-lactone **7** during warm-up to room temperature within 2 h (Scheme 1).

This surprising phenomenon was studied by small-scale experiments, which showed that the air humidity triggered the reaction. It is repeatable but variable in terms of the yield. An open flask during the thawing phase is necessary to provide air humidity. We believe that TBS triflate is attacked by the β-hydroxy group of **6** generating an oxonium ion as the reactive intermediate. Thus, a *Bronsted* acid would be available, which needs traces of water provided by air humidity to hydrolyze the primary TBS ether, followed by *in situ* ring closure via auxiliary cleavage. Attempts to add 1 equiv of water at −78 °C accelerated the reaction; however, partly the secondary TBS ether of **7** was also cleaved. A similar effect was observed when air humidity was used for a longer time. Due to the high polarity of the β-hydroxy-δ-lactone, most of it could not be recovered during the workup. This procedure is fast and economical; however, careful observation of the color change of the solvent is necessary to stop the reaction at the optimal point. In addition, upscaling attempts to 5 mmol led to lower conversions and yield (54%). Therefore, it was replaced by a more reliable one.

The chiral lactone **7** obtained as described above was treated with crotonaldehyde based on Brückner et al.’s model study. Although this approach could probably lead to isomer **3**, we performed this second aldol step to check whether their method was applicable for our purpose. As they used methyl-substituted lactones, we adjusted their method to our TBS-protected lactone **7** by reducing the boron reagent (1.5–1.1 equiv) and the basic reagent (1.6–1.3 equiv), respectively.^14^

Product **8** with the chiral quaternary center in the α-position was obtained in 81% yield (dr ≥ 99:1) in crystalline form. The absolute structure was proven by X-ray crystallography (Figure 2), revealing that the chiral quaternary center had an (*R*)-configuration like the non-natural isomer **3**. However, we recognized that the desired stereochemical triad in **8** was also generated with the disubstituted α-methyl-β-TBS-protected lactone **7**.

**Figure 2:**
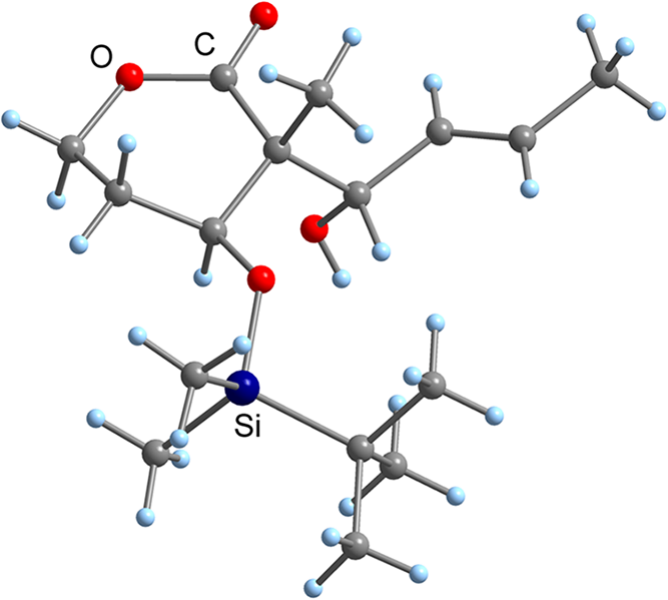
Molecular structure of lactone **8** in the crystal.

After protection with chloro­(methoxy)­methane (MOMCl), chiral lactone **9** was opened to obtain the final non-natural isomer **3** in 68% yield over two steps. For transesterification of **9**, a mild method was required to avoid the cleavage of the protecting groups. Hence, we developed a reliable method based on known and often used reagents like potassium hydroxide (KOH) to open the lactone, followed by *in situ* esterification with trimethylsilyl diazomethane (TMSCH_2_N_2_) (Scheme 1).^11^ The procedure was successful between 0 °C and room temperature in a dry organic solvent mixture (THF/methanol 3:1) that dissolves KOH and further reagents. Furthermore, the basic reaction environment was almost neutralized *in situ* with a calculated quantity of water-free camphersulfonic acid (CSA), avoiding acidification. In this way, traces of the product were sacrificed to prevent a reverse reaction. In addition, diethyl ether was added to change the polarity, and potassium sulfonate precipitated. Finally, the resulting carboxylic acid was immediately esterified with trimethylsilyl diazomethane in a suitable solvent mixture as described before.^15^ It should be pointed out that the disadvantage of this method is the use of trimethylsilyl diazomethane in excess.

The experiments described above offered an impressive method for the stereoselective construction of a chiral all-carbon quaternary center in the α-position to an ester group with an (*R*)-configuration and two individually protected β-hydroxy groups.

Next, we inverted the configuration of the quaternary center by changing the sequence of the aldehydes used in order to obtain isomer **2**. Based on our experience so far, the synthesis of isomer **2** followed again the strategy of two consecutive aldol steps with a lactone intermediate.

For reasons of atom economy, we used a TBS-protected β-hydroxybutanal for the first aldol step in order to provide a precursor for the allylic part of isomer **2**. However, this strategy had the consequence that a trisubstituted lactone was formed as an intermediate between the first and the second aldol steps. The lactone had a methyl group in δ-position as the third substituent, which can have an influence on the stereoselectivity obtained.^14^ Because of the known influence of the δ-methyl group on a subsequent aldol step of a monosubstituted lactone, we could not predict the outcome. Therefore, we worked with two possible lactones with an (*R*)- and (*S*)-configuration in δ-position, respectively.

Thus, the first aldol reaction shown in Scheme 2 was performed by coupling (*S*)-*N-*propionyl-thiazolidinethione **4** with either TBS-protected (*S*)*-*β-hydroxybutanal **10** or (*R*)-β-hydroxybutanal **11** to achieve the non-Evans *syn* aldol products **12** and **13** with yields around 80% (dr >95:5) in both cases.^13^ Interestingly, compound **13** crystallized and could be analyzed by X-ray diffraction to confirm its absolute stereochemistry; the other diastereomer **12** remained oily.

Since the accidentally observed one-pot procedure described above (Scheme 1) was not so reliable, we switched to a more solid approach, which takes more time (48 h) but worked more straightforward without surveillance.^11^ Now the chiral α-methyl-β-TBS-ether-δ-methyl-lactones **14** and **15** were formed by an intramolecular lactonization with *p-*toluenesulfonic acid monohydrate and subsequent *in situ* protection of the β-hydroxy group with TBSCl/imidazole. Solid crystalline products were obtained in about 80% yield. However, only lactone **14** formed single crystals suitable for X-ray diffraction analysis. For this reason, unambiguous confirmation of the absolute configuration was possible only for compounds **13** and **14** (Figures S2 and S3).

In order to perform the second aldol step via a boron enolate, we treated lactones **14** and **15** with a *para*-methoxybenzyl (PMB) protected propanal **16** according to our adjusted method. In this way, we could achieve compounds **17** and **18** with 78% (dr = 86:14) and 64% (dr = 80:20) yield, respectively. The structures could be confirmed by X-ray crystallography after deprotection of aldol products **17** and **18** with *tetra*-butylammonium fluoride (TBAF) to gain two crystalline diols **20** and **21** (Figure 3a,b). X-ray diffraction analyses revealed the relative configurations of the achieved stereochemistry and especially the chiral quaternary carbon centers. The correct stereochemistry of compounds **20** and **21** could be determined by the use of compounds **13** and **14** whose absolute configuration was known. Lactone **17** represented a potential key intermediate for the total synthesis of (+)-disorazole Z1. However, lactone **18** seemed to be useless for our aim.

**Figure 3:**
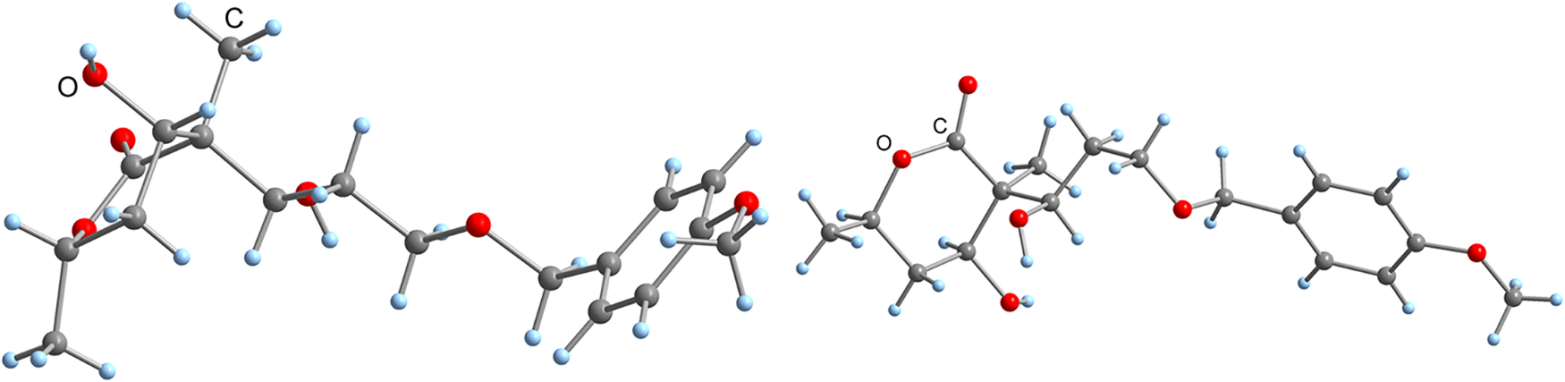
(a) Molecular structure of diol **20** in the crystal (left). (b) Molecular structure of diol **21** in the crystal (right).

Consequently, the use of (*S*)-butanal **10** in the first aldol step was necessary to construct the correct configuration of the quaternary carbon center (Figure 3a) represented in the natural isomer **2**. In addition, our work proved and extended the work of Brückner et al., who showed that the δ-methyl group of a lactone has an influence on the stereochemical outcome of the subsequent aldol step. We discovered that even in the case of a trisubstituted lactone, the methyl group in δ-position controls the diastereoselectivity even when a large TBS group has been placed in β-position close to the reaction center.

Next, lactone **17** was protected with triethylsilyl (TES) triflate and diisopropylethylamine (DIPEA), followed by ring opening and esterification as described above (Scheme 1). In this case, we could observe that even the labile TES protecting group was preserved, predominantly achieving compound **22** with 60% yield over two steps.

**Scheme 1:**
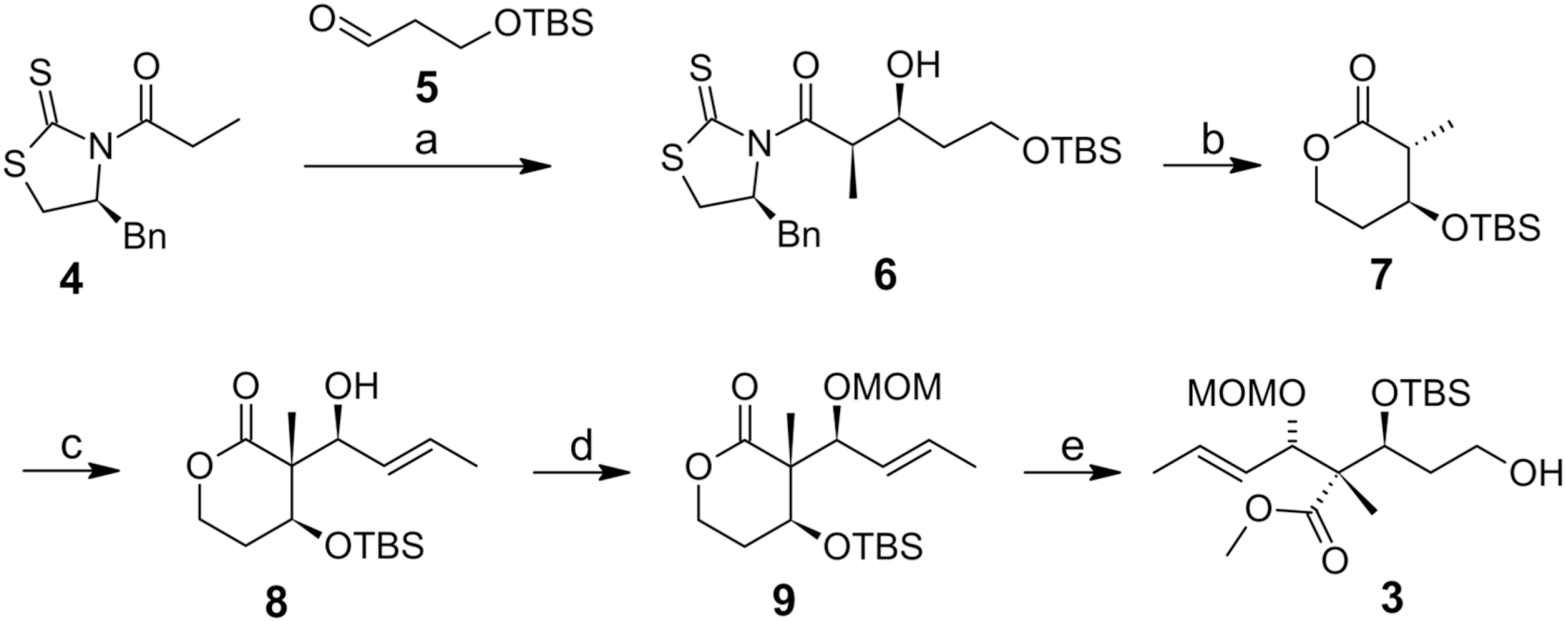
Synthesis of the non-natural lateral chain precursor **3** (a) TiCl_4_, DIPEA, aldehyde **5**, DCM, 1 h, −78 °C, 79%; (b) TBSOTf, air humidity, DCM, −78 °C → −16 °C, 2 h 70%; (c) 1 M Bu_2_BOTf in DCM, Et_3_N, crotonaldehyde, DCM, −78 °C, 4.5 h, 81%; (d) MOMCl, DIPEA, DCM, 7.5 h, 30 °C, 88%; (e) 1 M KOH in MeOH, CSA, TMSCH_2_N_2_, MeOH/THF/Et_2_O, 41 h, r.t., 77%.

Now, a Zaytsev elimination of the δ-hydroxy group of **22** leading to **2** had limited possibilities,^16^ as both the sensitive TES group and the methyl ester had to tolerate harsh conditions. Furthermore, mild alternatives like the *Burgess* reagent led to ring closing. Phosphoryl chloride (POCl_3_) in pyridine only replaced the hydroxy group with chloride.^17^ Additional attempts to form a leaving group with *para-*toluenesulfonyl chloride (*p-*TsCl) resulted in a highly unstable compound.^18^ Depending on the workup procedure, basic conditions led to starting material **22**, and acidic conditions gave rise to ring closing. Hence, any kind of further elimination reactions with bases like sodium hydride were obsolete.

Even avoiding a workup by the addition of diazabicycloundecene (DBU) and subsequent heating led predominantly to the formation of the chloride or ring closure. Only traces of elimination products could be observed. As a consequence, we assumed that the methyl ester blocked the elimination process due to a strong neighboring group effect.^19^ Thus, we had to change the strategy.

In a second attempt to form isomer **2** with the desired (*S*)-configuration of the quaternary carbon center, we wanted to exploit the effect of lactone **15** by adding crotonaldehyde, as shown in Scheme 2, hoping for the same stereochemical outcome that we obtained for compound **18**. We speculated that this time the quaternary center would have the desired stereochemical outcome; only the resulting stereocenter in the β-position would have to be inverted using a Mitsunobu reaction. Indeed, the aldol addition could be performed successfully with a yield of 54% (dr = 80:20). Compound **19** crystallized and was accessible to X-ray structure analysis (Figure 4). The analysis revealed a structure similar to that of lactone **8**. Obviously, in our case, the usage of an unsaturated aldehyde did not lead to the same stereochemical outcome as a saturated aldehyde.

**Scheme 2:**
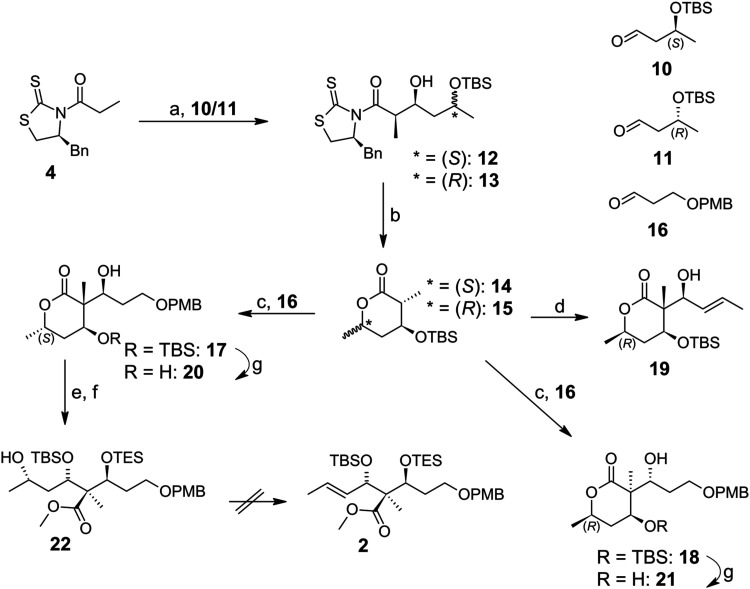
Attempts to synthesize the natural lateral chain precursor **2** (a) TiCl_4_, DIPEA, aldehyde **10** or **11**, DCM, 1 h, −78 °C, **12** = 81%/**13** = 79%; (b) *p-*TsOH·H_2_O, DCM, 0 °C → r.t., 23 h, then TBSCl/imidazole, 0 °C → r.t. 25 h, **14** = 81%/**15** = 80%; (c) 1 M Bu_2_BOTf, Et_3_N, aldehyde **16**, DCM, −78 °C, 4.5 h, **17** = 78%/**18** = 64%; (d) 1 M Bu_2_BOTf, Et_3_N, crotonaldehyde, DCM, −78 °C, 4.5 h, 54%; (e) TESOTf, DIPEA, DCM, −78 °C, 2 h, 88%; (f) 1 M KOH in MeOH, CSA, TMSCH_2_N_2_, MeOH/THF/Et_2_O, 41 h, r.t., 68%; (g) 1 M TBAF in THF, THF, 0 °C, 1 h, **20** = 31%/**21** = 55%.

**Figure 4:**
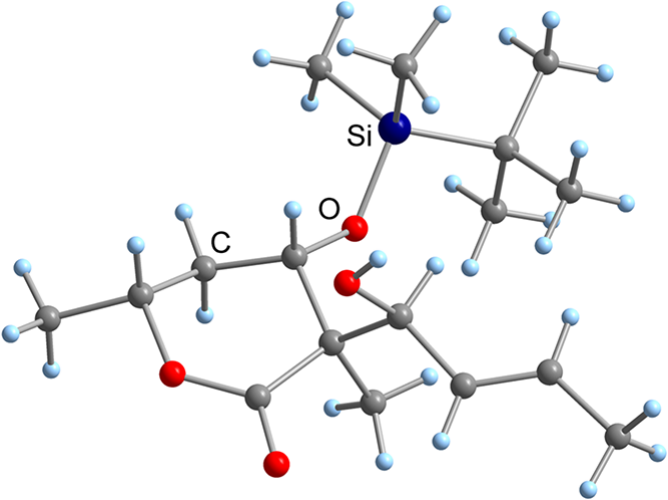
Molecular structure of lactone **19** in the crystal.

Therefore, we returned to a less atom economic strategy (Scheme 3), which was already shown to be the most successful route for the total synthesis of (+)-disorazole Z1.^11^ Lactone **7** generated according to the reliable method shown in Scheme 2 was reacted with the PMB-protected aldehyde **16** forming lactone **23** as the TES-protected compound over two steps. Then, our mild lactone transesterification method was also applied here, followed by a DMP oxidation and a Wittig reaction to form a terminal alkene **24**. Finally, terminal alkene **24** was isomerized to the final compound **2** with an *E*/*Z* ratio of 12:1 according to Hanessian et al. using Grubbs II reagent.^20^ This method had to be adjusted to our intermediate by reducing the temperature from 60 to 55 °C because the conversion stopped at 60 °C! In addition, it should be mentioned here that normal lab-grade methanol is in fact sufficient, and the addition of 1 equiv of triethylamine ensures complete transformation of the Grubbs II reagent to the necessary isomerization catalyst without any interaction with our substrate.^21^ Side products were not observed either.

**Scheme 3:**
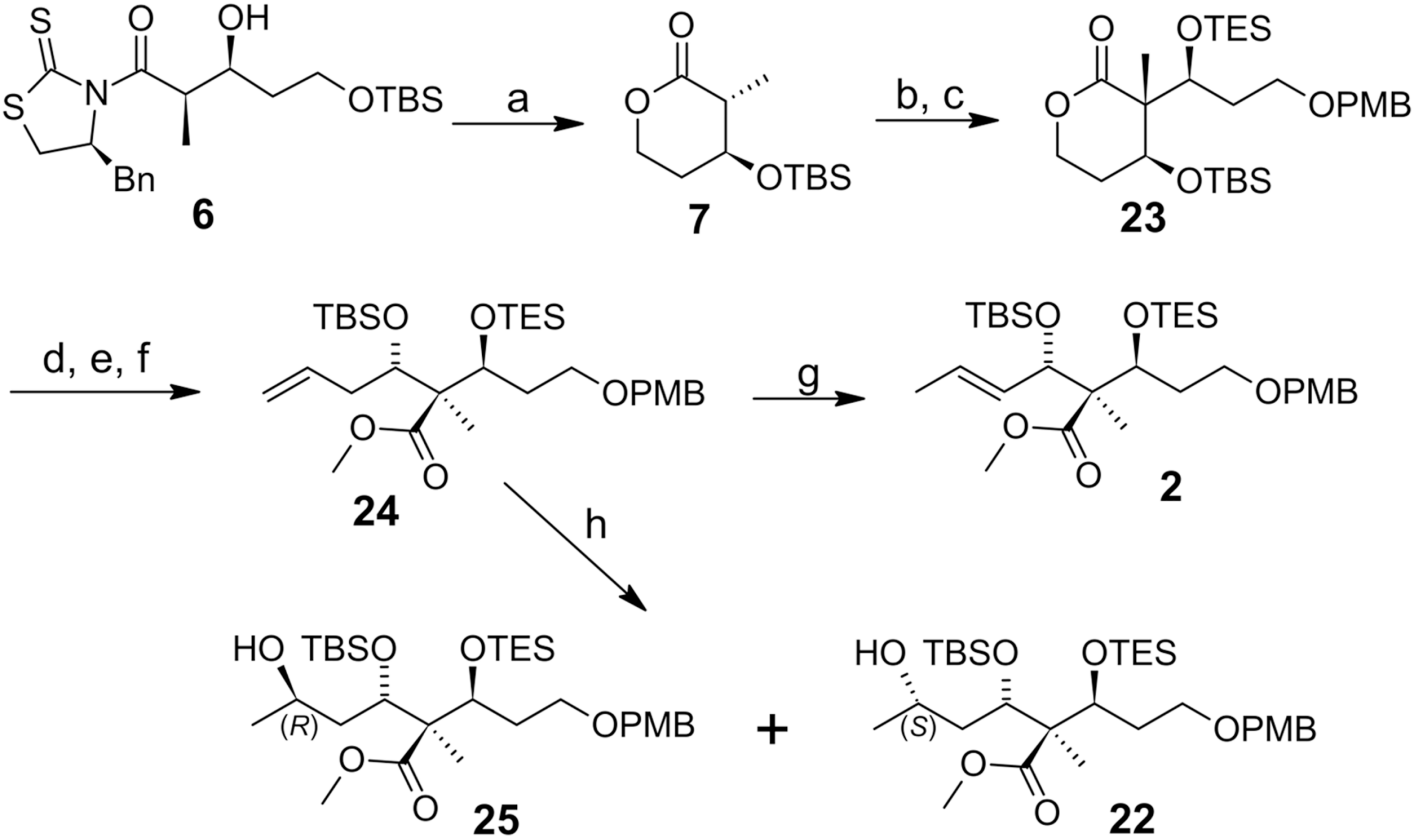
Final structure proof of the Recently Published Synthesized Natural Lateral Chain **2** ^a^

a (a) *p-*TsOH·H_2_O, DCM, 0 °C → r.t., 23 h, then TBSCl/imidazole, 0 °C → r.t. 25 h, 71%; (b) 1 M Bu_2_BOTf, Et_3_N, aldehyde **16**, DCM, −78 °C, 4.5 h; (c) TESOTf, DIPEA, DCM −78 °C, 2 h, 55% over two steps; (d) 1 M KOH in MeOH, CSA, TMSCH_2_N_2_, MeOH/THF/Et_2_O, 23 h, r.t., 70%; (e) DMP, NaHCO_3_, 0 °C → r.t.; (f) *n*-BuLi, MePPh_3_Br, THF, −78 °C → r.t., 6.5 h, 82% over two steps; (g) Grubbs II, Et_3_N, MeOH, 55 °C, 18 h, 90%, *E*/*Z* = 12:1;^11^ Final structure proof: (h) Hg­(OAc)_2_, NaBH_4_, water/THF, r.t., 21 h, 25%, *R*/*S* = 3:1.

Unfortunately, in this third approach, no crystalline products could be obtained after the second aldol step; even a diol derived from **23** did not crystallize. Therefore, the correct stereochemistry of compound **23** was not clear at that point. We made this assumption based on the structure of lactone **8**. This assumption was proved with the help of compound **22** when the terminal alkene **24** was treated with mercury­(II) acetate to hydrate the double bond via an oxymercuration reaction which generated an inseparable mixture of compounds **25** and **22** with an *R*/*S* ratio of 3:1 of the newly formed δ-hydroxy group in 25% yield.^22^ A comparison of the ^13^C­{^1^H}-NMR spectrum of mixture **25**/**22** with compound **22**, whose structure was proven by X-ray crystallography of diol **20** (Scheme 2), confirmed that we had obtained the desired isomer **2**. Indeed, later on, it was also proven by the successful total synthesis of **1**.^11^

## Conclusions

In this account, we showed numerous approaches to build up chiral quaternary carbon centers together with vicinal stereocenters, also described as chiral acyclic α-quaternary-β,β′-hydroxy esters. We unambiguously demonstrated the advantage of lactone intermediates for the diastereoselective construction of chiral quaternary carbon centers via consecutive aldol steps. We also developed an alternative lactonization procedure, provided detailed fine adjustments of lactone ring opening and subsequent esterification preserving predominantly the sensitive TES protecting group as well as the methyl ester by preventing it from relactonization.

In addition, Scheme 2 shows a very atom-economical way to synthesize several types of stereochemical triads. Although, this strategy could not provide isomer **2**, it reveals two important points: (i) the position of a δ-methyl group of a trisubstituted lactone controls the following aldol reaction and (ii) while going two ways to one aim results of single-crystal X-ray structural analysis obtained for intermediates **13**, **14**, **17**, and **18** during one procedure could be applied to reinforce the stereochemistry of compound **24** of the other procedure. This had been used successfully for the total synthesis of (+)-disorazole Z1 at that time with some risk because there was no clear evidence that compound **24** was suitable.

Hence, we developed strategies to synthesize quaternary carbon centers with an (*R*)- or (*S*)-configuration, respectively, even via polysubstituted lactone intermediates. Therefore, the results obtained in this study appear also applicable for the synthesis of disorazole analogues and of other chiral quaternary carbon centers with functionalized side chains.

## Supplementary Material



## Data Availability

The data underlying this study are available in the published article and its Supporting Information.

## Notes

Experimental procedures, analytical data, NMR spectra, and all X-ray analyses. In addition, safety statements of significant hazards or risks may be found there. (PDF)

## References

[1] Wu G., Wu J. R., Yan Huang Y., Yang Y. W. (2021). Enantioselective
Synthesis of Quaternary Carbon Stereocenters by Asymmetric Allylic
Alkylation: A Review. Chem. – Asian J..

[2] Christoffers J., Baro A. (2005). Stereoselective Construction of Quaternary
Stereocenters. Adv. Synth. Catal..

[3] Ling T., Rivas F. (2016). All-carbon quaternary
centers in natural products and medicinal chemistry:
recent advances. Tetrahedron.

[4] Douglas C. J., Overman L. E. (2004). Catalytic asymmetric
synthesis of all-carbon quaternary
stereocenters. Proc. Natl. Acad. Sci. U.S.A..

[5] Long R., Huang J., Gong J., Yang Z. (2015). Direct construction
of vicinal all-carbon quaternary stereocenters in natural product
synthesis. Nat. Prod. Rep..

[6] Li C., Ragab S. S., Liu G., Tang W. (2020). Enantioselective formation
of quaternary carbon stereocenters in natural product synthesis: a
recent update. Nat. Prod. Rep..

[7] Gao Y., Birkelbach J., Fu C., Herrmann J., Irschik H., Morgenstern B., Hirschfelder K., Li R., Zhang Y., Jansen R., Müller R. (2023). The Disorazole Z Family of Highly
Potent Anticancer Natural Products from Sorangium cellulosum: Structure,
Bioactivity, Biosynthesis and Heterologous Expression. Microbiol. Spectrum.

[8] Irschik, H. ; Jansen, R. ; Sasse, F. Biologically Active Compounds Obtainable from *Sorangium cellulosum* . European Patent EP1743897A1, 2005.

[9] Schäckel R., Hinkelmann B., Sasse F., Kalesse M. (2010). The Synthesis of novel
disorazoles. Angew. Chem..

[10] Bold C. P., Lucena-Agell D., Oliva M. Á., Diaz J. F., Altmann K. H. (2022). Synthesis
and biological evaluation of C(13)/C(13′)-Bis­(desmethyl)­disorazole
Z. Angew. Chem., Int. Ed..

[11] Bauer T. J., Aminian S., Spieß O., Schinzer D. (2025). Total synthesis of
(+)-Disorazole Z1. Chem. – Eur. J..

[12] Menchon G., Prota A. E., Lucena-Agell D., Bucher P., Jansen R., Irschik H., Müller R., Paterson I., Fernando
Diaz J. F., Altmann K. H., Steinmetz M. O. (2018). A fluorescence
anisotropy assay to discover and characterize ligands targeting the
maytansine site of tubulin. Nat. Commun..

[13] Crimmins M. T., King B. W., Tabet E. A., Chaudhary K. (2001). Asymmetric
Aldol Additions: Use of Titanium Tetrachloride and (−)-Sparteine
for the Soft Enolization of N-acyl Oxazolidinones, Oxazolidinethiones,
and Thiazolidinethiones. J. Org. Chem..

[14] Weber F., Becker F., Keller M., Hillebrecht H., Brückner R. (2015). Aldol Additions of Titanium and Boron
Enolates of Achiral
and Chiral δ-Lactones to Achiral Model Aldehydes: Simple and
induced Diastereoselectivities. Eur. J. Org.
Chem..

[15] Presser A., Hüfner A. (2004). Trimethylsilyl diazomethane – A mild and efficient
reagent for the methylation of carboxylic acids and alcohols in natural
products. Monatsh. Chem..

[16] Kostikov, R. R. ; Khlebnikov, A. F. ; Sokolov, V. V. Synthesis by Elimination Reactions. In Science of Synthesis; de Meljere, A. , Ed.; Thieme Verlag: Munich, 2010; pp 771–801.

[17] Hjerrild P., Torring T., Poulsen T. B. (2020). Dehydration
reactions in polyfunctional
natural products. Nat. Prod. Rep..

[18] Kabalka G. W., Varma M., Varma R. S., Srivastava P. C., Knapp F. F. (1986). Tosylation of alcohols. J. Org.
Chem..

[19] Clayden, J. ; Greeves, N. ; Warren, S. Organische Chemie; Springer Spektrum: Auflage, 2013; p 1023.

[20] Hanessian S., Giroux S., Larsson A. (2006). Efficient
Allyl to Propenyl Isomerization
in Functionally Diverse Compounds with a Thermally Modified Grubbs
2nd Generation Catalyst. Org. Lett..

[21] Dinger M.
B., Mol J. C. (2003). Degradation
of the Second-Generation Grubbs Metathesis
Catalyst with Primary Alcohols and Oxygen – Isomerization and
Hydrogenation Activities of Monocarbonyl Complexes. Eur. J. Inorg. Chem..

[22] Phillips S. T., Shair M. D. (2007). Syntheses of the Eastern Halves of Ritterazines B,
F, G, and H, Leading to Reassignment of the 5,5-Spiroketal Stereochemistry
of Ritterazines B and F. J. Am. Chem. Soc..

