# Supplementary material for: Development of a Stereoselective Synthesis of Isomers of (+)-Disorazole Z1’s Lateral Chain
Source: J Org Chem. 2025 Oct 29;90(44):15648–53. doi: 10.1021/acs.joc.5c01789 (PMC12604028; doi:10.1021/acs.joc.5c01789)
Supplement: Supplementary file 1 [file jo5c01789_si_001.pdf]

Supporting Information

Development of a Stereoselective Synthesis of Isomers of (+)-Disorazole Z1's Lateral Chain

*Thomas J. Bauer, Phil Köhler, Oliver Spieß, and Dieter Schinzer**

General……………………………………………………………………………………………S2
Experimental procedures and $^{1}H/^{13}C\{^{1}H\}$ NMR spectra……………………………………S3
X-ray data…………………………………………………………………………………………S44
References…………………………………………………………………………………………S48

General

Solvents were dried by standard procedures and redistilled under nitrogen atmosphere prior to use or were purchased in an appropriate water free quality and used as obtained. All reactions were run under nitrogen unless otherwise stated. For reactions that require heating, an oil bath was used. The products were purified by flash chromatography on Merck silica gel 60 (40-63 µm). POLYGRAM SIL G/UV254 prefabricated TLC plates with fluorescent indicator from Macherey-Nagel have been used for the analytical thin layer chromatography (TLC). The separated substances were detected by irradiation with UV light with a wavelength of 254 nm or staining with vanillin or potassium permanganate reagent and subsequent warming with a heat gun. Electrospray ionization (ESI) and electron ionisation (EI) mass spectra were recorded on Finnigan MAT 95 and Waters Xevo G2-TOF spectrometers. ^{1}H and $^{13}C\{^{1}H\}$ NMR spectra were recorded on Brucker AVIII 400 and Brucker AVI 600 spectrometers. Chemical shifts (δ) are reported in ppm from tetramethylsilane, referenced to the solvent resonance resulting from incomplete deuteration (^{1}H NMR = $CDCl_3$: 7.26, C_6D_6: 7.16; $^{13}C\{^{1}H\}$ NMR = $CDCl_3$: 77.16, C_6D_6: 128.06). Data are reported as follows: chemical shift, multiplicity (s = singlet, d = doublet, t = triplet, q = quartet, quint = quintet, br = broad, m = multiplet), integration and coupling constants (Hz). Optical rotations were recorded on an Anton Raar MCP150 polarimeter. Infrared (IR) spectra were recorded on Brucker Vertex 70v Bands are characterized as strong (s), medium (m), weak (w) or broad (br).

(3*R*,4*S*)-4-((*tert*-butyl(dimethyl)silyl)oxy)-3-methyloxan-2-one (7)

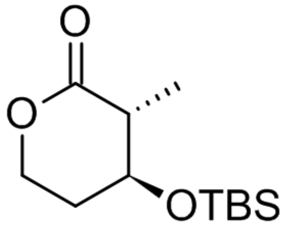

7

(2*R*,3*S*)-1-((4*S*)-4-benzyl-2-thioxo-1,3-thiazolidin-3-yl)-5-((*tert*-butyl(dimethyl)silyl)oxy)-3-hydroxy-2-methylpentan-1-one (**6**) (for preparation see ref. S1) (453 mg, 1.0 mmol, 1.0 eq.) is dissolved in anhydrous dichloromethane (5 ml). After cooling to -78°C TBSOTf (0.24 ml, 1.05 mmol, 1.05 eq.) is added and the solution is stirred further 15 min at the same temperature. Then the reaction is allowed to warm up to -16°C over 2h with open flask. Air humidity triggers the reaction which can be recognized by color change of the solvent from yellow to colorless. The reaction is quenched by addition of an aqueous saturated solution of $NaHCO_3$ (7 ml) under vigorous stirring. The organic layer is separated and the aqueous layer is extracted with dichloromethane (3x7 ml). The combined organic layers are dried over $MgSO_4$, filtered and the solvent is removed under reduced pressure. The residue is purified by column chromatography (*n*-pentane/Et_2O, 6:1) to obtain lactone **7** (170.1 mg, 0.70 mmol, 70 %) as a colorless crystalline solid.

^{1}H NMR ($CDCl_3$, 400 MHz): δ 4.48 (ddd, 1H, *J* = 11.3, 8.9, 4.0 Hz), 4.23 (ddd, 1H, *J* = 11.3, 5.7, 4.7 Hz), 3.79 (td, 1H, *J* = 6.3, 4.5 Hz), 2.54 (quint, 1H, *J* = 7.1 Hz), 2.12 (ddt, 1H, *J* = 14.2, 9.0, 4.6 Hz), 1.80 (dtd, 1H, *J* = 14.3, 5.8, 4.0 Hz), 1.29 (d, 3H, *J* = 7.2 Hz), 0.89 (s, 9H), 0.08 (s, 3H), 0.07 (s, 3H).

^{13}C{^{1}H} NMR ($CDCl_3$, 101 MHz): δ 174.1, 70.3, 64.9, 44.7, 31.4, 25.8, 18.0, 14.7, -4.4, -4.7.

Analytical data as described in the literature.[S1]

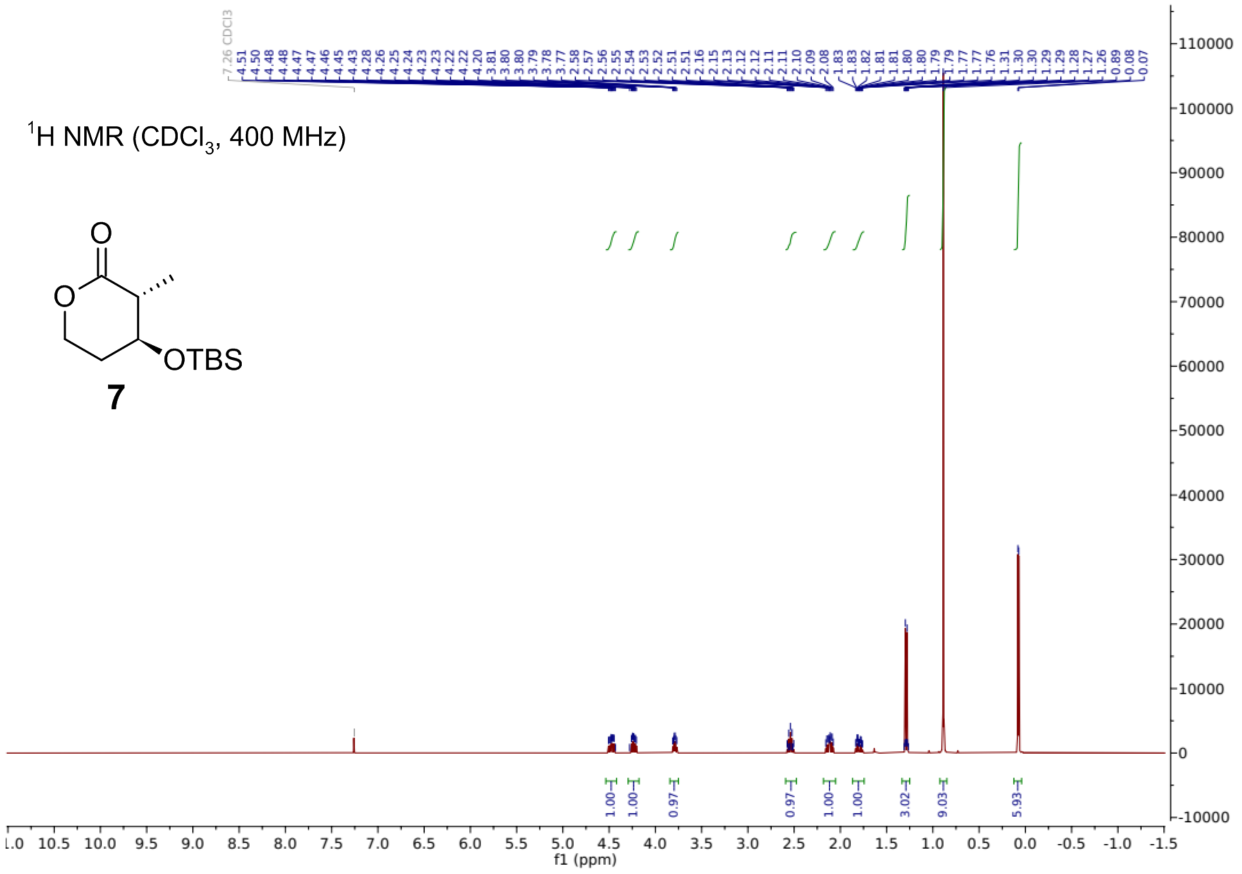

1H NMR (CDCl3, 400 MHz)
OTBS
7
7.26 CDCl3
4.51 4.50 4.48 4.48 4.47 4.46 4.45 4.43 4.28 4.26 4.25 4.24 4.23 4.23 4.22 4.22 4.20 3.81 3.80 3.80 3.79 3.78 3.77 2.58 2.57 2.56 2.55 2.54 2.53 2.52 2.51 2.51 2.16 2.15 2.13 2.12 2.12 2.11 2.11 2.10 2.09 2.08 1.83 1.83 1.82 1.81 1.81 1.80 1.80 1.79 1.79 1.77 1.77 1.76 1.31 1.30 1.30 1.29 1.28 1.27 1.26 0.89 0.08 0.07
1.00 1.00 0.97 0.97 1.00 1.00 3.02 9.03 5.93
f1 (ppm)

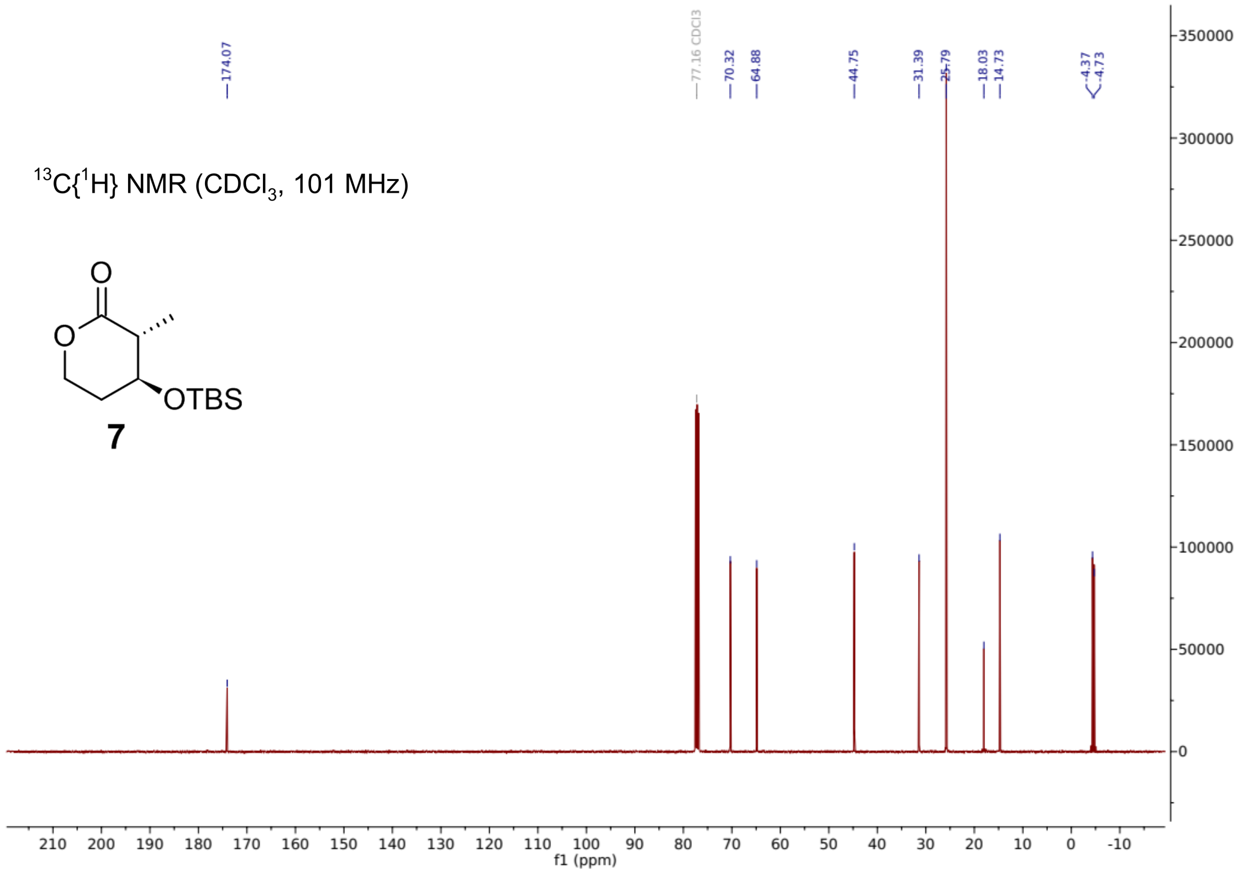

13C{1H} NMR (CDCl3, 101 MHz)
OTBS
7
174.07
77.16 CDCl3
70.32
64.88
44.75
31.39
25.79
18.03
14.73
-4.37
-4.73
f1 (ppm)

(3*R*,4*S*)-4-((*tert*-butyl(dimethyl)silyl)oxy)-3-((1*S*,2*E*)-1-hydroxybut-2-en-1-yl)-3-methyloxan-2-one (8)

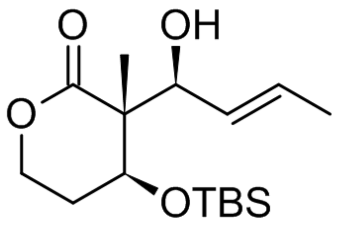

8

To a solution of lactone **7** (488 mg, 2.0 mmol, 1.0 eq.) in dry dichloromethane (9.2 ml) 1M Bu_2BOTf in dichloromethane (2.2 ml, 2.2 mmol, 1.1 eq.) is injected at -78 °C under nitrogen atmosphere. After stirring of the yellow solution for 20 min at -78 °C triethylamine (0.36 ml, 2.6 mmol, 1.3 eq.) is added slowly which leads to a colorless solution. The reaction mixture is allowed to stir 1 h at this temperature. Then a mixture of crotonaldehyde (0.33 ml, 4.0 mmol, 2.0 eq.) with anhydrous dichloromethane (0.67 ml) is added slowly dropwise within 20 min. After stirring of the colorless solution for 3 h at -78 °C the cold reaction mixture is quenched sequentially with 20 mM aqueous phosphate buffer (8 ml), methanol (4 ml) and 30 % H_2O_2 (2 ml) at -78 °C. The cooling bath is removed and vigorous stirring is continued for additional 1.5 h. Dichloromethane (10 ml) is added and the organic layer is separated. The aqueous layer is extracted with dichloromethane (3x20 ml) and the combined organic layers are washed once with an aqueous saturated Na_2SO_3 solution (11 ml). After drying over $MgSO_4$, filtration and removing of the solvent the residue is purified by column chromatography (*n*-pentane/Et_2O, 3:2) to afford product **8** (509 mg, 1.62 mmol, 81 %) as colorless crystalline solid.
In order to measure in X-ray, the crystalline solid is dissolved in freshly distilled diethyl ether. The solution is concentrated under reduced pressure until it becomes an oil which is covered immediately and carefully with freshly distilled *n*-pentane. Crystals start growing at room temperature while the solvent is allowed to evaporate slowly.

General data: R_f = 0.56 (*n*-pentane/Et_2O, 1:3), $[\alpha]^{20}_D$ = +1.610 (c=1.242, $CHCl_3$),
m. p. = 94 °C, MW = 314.49 g/mol.

IR (neat): 3540 (m), 2954 (m), 2929 (m), 2856 (m), 1715 (s), 1671 (w), 1252 (s), 1138 (m), 1106 (s), 1051 (m), 1027 (m), 1006 (m), 979 (m), 930 (m), 834 (s), 776 (s), 672 (m), 627 (m), 442 (m) cm^{-1}.

^{1}H NMR ($CDCl_3$, 400 MHz): δ 5.82 - 5.74 (m, 1H), 5.67 (dq, 1H, *J* = 15.3, 6.2 Hz), 4.43 (ddd, 1H, *J* = 11.1, 6.5, 4.6 Hz), 4.27 - 4.22 (m, 1H), 4.20 (dd, 1H, *J* = 8.5, 3.3 Hz), 4.12 (d, 1H, *J* = 7.9 Hz), 2.29 (s, 1H), 2.03 (dddd, 1H, *J* = 14.2, 6.5, 4.5, 3.4 Hz), 1.90 (dtd, 1H, *J* = 14.0, 8.2, 4.6 Hz), 1.72 (dd, 3H, *J* = 6.0, 1.2 Hz), 1.17 (s, 3H), 0.88 (s, 9H), 0.09 (s, 3H), 0.07 (s, 3H).

^{13}C{^{1}H} NMR ($CDCl_3$, 101 MHz): δ 175.5, 130.4, 129.7, 76.3, 68.8, 65.4, 53.7, 29.0, 25.8, 18.1, 17.9, 16.7, -4.1, -4.9.

HRMS (ESI): calculated for $C_{16}H_{30}O_4SiNa$ $[M+Na]^+$: 337.1811, found: 337.1818.

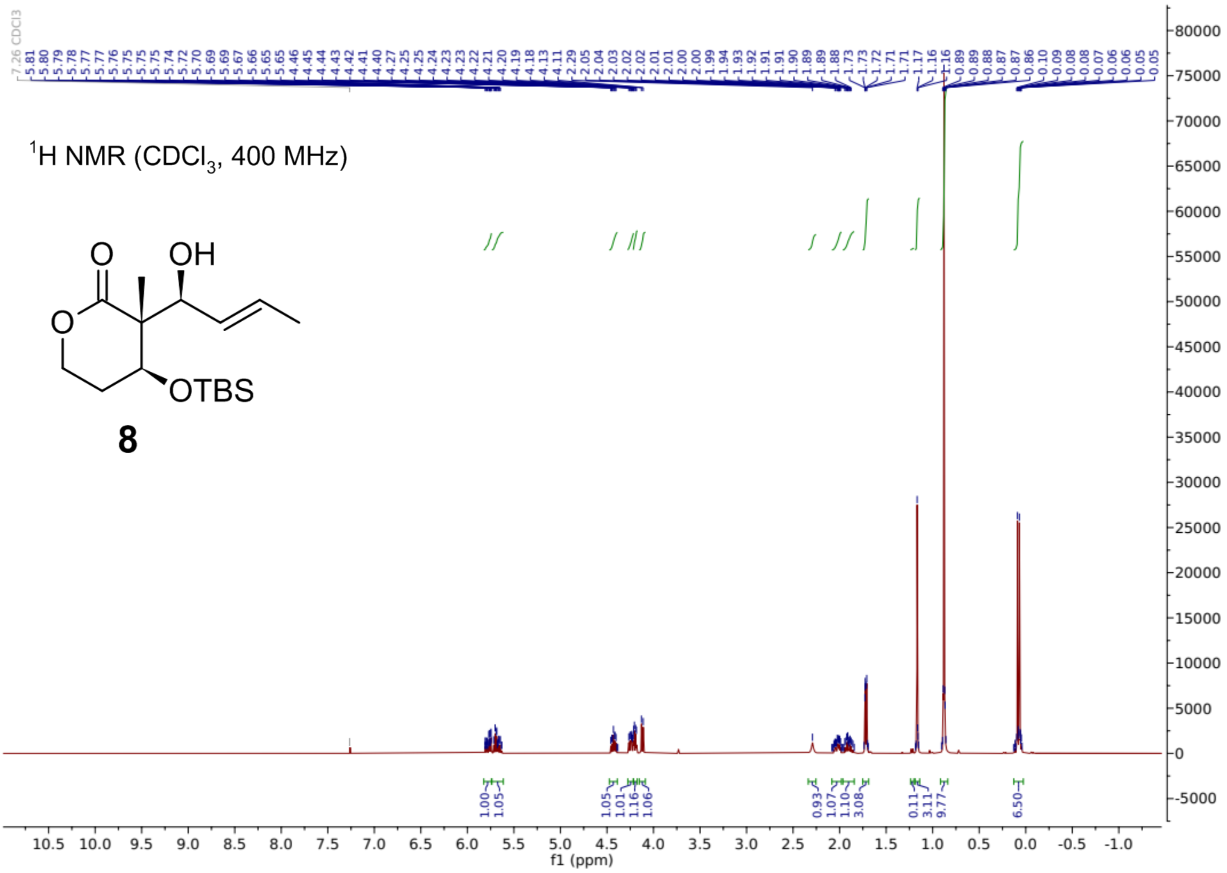

^{1}H NMR (CDCl$_3$, 400 MHz)
O
OH
O
OTBS
8
f1 (ppm)

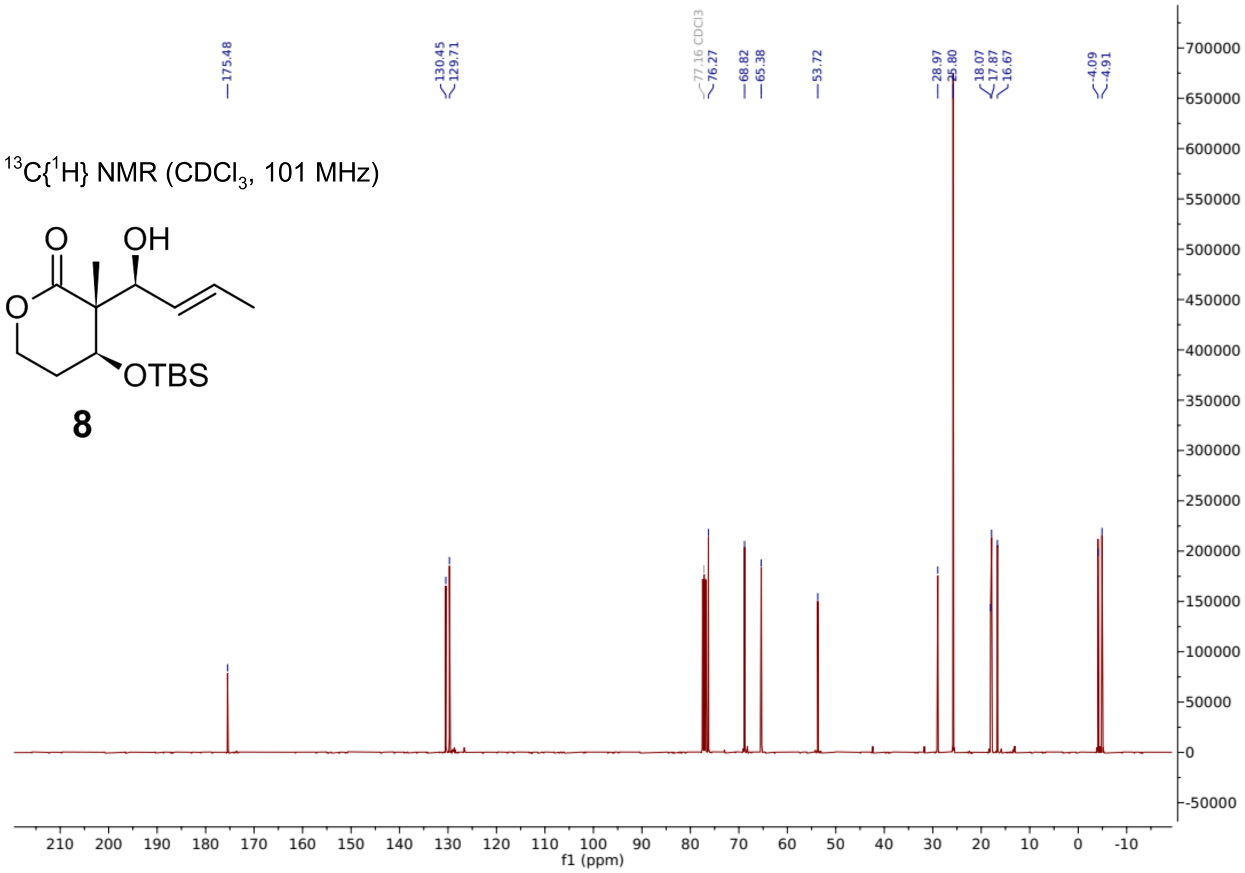

^{13}C{^{1}H} NMR (CDCl$_3$, 101 MHz)
O
OH
O
OTBS
8
175.48
130.45
129.71
77.16 CDCl3
76.27
68.82
65.38
53.72
28.97
25.80
18.07
17.87
16.67
-4.09
-4.91
f1 (ppm)

(3*R*,4*S*)-4-((*tert*-butyl(dimethyl)silyl)oxy)-3-((1*S*,2*E*)-1-(methoxymethoxy)but-2-en-1-yl)-3-methyloxan-2-one (9)

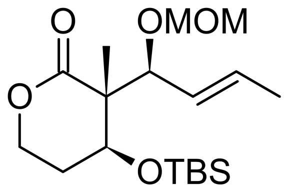

9

To a solution of compound **8** (430 mg, 1.37 mmol, 1.0 eq.) in anhydrous dichloromethane (*3*.8 ml) DIPEA (0.93 ml, 5.47 mmol, 4.0 eq.) and MOMCl (0.31 ml, 4.11 mmol, 3.0 eq.) are added sequentially at 0 °C. After warming to room temperature over 2 h the solution is stirred for 3.5 h at 30 °C, then 18 h at room temperature and after that again 4h at 30 °C. The orange mixture is quenched with water (6 ml) and the organic phase is separated. The aqueous layer is extracted with diethyl ether (3x10 ml) and the combined organic layers are dried over $MgSO_4$.. After filtration and concentration under reduced pressure the residue is purified by column chromatography (*n*-pentane/Et_2O, 2:1) to yield a product **9** (433 mg, 1.21 mmol, 88.3 %) as a colorless oil.

CAUTION! *MOMCl (chloromethyl methyl ether) is harmful and may cause cancer. We handled it using needle and syringe techniques under a well ventilated fume hood.*

General data: R_f = 0.43 (*n*-pentane/Et_2O, 1:1), $[\alpha]^{20}_D$ = +71.204 (c=0.382, $CHCl_3$),
MW = 358.55 g/mol.

IR (neat): 2953 (m), 2930 (m), 2891 (m), 2857 (m), 1731 (s), 1667 (w), 1256 (m), 1148 (m), 1111 (s), 1056 (m), 1031 (s), 1013 (s), 976 (m), 922 (m), 836 (s), 775 (s) cm^{-1}.

^{1}H NMR ($CDCl_3$, 400 MHz): δ 5.74 - 5.60 (m, 2H), 4.69 (d, 1H, *J* = 6.8 Hz), 4.46 (d, 1H, *J* = 6.8 Hz), 4.40 (ddd, 1H, *J* = 10.8, 6.2, 4.4 Hz), 4.25 (ddd, 1H, *J* = 11.1, 8.4, 4.0 Hz), 4.18 (dd, 1H, *J* = 8.6, 3.5 Hz), 4.07 (d, 1H, *J* = 8.0 Hz), 3.36 (s, 3H), 2.06 (ddt, 1H, *J* = 13.8, 6.2, 3.8 Hz), 1.88 (dtd, 1H, *J* = 13.9, 8.5, 4.4 Hz), 1.76 (d, 3H, *J* = 4.9 Hz), 1.17 (s, 3H), 0.90 (s, 9H), 0.11 (s, 3H), 0.08 (s, 3H).

^{13}C{^{1}H} NMR ($CDCl_3$, 101 MHz): δ 174.5, 132.6, 127.3, 93.50, 81.20, 70.0, 65.1, 56.1, 53.7, 29.8, 25.8, 18.2, 17.9, 17.8, -4.2, -4.8.

HRMS (ESI): calculated for $C_{18}H_{35}O_5Si$ $[M+H]^+$: 359.2254, found: 359.2258.

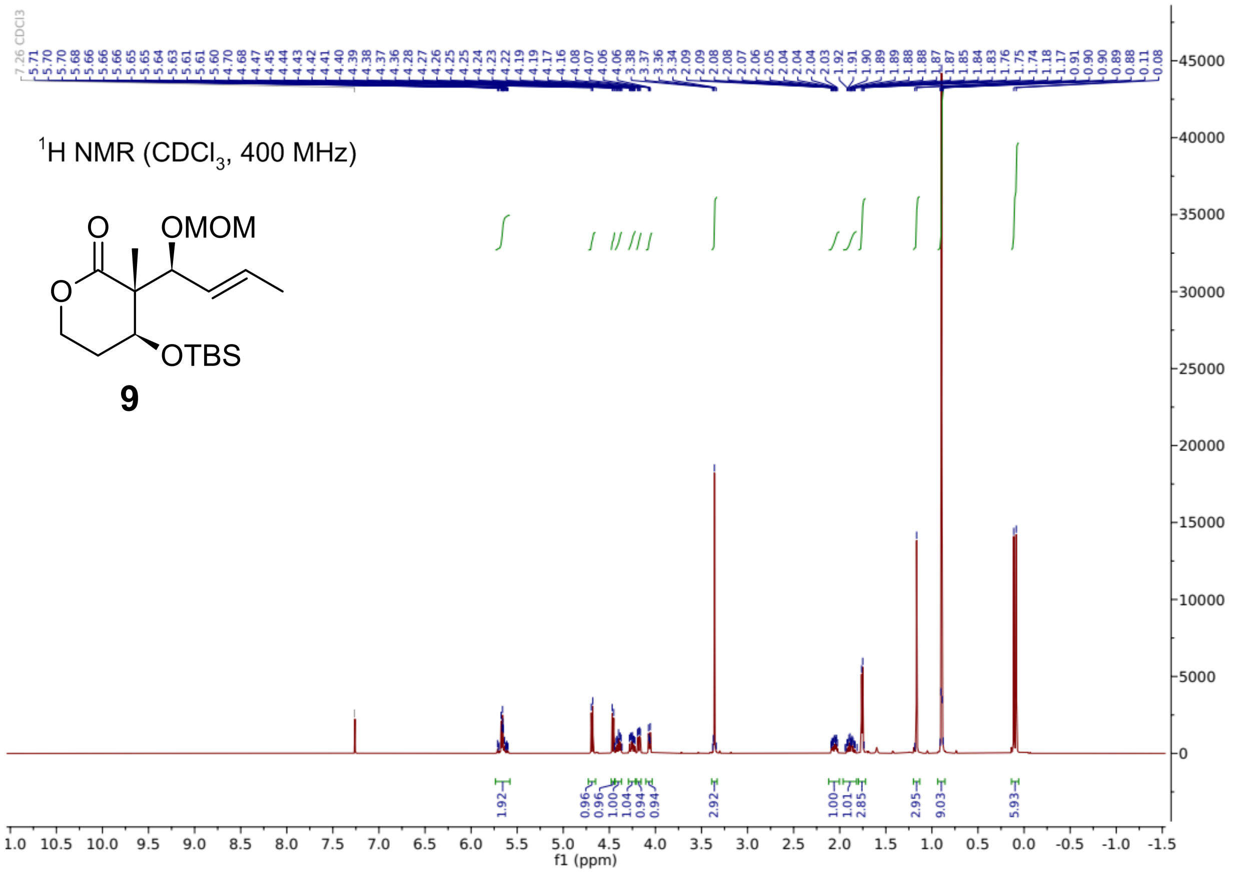

1H NMR (CDCl3, 400 MHz)
O
OMOM
O
OTBS
9
1.92
0.96
0.96
1.00
1.04
0.94
0.94
2.92
1.00
1.01
2.85
2.95
9.03
5.93
f1 (ppm)

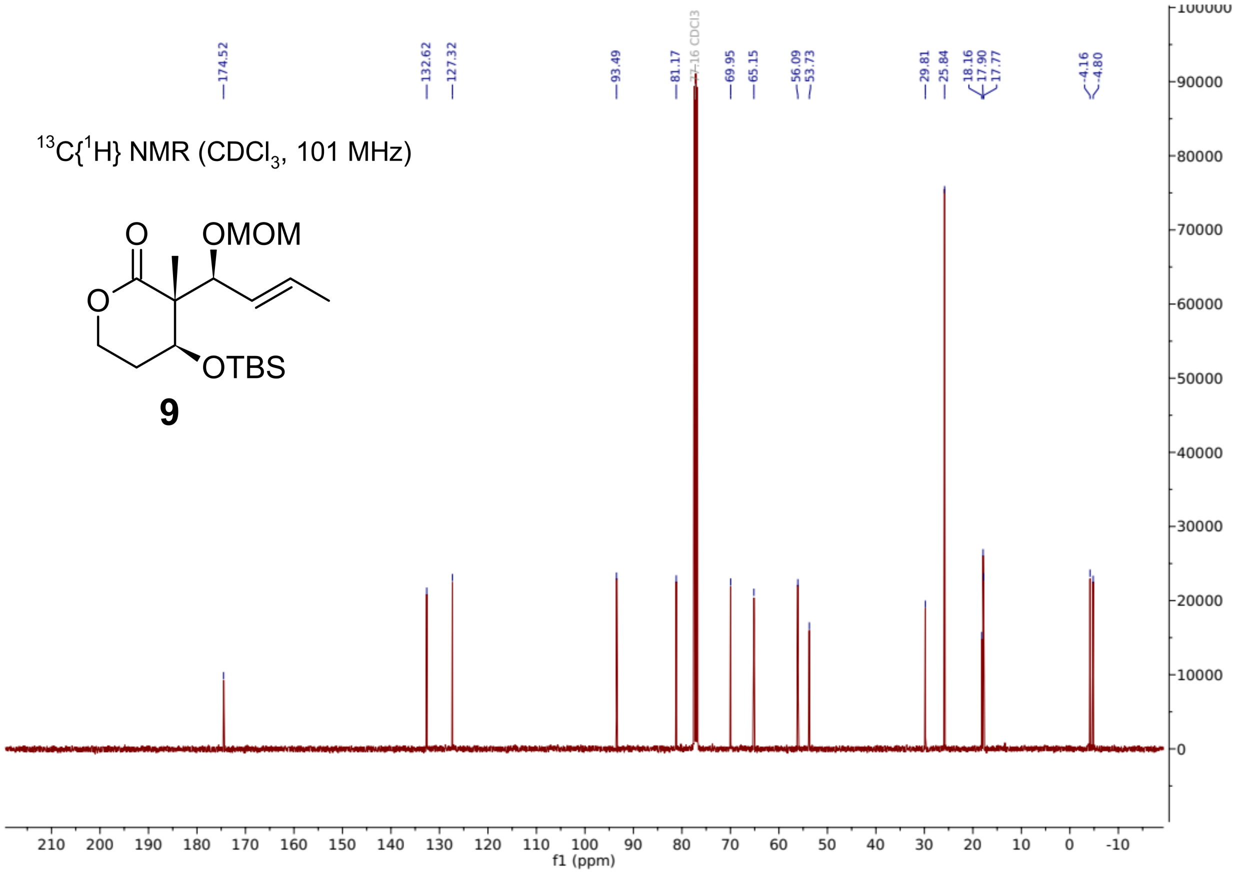

174.52
132.62
127.32
93.49
81.17
77.16 CDCl3
69.95
65.15
56.09
53.73
29.81
25.84
18.16
17.90
17.77
-4.16
-4.80
13C{1H} NMR (CDCl3, 101 MHz)
O
OMOM
O
OTBS
9
f1 (ppm)

Methyl (2*R*,3*S*,4*E*)-2-((1*S*)-1-((*tert*-butyl(dimethyl)silyl)oxy)-3-hydroxypropyl)-3-(methoxymethoxy)-2-methylhex-4-enoate (3)

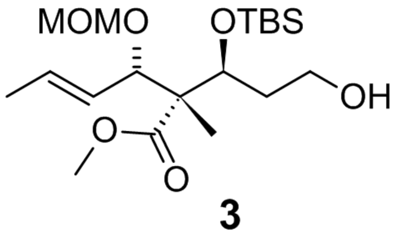

3

Note: The KOH solution in anhydrous methanol is prepared with 86.2 % potassium hydroxide pellets.

In a 10 ml pointed flask lactone **9** (75 mg, 0.21 mmol, 1.0 eq.) is dissolved in anhydrous THF (0.13 ml) and anhydrous methanol (0.04 ml). A 1.02 M KOH solution in anhydrous methanol (0.30 ml, 0.306 mmol, 1.457 eq.) is added slowly at 0 °C and the reaction solution is allowed to stir for further 50 min at 0 °C. After stirring for 41 h at room temperature the clear bright yellow solution is monitored by TLC (*n*-pentane/Et_2O, 1:1), which shows full conversion. A solution of camphorsulfonic acid (70.9 mg, 0.305 mmol, 1.453 eq.) in anhydrous methanol (1.4 ml) is added slowly dropwise at 0 °C. After stirring for 15 min at 0 °C diethyl ether (3.3 ml) is added. Then the reaction mixture is allowed to stir further 15 min at 0 °C before it is warmed to room temperature. The reaction solution becomes a white suspension which must be stirred more vigorous to keep it homogeneous. $TMSCH_2N_2$ solution in hexane is injected in several portions until TLC shows full conversion of the carboxylic acid intermediate (R_f: 0.1 (*n*-pentane/Et_2O, 1:1). Excess of $TMSCH_2N_2$ represented by a yellow suspension must be destroyed by slowly dropwise addition of acetic acid glacial at 0 °C. To the resulting white suspension triethylamine (1.0 ml) is added immediately. After warm up the suspension, dichloromethane (10 ml) and a saturated aqueous $NaHCO_3$ solution (1 ml) are poured in a separating funnel. After shaking water (1 ml) is added and the organic layer is separated. The aqueous layer is extracted with dichloromethane (3x10 ml) and the combined organic layers are dried over $MgSO_4$, filtered and concentrated under reduced pressure. The residue is purified by column chromatography (*n*-pentane/Et_2O, 2:1) to afford product **3** (63 mg, 0.162 mmol, 77.2 %) as a colorless oil.

CAUTION! *$TMSCH_2N_2$ (trimethylsilyldiazomethane) is fatal, may cause cancer and causes damage to organs. Solutions in hexane (0.5M to 2M) were purchased. The solutions were handled using needle and syringe techniques under a well ventilated fume hood.*

General data: R_f = 0.21 (*n*-pentane/Et_2O, 1:1), $[\alpha]^{20}_D$ = +18.095 (c=0.315, $CHCl_3$), MW = 390.59 g/mol.

IR (neat): 3474 (w br), 2951 (m), 2931 (m), 2887 (m), 2857 (m), 1727 (s), 1670 (w), 1248 (s), 1149 (s), 1091 (s), 1054 (s), 1027 (s), 972 (s), 834 (s), 774 (s) cm^{-1}.

^{1}H NMR (C_6D_6, 400 MHz): δ 5.65 (dqd, 1H, *J* = 15.4, 6.5, 0.7 Hz), 5.37 (ddq, 1H, *J* = 15.4, 8.8, 1.6 Hz), 4.73 (d, 1H, *J* = 6.6 Hz), 4.68 (d, 1H, *J* = 8.9 Hz), 4.45 (d, 1H, *J* = 6.6 Hz), 4.00 (dd, 1H, *J* = 7.8, 2.9 Hz), 3.62 - 3.51 (m, 2H), 3.47 (s, 3H), 3.25 (s, 3H), 2.06 - 1.87 (m, 2H), 1.52 (s, 3H), 1.50 (dd, 3H, *J* = 6.5, 1.7 Hz), 1.01 (s, 9H), 0.14 (s, 3H), 0.11 (s, 3H).

^{13}C{^{1}H} NMR (C_6D_6, 101 MHz): δ 174.2, 132.8, 126.7, 93.4, 78.9, 73.7, 59.6, 56.4, 55.7, 51.2, 36.9, 26.4, 18.7, 17.9, 15.8, -3.4, -3.8.

HRMS (ESI): calculated for $C_{19}H_{38}O_6SiNa$ $[M+Na]^+$: 413.2335, found: 413.2349.

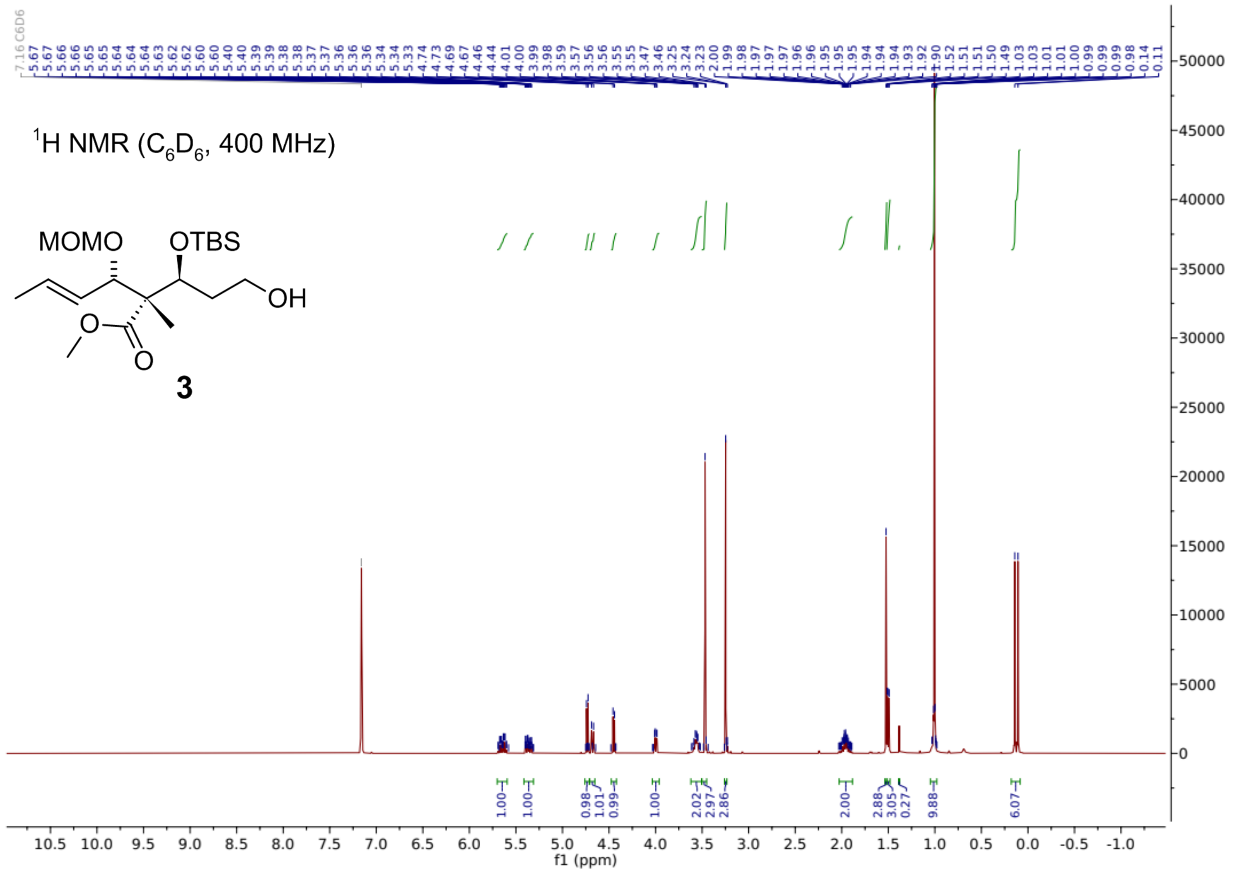

1H NMR (C6D6, 400 MHz)
MOMO
OTBS
OH
O
O
3
7.16 C6D6
5.67 5.67 5.66 5.65 5.65 5.64 5.64 5.63 5.62 5.62 5.60 5.60 5.40 5.40 5.39 5.39 5.38 5.38 5.37 5.37 5.36 5.36 5.34 5.34 5.33 4.74 4.73 4.69 4.67 4.46 4.44 4.01 4.00 3.99 3.98 3.59 3.57 3.56 3.56 3.55 3.55 3.47 3.46 3.25 3.24 3.23 2.00 1.99 1.98 1.97 1.97 1.97 1.96 1.96 1.95 1.95 1.95 1.95 1.94 1.94 1.94 1.93 1.92 1.90 1.52 1.51 1.51 1.50 1.49 1.03 1.03 1.01 1.01 1.00 0.99 0.99 0.98 0.14 0.11
1.00 1.00 0.98 1.01 0.99 1.00 2.02 2.97 2.86 2.00 2.88 3.05 0.27 9.88 6.07
f1 (ppm)

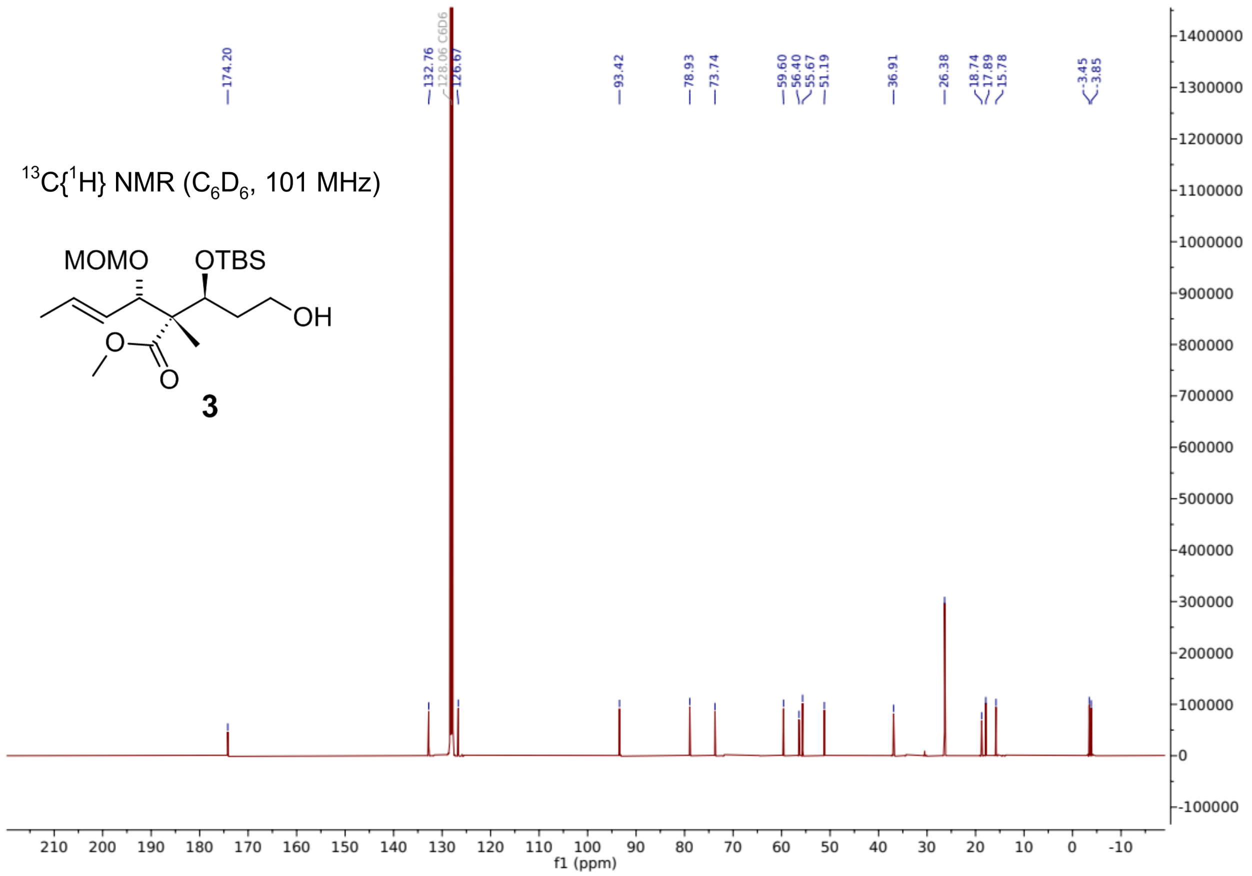

13C{1H} NMR (C6D6, 101 MHz)
MOMO
OTBS
OH
O
O
3
174.20 132.76 128.06 C6D6 126.67 93.42 78.93 73.74 59.60 56.40 55.67 51.19 36.91 26.38 18.74 17.89 15.78 -3.45 -3.85
f1 (ppm)

Ethyl (3S)-3-((*tert*-butyl(dimethyl)silyl)oxy)butanoate (i-10)
Ethyl (3R)-3-((*tert*-butyl(dimethyl)silyl)oxy)butanoate (i-11)

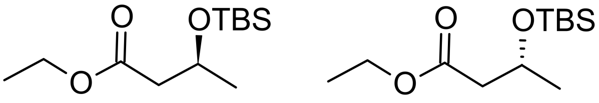

i-10 **i-11**

R or *S* isomer of ethyl 3-hydroxy-butanoate (2.62 ml, 20 mmol, 1.0 eq.) is dissolved in anhydrous dichloromethane (50 ml). After cooling of the solution to 0 °C imidazole (2.73 g, 40 mmol, 2.0 eq.) and TBSCl (3.62 g, 24 mmol, 1.2 eq.) are added sequentially. The white suspension is stirred for 24 h at room temperature. Then an aqueous saturated $NaHCO_3$ solution (30 ml) is poured in and the mixture is diluted with diethyl ether (300 ml). The organic phase is separated, washed with water (2x40 ml) and brine (50 ml). After drying over $MgSO_4$ and filtration of the organic layer, the solvent is removed under reduced pressure. In order to purify the crude material, a column chromatography (*n*-pentane/Et_2O 98:2) is done to afford product **i-10** or **i-11** (4.7 g, 19.1 mmol, 96 %) as a colorless oil.

General data: R_f = 0.52 (*n*-pentane/Et_2O, 95:5), **i-10**: $[\alpha]^{25}_D$ = +23.340 (c=0.994, $CHCl_3$), **i-11**: $[\alpha]^{25}_D$ = -24.203 (c=1.004, $CHCl_3$).

^{1}H NMR ($CDCl_3$, 400 MHz): δ 4.33 - 4.21 (m, 1H), 4.19 - 4.03 (m, 2H), 2.46 (dd, 1H, *J* = 14.5, 7.6 Hz), 2.35 (dd, 1H, *J* = 14.5, 5.3 Hz), 1.25 (t, 3H, *J* = 7.2 Hz), 1.18 (d, 3H, *J* = 6.1 Hz), 0.85 (s, 9H), 0.05 (s, 3H), 0.03 (s, 3H).

^{13}C{^{1}H} NMR ($CDCl_3$, 101 MHz): δ 171.8, 66.0, 60.4, 45.1, 25.9, 24.1, 18.1, 14.3, -4.4, -4.9.

Analytical data as described in the literature.[S2]

^{1}H NMR ($CDCl_3$, 400 MHz)

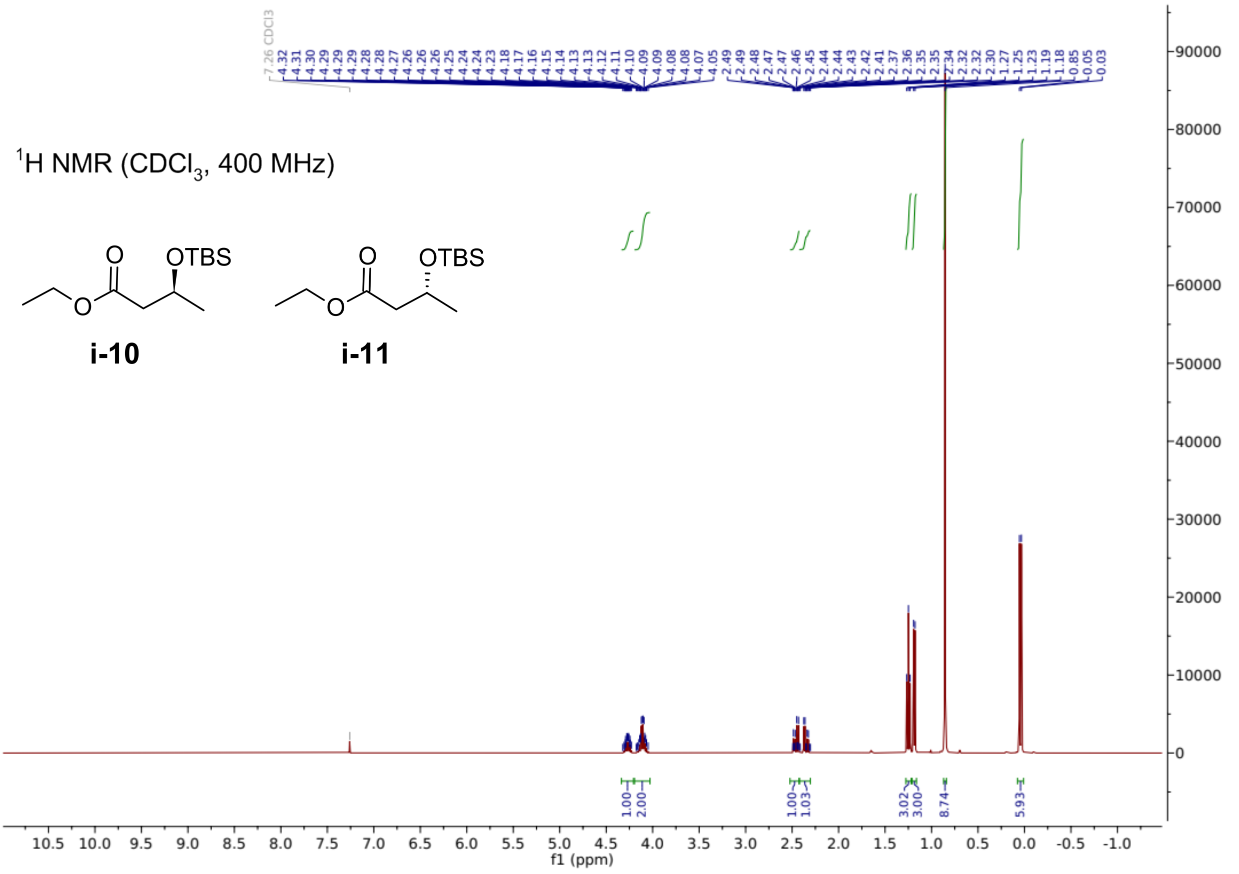

$^{13}C\{^{1}H\}$ NMR ($CDCl_3$, 101 MHz)

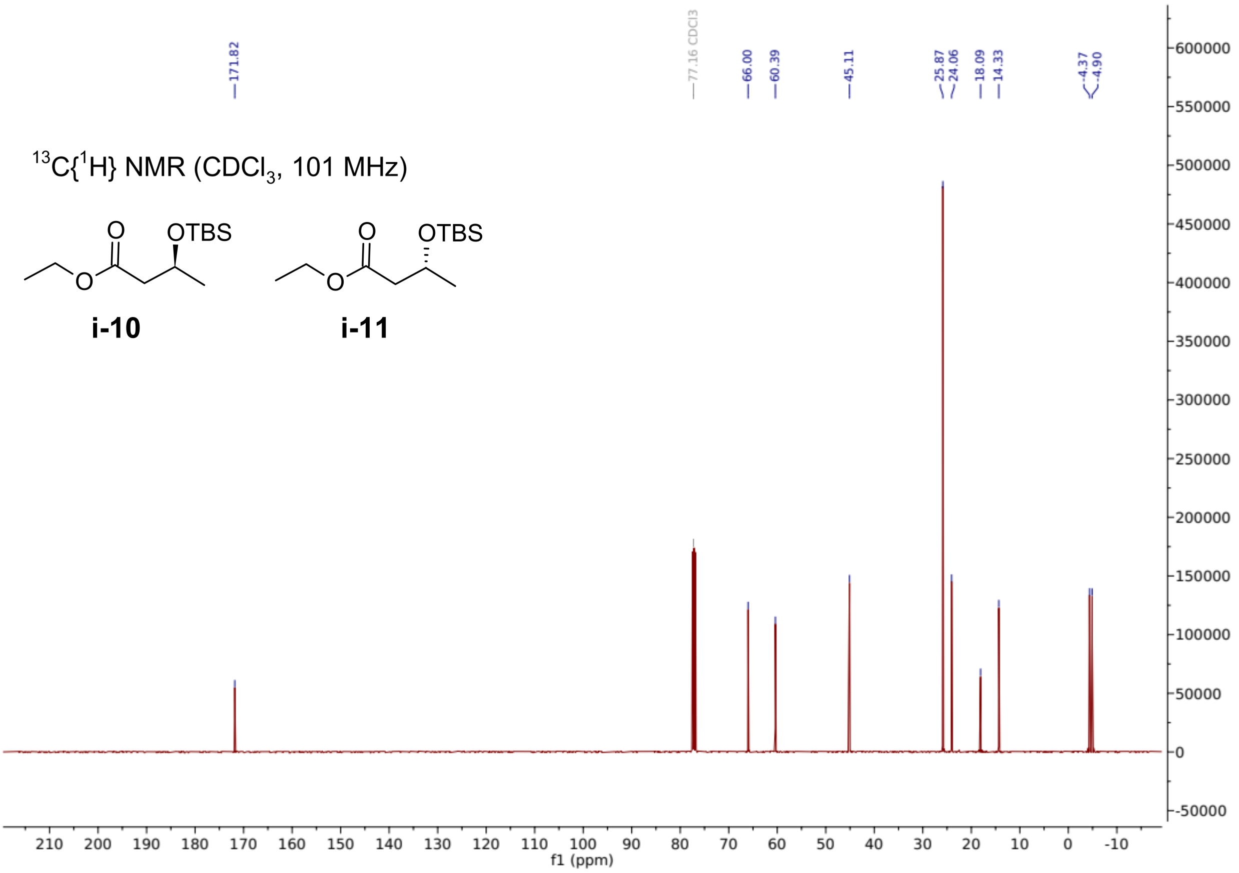

(3*S*)-3-((*tert*-butyl(dimethyl)silyl)oxy)butanal (10)
(3*R*)-3-((*tert*-butyl(dimethyl)silyl)oxy)butanal (11)

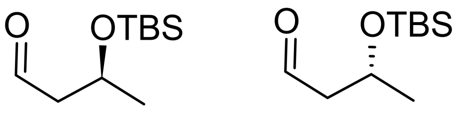

10 **11**

Compound **i-10** or **i-11** (4.6 g, 18.7 mmol, 1.0 eq.) is dissolved in anhydrous dichloromethane (93.5 ml) under nitrogen atmosphere and the solution is cooled to -78 °C. A solution of 1M DIBAL-H in hexane (19.6 ml, 19.6 mmol, 1.05 eq.) is added slowly dropwise with a syringe pump (4 ml/h) at -78 °C. After addition, the reaction mixture is allowed to stir for further 30 min at the same temperature. Then methanol (9.1 ml) is injected and again the solution is continued stirring for 15 min before the cooling bath is removed. At room temperature a saturated aqueous solution of Rochelle salt (35 ml) is added and the reaction mixture is stirred for additional 2 h at room temperature. After that the organic phase is separated and the aqueous layer is extracted with dichloromethane (3x65 ml). The combined organic layers are washed with water (100 ml), an aqueous saturated $NaHCO_3$ solution (65 ml) and brine (100 ml). The organic layer is dried over $MgSO_4$, filtered and concentrated under reduced pressure. After column chromatography (*n*-pentane/Et_2O 95:5) the aldehyde **10** or **11** (2.86 g, 14.16 mmol, 76 %) is obtained as a colorless oil.

General data: R_f = 0.54 (*n*-pentane/Et_2O, 9:1), **10**: $[\alpha]^{25}_D$ = +13.163 (c=1.018, $CHCl_3$),
11: $[\alpha]^{25}_D$ = -11.063 (c=1.6, DCM).

^{1}H NMR ($CDCl_3$, 600 MHz): δ 9.77 (dd, 1H, *J* = 2.9, 2.0 Hz), 4.37 - 4.29 (m, 1H), 2.52 (ddd, 1H, *J* = 15.7, 7.1, 2.8 Hz), 2.44 (ddd, 1H, *J* = 15.7, 4.9, 2.0 Hz), 1.21 (d, 3H, *J* = 6.2 Hz), 0.84 (s, 9H), 0.05 (s, 3H), 0.04 (s, 3H).

^{13}C{^{1}H} NMR ($CDCl_3$, 151 MHz): δ 202.3, 64.7, 53.1, 25.8, 24.3, 18.1, -4.3, -4.8.

Analytical data as described in the literature.[S3]

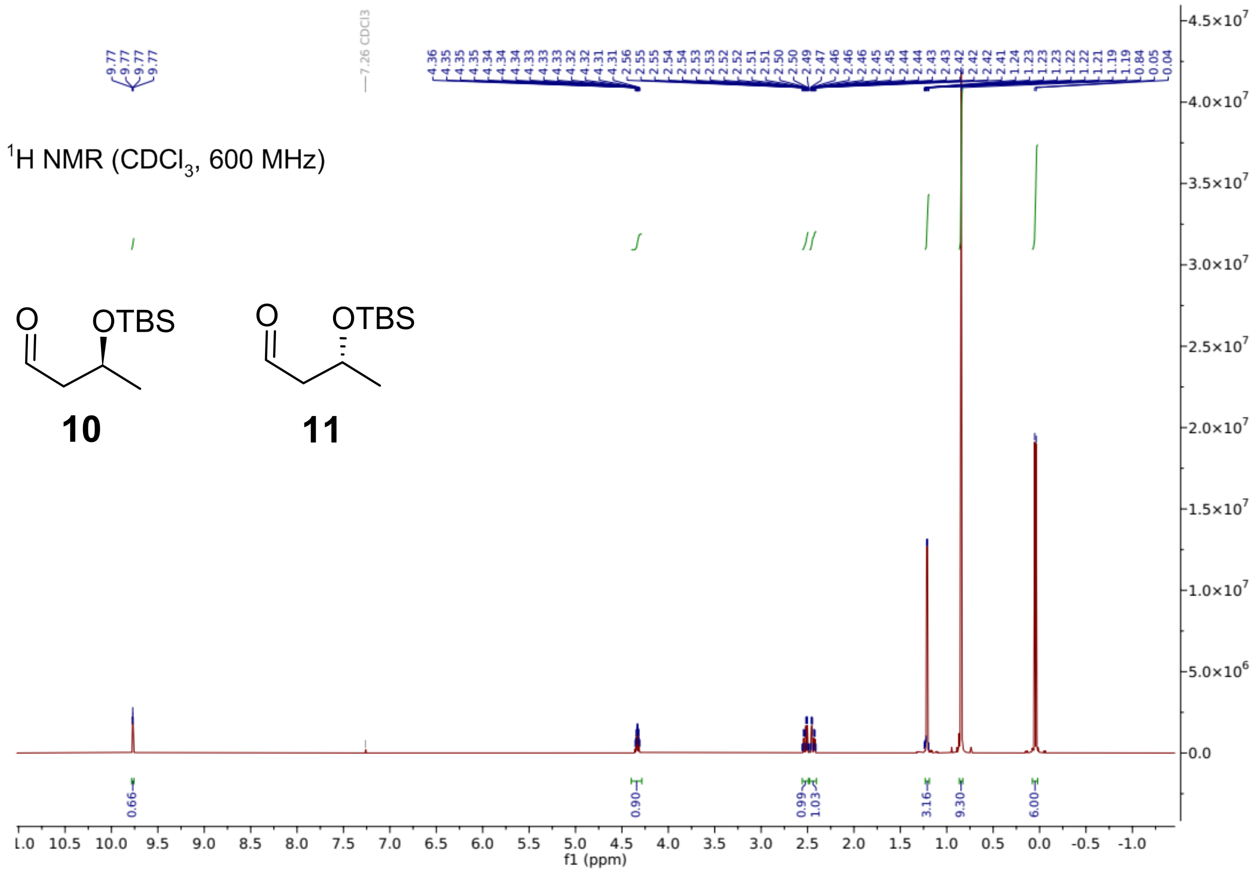

1H NMR (CDCl3, 600 MHz)
OTBS
OTBS
10
11

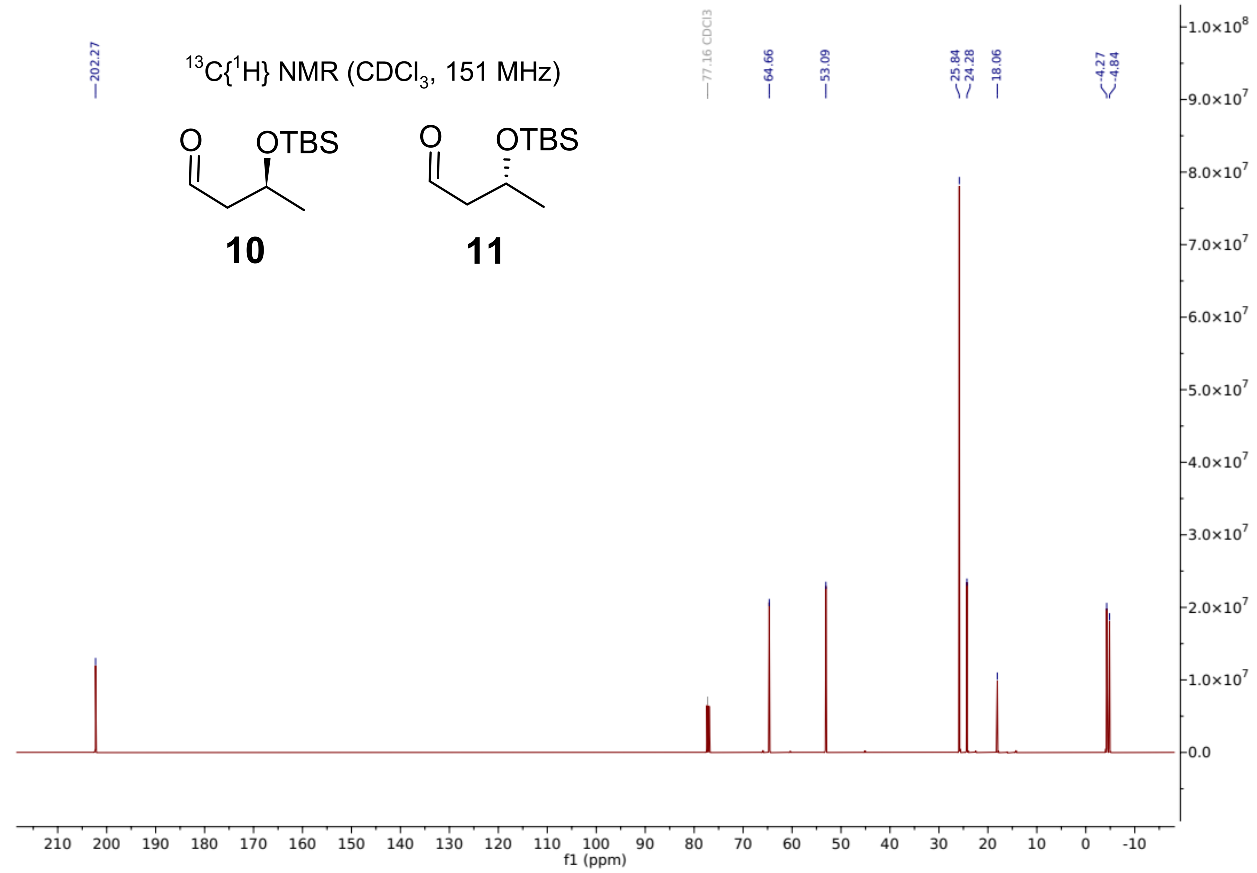

13C{1H} NMR (CDCl3, 151 MHz)
OTBS
OTBS
10
11

(2*R*,3*S*,5*S*)-1-((4*S*)-4-benzyl-2-thioxo-1,3-thiazolidin-3-yl)-5-((*tert*-butyl(dimethyl)silyl)oxy)-3-hydroxy-2-methylhexan-1-one (12)

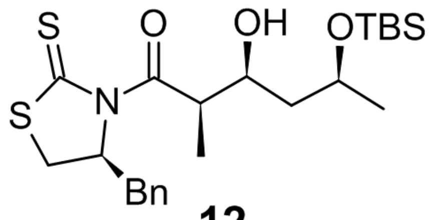

12

Under nitrogen atmosphere (*S*)-*N*-propionyl-thiazolidinethione **4** (for preparation see ref. S1) (2 g, 7.55 mmol, 1.0 eq.) is dissolved in anhydrous dichloromethane (60 ml) and cooled to 0 °C. Titanium tetrachloride (0.96 ml, 8.74 mmol, 1.16 eq.) is added slowly and the orange suspension is stirred for 5 min. Then DIPEA (1.49 ml, 8.74 mmol, 1.16 eq.) is introduced slowly which leads to a red/black solution. After stirring for 20 min at 0 °C the reaction mixture is cooled to -78 °C. (3*S*)-3-((*tert*-butyl(dimethyl)silyl)oxy)butanal **(10)** (1.82 g, 9.00 mmol, 1.2 eq.) is injected within two minutes and stirred for further 1 h at -78 °C. The reaction is quenched under vigorous stirring with saturated aqueous NH_4Cl solution (28 ml) at -78 °C. While warming to room temperature water (28 ml) is poured in. The organic layer is separated and the aqueous phase is extracted with dichloromethane (3x55 ml). The combined organic layers are dried over $MgSO_4$, filtered and the solvent is removed. The residue is purified by column chromatography (n-pentane/Et_2O, 9:1) to afford product **12** (2.87 g, 6.14 mmol, 81 %) as a yellow sticky oil.

General data: R_f = 0.27 (*n*-pentane/Et_2O, 3:1), $[\alpha]^{20}_D$ = +142.353 (c=0.51, $CHCl_3$), MW = 467.75 g/mol.

IR (neat): 3515 (w br), 3063 (w), 3028 (w), 2952 (m), 2928 (m), 2856 (m), 1681 (m), 1456 (m), 1361 (m), 1340 (m), 1252 (s), 1190 (m), 1157 (s), 1133 (s), 1061 (m), 1026 (m), 1001 (m), 962 (m), 893 (m), 834 (s), 775 (s), 744 (m), 701 (s) cm^{-1}.

^{1}H NMR ($CDCl_3$, 600 MHz): δ 7.36 - 7.27 (m, 5H), 5.43 (dddd, 1H, *J* = 11.1, 7.3, 3.9, 1.1 Hz), 4.67 (qd, 1H, *J* = 6.9, 3.6 Hz), 4.25 (ddd, 1H, *J* = 9.3, 3.6, 2.8 Hz), 4.08 (dqd, 1H, *J* = 12.1, 6.0, 4.6 Hz), 3.38 - 3.32 (m, 1H), 3.23 (dd, 1H, *J* = 13.3, 3.8 Hz), 3.05 - 2.98 (m, 1H), 2.87 (dd, 1H, *J* = 11.5, 1.2 Hz), 1.76 - 1.67 (m, 1H), 1.54 (ddd, 1H, *J* = 14.0, 4.6, 2.7 Hz), 1.23 (d, 3H, *J* = 7.0 Hz), 1.20 (d, 3H, *J* = 6.0 Hz), 0.88 (s, 9H), 0.10 (s, 3H), 0.10 (s, 3H).

^{13}C{^{1}H} NMR ($CDCl_3$, 151 MHz): δ 201.4, 177.3, 136.6, 129.6, 129.0, 127.3, 70.6, 69.08, 69.03, 43.8, 43.3, 37.1, 31.8, 26.0, 24.4, 18.1, 11.1, -3.9, -4.6.

HRMS (ESI): calculated for $C_{23}H_{37}NO_3S_2SiNa$ $[M+Na]^+$: 490.1882, found: 490.1868.

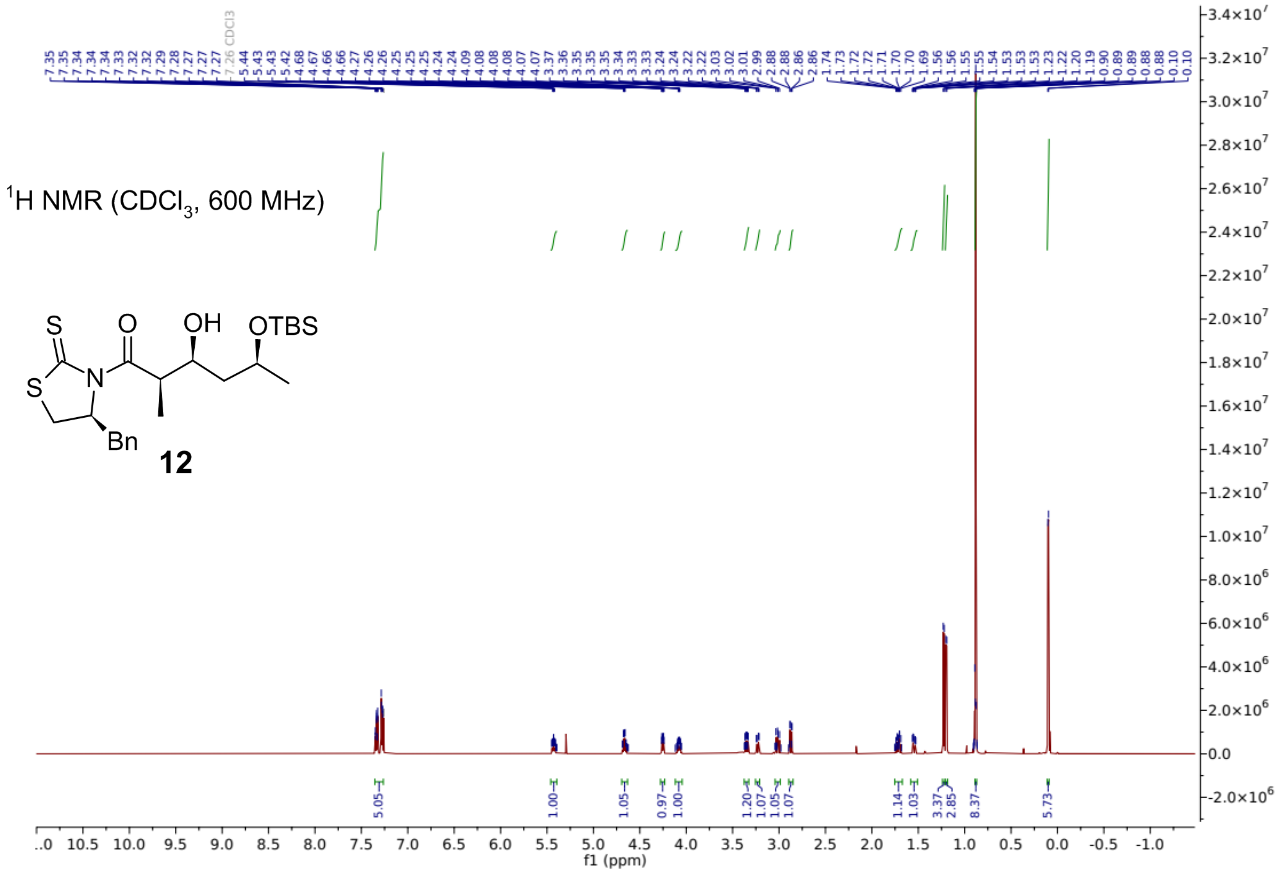

1H NMR (CDCl3, 600 MHz)
S
O
OH
OTBS
N
S
Bn
12
f1 (ppm)

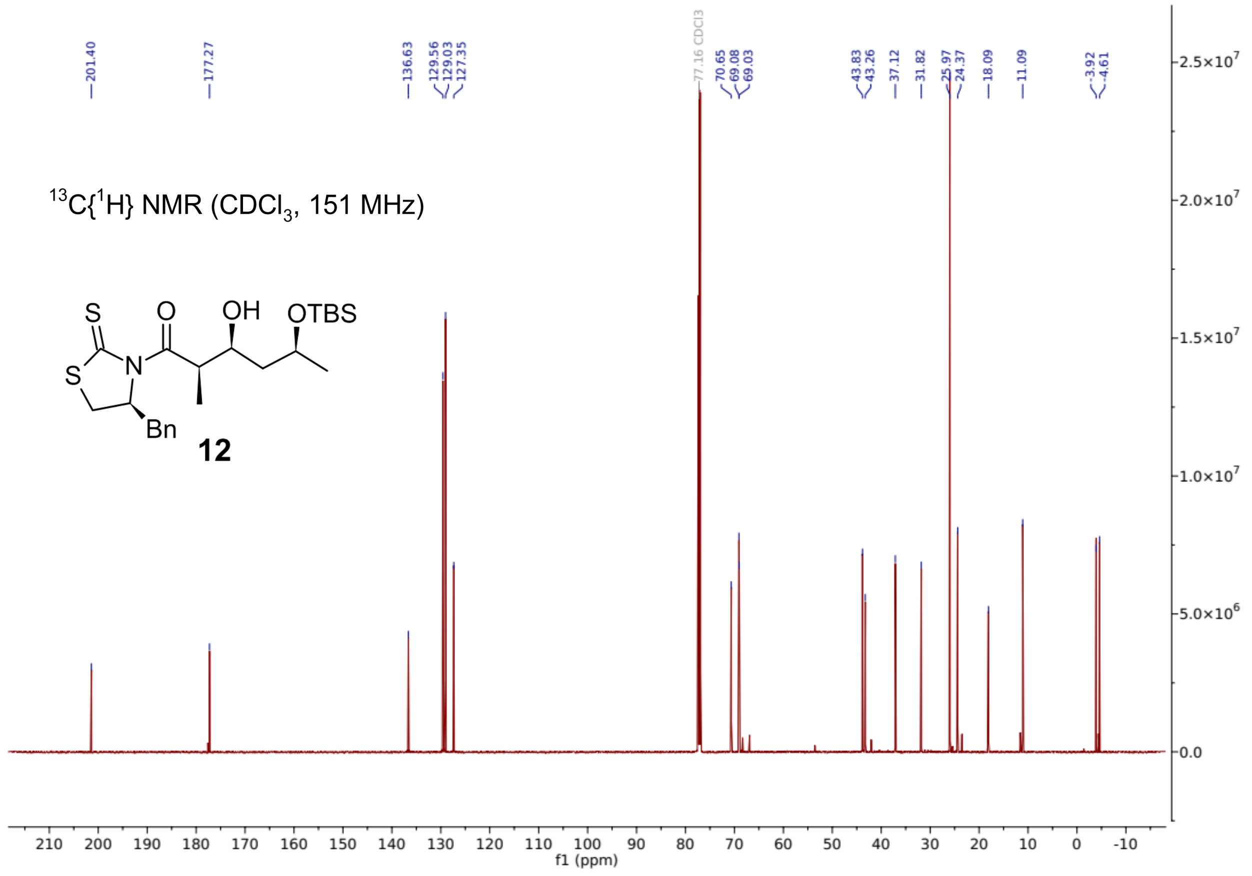

13C{1H} NMR (CDCl3, 151 MHz)
201.40
177.27
136.63
129.56
129.03
127.35
77.16 CDCl3
70.65
69.08
69.03
43.83
43.26
37.12
31.82
25.97
24.37
18.09
11.09
-3.92
-4.61
S
O
OH
OTBS
N
S
Bn
12
f1 (ppm)

(3*R*,4*S*,6*S*)-4-((*tert*-butyl(dimethyl)silyl)oxy)-3,6-dimethyloxan-2-one (14)

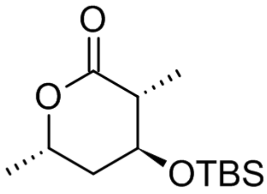

14

The aldol product **12** (2.87 g, 6.14 mmol, 1.0 eq.) is dissolved in dry dichloromethane (20.5 ml) and cooled to 0 °C. *Para*-toluenesulfonic acid monohydrate (642.4 mg, 3.38 mmol, 0.55 eq.) is added in portions and the solution is stirred for 1 h at 0 °C. After 23 h stirring at room temperature the colorless solution is again cooled to 0 °C and imidazole (1.25 g, 18.42 mmol, 3.0 eq.) and TBSCl (1.39 g, 9.21 mmol, 1.5 eq.) are introduced sequentially in portions. The reaction mixture is allowed to stir 10 min at 0 °C before it is warmed to room temperature and continues for another 25 h. Under intense stirring saturated aqueous $NaHCO_3$ solution (16 ml) is added to the solution, which is extracted with dichloromethane (4x20 ml). The organic layer is washed with water (16 ml), dried over $MgSO_4$, filtered and concentrated under reduced pressure. After column chromatography (*n*-pentane/Et_2O, 9:1) lactone **14** is provided (1.28 g, 4.95 mmol, 81 %) as a colorless oil, which crystallizes slowly at room temperature.

General data: R_f = 0.44 (*n*-pentane/Et_2O, 3:1), $[\alpha]^{20}_D$ = +13.021 (c=0.192, $CHCl_3$), m. p. = 38 °C, MW = 258.43 g/mol.

IR (neat): 2980 (w), 2953 (w), 2930 (w), 2887 (w), 2856 (w), 1746 (s), 1256 (s), 1204 (m), 1118 (m), 1086 (s), 1053 (s), 962 (m), 924 (s), 876 (s), 830 (s), 804 (m), 774 (s), 665 (w) cm^{-1}.

^{1}H NMR ($CDCl_3$, 400 MHz): δ 4.77 - 4.65 (m, 1H), 3.81 (td, 1H, *J* = 5.3, 3.3 Hz), 2.62 - 2.51 (m, 1H), 1.91 - 1.74 (m, 2H), 1.35 (d, 3H, *J* = 6.4 Hz), 1.25 (d, 3H, *J* = 7.1 Hz), 0.88 (s, 9H), 0.06 (d, 6H, *J* = 3.7 Hz).

^{13}C{^{1}H} NMR ($CDCl_3$, 101 MHz): δ 174.6, 71.8, 70.0, 43.3, 38.5, 25.8, 21.2, 18.0, 14.9, -4.5, -4.8.

HRMS (ESI): calculated for $C_{13}H_{27}O_3Si$ $[M+H]^+$: 259.1729, found: 259.1733.

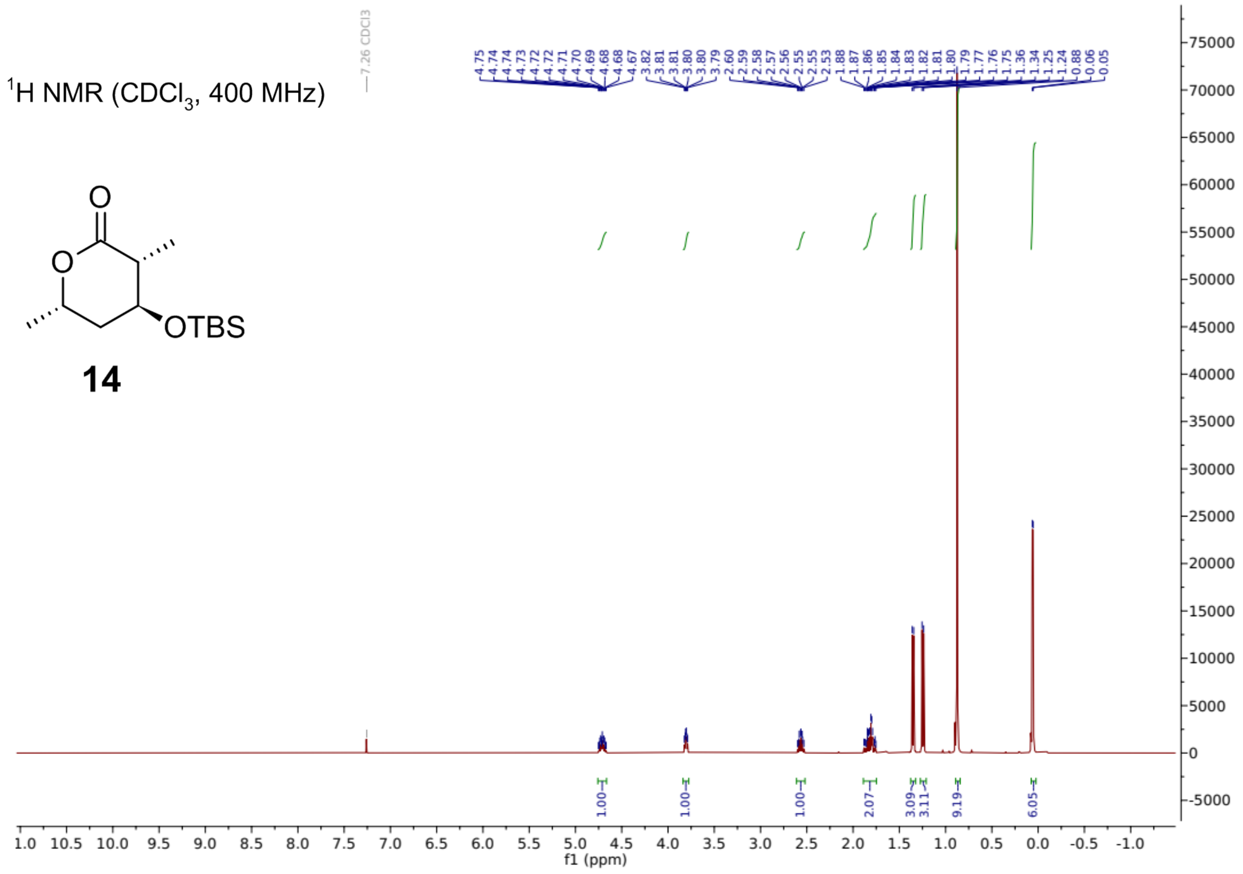
^{1}H NMR (CDCl$_3$, 400 MHz)
O
O
OTBS
14
7.26 CDCl3
4.75 4.74 4.74 4.73 4.72 4.72 4.71 4.70 4.69 4.68 4.68 4.67 3.82 3.81 3.81 3.80 3.80 3.79 2.60 2.59 2.58 2.57 2.56 2.55 2.55 2.53 1.88 1.87 1.86 1.85 1.84 1.83 1.82 1.81 1.80 1.79 1.77 1.76 1.75 1.36 1.34 1.25 1.24 0.88 0.06 0.05
1.00 1.00 1.00 2.07 3.09 3.11 9.19 6.05
f1 (ppm)

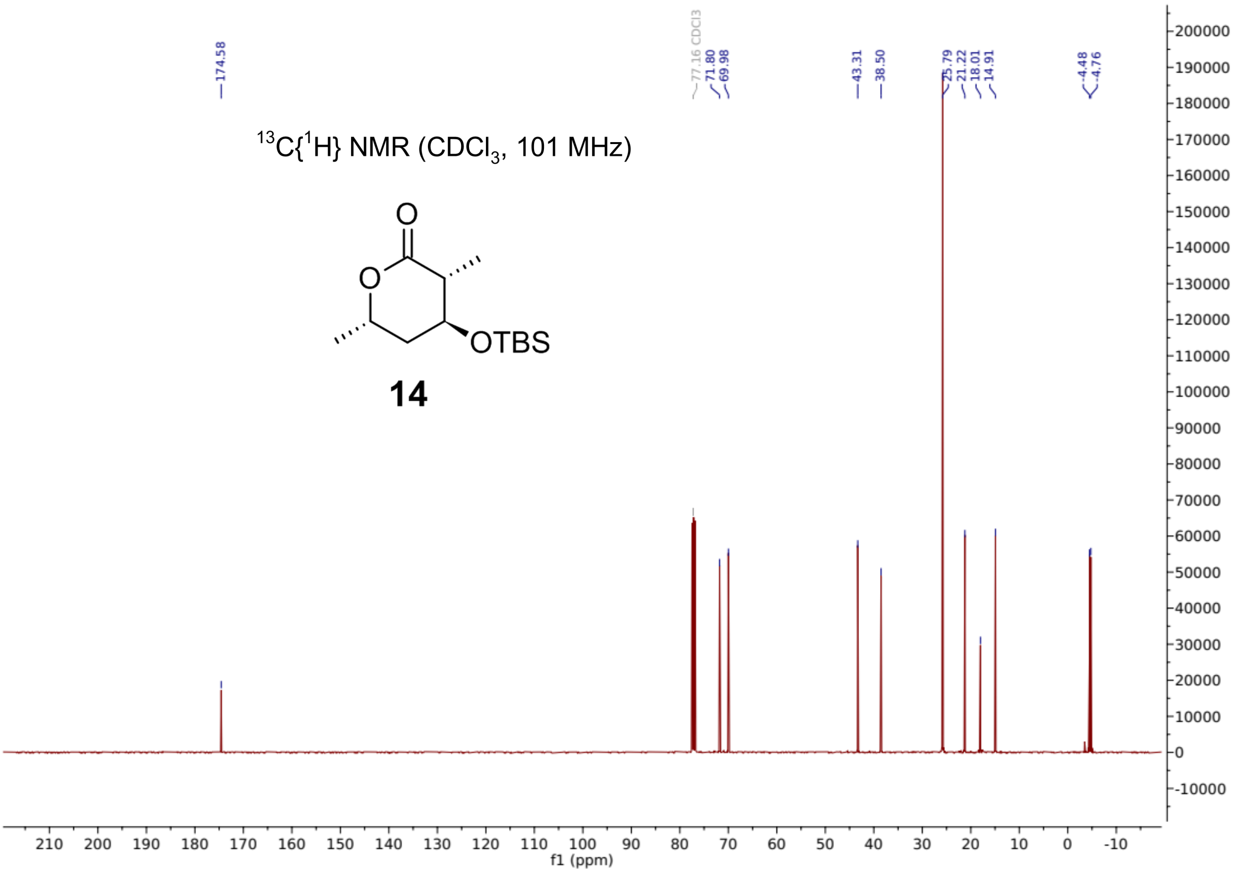
^{13}C{^{1}H} NMR (CDCl$_3$, 101 MHz)
O
O
OTBS
14
174.58
77.16 CDCl3
71.80
69.98
43.31
38.50
25.79
21.22
18.01
14.91
-4.48
-4.76
f1 (ppm)

(3*R*,4*S*,6*S*)-4-((*tert*-butyl(dimethyl)silyl)oxy)-3-((1*S*)-1-hydroxy-3-((4-methoxybenzyl)oxy)propyl)-3,6-dimethyloxan-2-one (17)

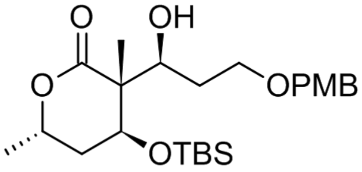

17

To a solution of lactone **14** (517 mg, 2.0 mmol, 1.0 eq.) in dry dichloromethane (9.2 ml) 1M Bu_2BOTf in dichloromethane (2.2 ml, 2.2 mmol, 1.1 eq.) is injected at -78 °C under nitrogen atmosphere. After stirring of the yellow solution for 20 min at -78 °C triethylamine (0.36 ml, 2.6 mmol, 1.3 eq.) is added slowly which leads to a colorless solution. The reaction mixture is allowed to stir 1 h at this temperature. Then a mixture of 3-((4-methoxybenzyl)oxy)propanal **(16)** (for preparation see ref. S1) (777 mg, 4.0 mmol, 2.0 eq.) with anhydrous dichloromethane (1.0 ml) is added slowly dropwise within 20 min. After stirring of the colorless solution for 3 h at -78 °C the cold reaction solution is quenched sequentially with 20 mM aqueous phosphate buffer (8 ml), methanol (4 ml) and 30 % H_2O_2 (2 ml) at -78 °C. The cooling bath is removed and vigorous stirring is continued for additional 1.5 h. Dichloromethane (10 ml) is added and the organic layer is separated. The aqueous layer is extracted with dichloromethane (3x20 ml) and the combined organic layers are washed once with an aqueous saturated Na_2SO_3 solution (13 ml). After drying over $MgSO_4$, filtration and removing of the solvent the residue is purified by column chromatography (*n*-pentane/Et_2O, 2:1) to afford product **17** (707 mg, 1.56 mmol, 78 %) as a colorless oil.

General data: R_f = 0.28 (*n*-pentane/Et_2O, 1:1), $[\alpha]^{20}_D$ = +12.037 (c=0.216, $CHCl_3$), MW = 452.66 g/mol.

IR (neat): 3467 (w br), 2953 (w), 2931 (w), 2885 (w), 2857 (w), 1717 (m), 1612 (w), 1513 (m), 1462 (m), 1247 (s), 1114 (m), 1067 (s), 1037 (s), 999 (m), 831 (s), 776 (s) cm^{-1}.

^{1}H NMR ($CDCl_3$, 400 MHz): δ 7.25 - 7.21 (m, 2H), 6.92 - 6.82 (m, 2H), 4.78 (ddp, 1H, *J* = 10.1, 6.4, 3.2 Hz), 4.44 (s, 2H), 4.12 (dd, 1H, *J* = 4.8, 2.5 Hz), 3.89 (dd, 1H, *J* = 10.5, 1.8 Hz), 3.80 (s, 3H), 3.69 (ddd, 1H, *J* = 9.2, 5.9, 4.6 Hz), 3.61 (ddd, 1H, *J* = 9.2, 7.9, 4.3 Hz), 3.23 (s, 1H), 2.00 - 1.77 (m, 3H), 1.72 (dddd, 1H, *J* = 14.2, 6.0, 4.3, 1.8 Hz), 1.36 (d, 3H, *J* = 6.4 Hz), 1.22 (s, 3H), 0.88 (s, 9H), 0.08 (s, 3H), 0.06 (s, 3H).

^{13}C{^{1}H} NMR ($CDCl_3$, 101 MHz): δ 175.8, 159.4, 130.0, 129.5, 114.0, 74.9, 73.2, 72.6, 69.1, 68.7, 55.4, 52.2, 36.8, 32.0, 25.9, 21.8, 18.1, 17.3, -4.4, -4.8.

HRMS (ESI): calculated for $C_{24}H_{41}O_6Si$ $[M+H]^+$: 453.2672, found: 453.2670.

^{1}H NMR ($CDCl_3$, 400 MHz)

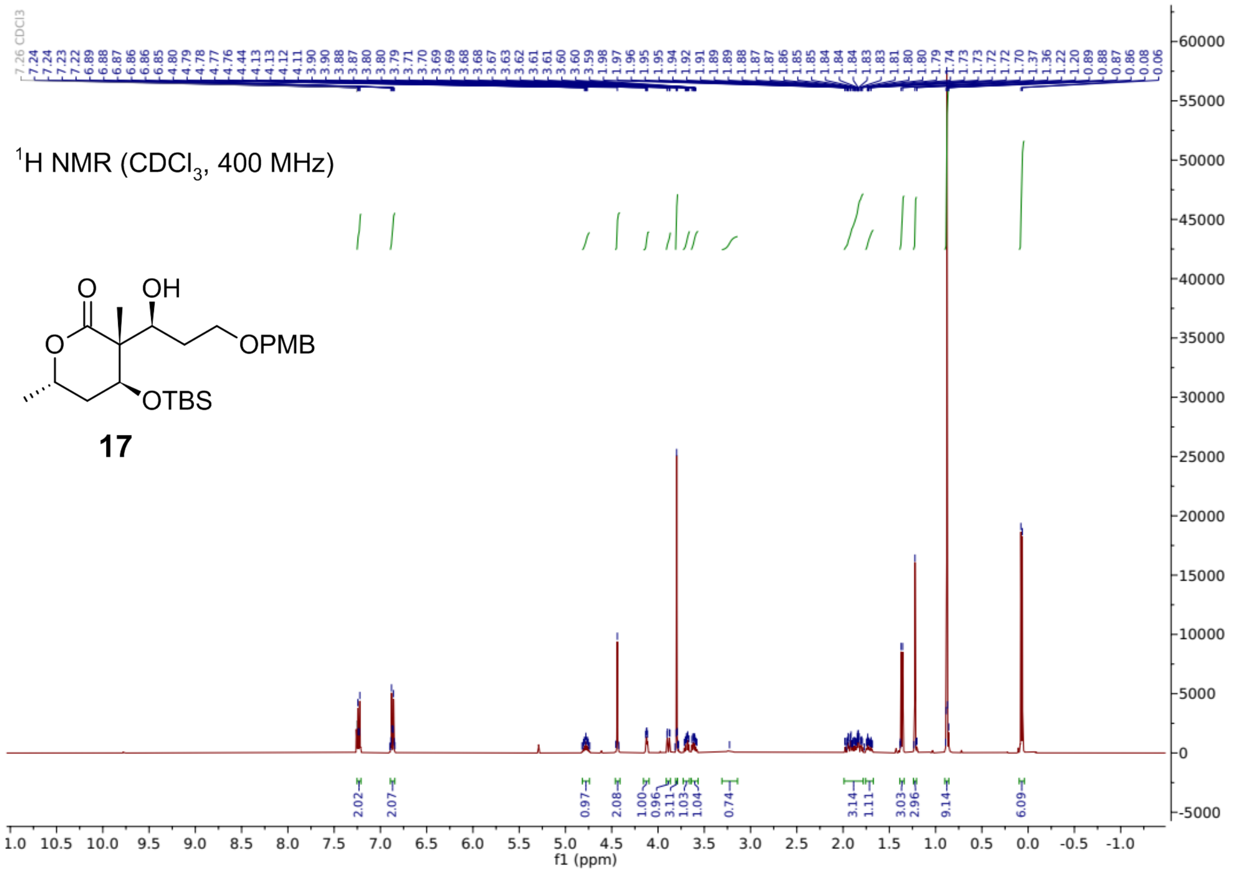

17

$^{13}C\{^{1}H\}$ NMR ($CDCl_3$, 101 MHz)

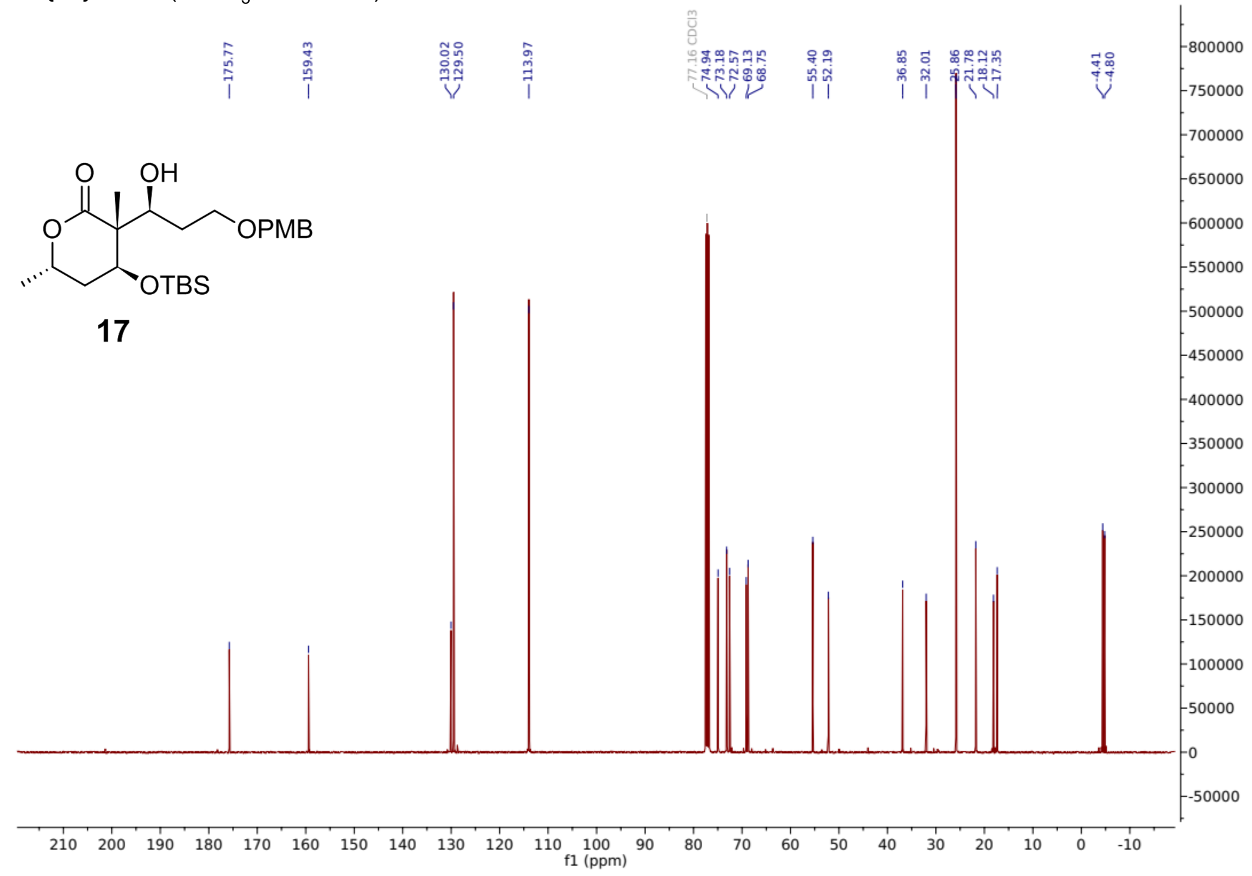

17

(3*R*,4*S*,6*S*)-4-hydroxy-3-((1*S*)-1-hydroxy-3-((4-methoxybenzyl)oxy)propyl)-3,6-dimethyloxan-2-one (20)

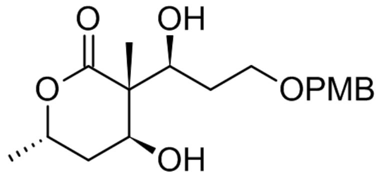

20

Compound **17** (63 mg, 0.14 mmol, 1.0 eq.) is dissolved in anhydrous THF (0.7 ml), cooled to 0 °C and 1M TBAF in THF (0.17 ml, 0.17 mmol, 1.2 eq.) is added. After 1 h stirring at 0 °C the colorless reaction solution is quenched with water (1.5 ml) and extracted with ethyl acetate (4x3 ml). The combined organic layers are dried over $MgSO_4$, filtered and concentrated under reduced pressure. After purification by column chromatography (freshly distilled diethyl ether, 100%) diol **20** is provided (14.7 mg, 0.04 mmol, 31 %) as a colorless crystalline solid.

In order to form crystals, which can be measured in X-ray, the crystalline solid is dissolved with some drops of freshly distilled anhydrous dichloromethane to create a high concentrated solution at room temperature. Then freshly distilled *n*-pentane is added dropwise until the solution becomes a suspension. At that point the *n*-pentane addition is stopped to let the floating crystals grow while the solvent is allowed to evaporate slowly at room temperature. After that short thin crystals like needles are obtained.

General data: R_f = 0.32 (Et_2O), $[\alpha]^{20}_D$ = -30.882 (c=0.204, $CHCl_3$), m. p. = 86 °C, MW = 338.4 g/mol.

IR (neat): 3481 (m br), 3408 (m br), 2981 (w), 2942 (m), 2911 (m), 2850 (w), 1710 (s), 1513 (m), 1369 (m), 1248 (s), 1191 (m), 1119 (s), 1101 (m), 1070 (s), 1053 (s), 1025 (s), 991 (m), 822 (s), 582 (m), 519 (m) cm^{-1}.

^{1}H NMR ($CDCl_3$, 400 MHz): δ 7.25 - 7.21 (m, 2H), 6.93 - 6.85 (m, 2H), 4.73 (pd, 1H, *J* = 6.6, 3.7 Hz), 4.52 - 4.41 (m, 3H), 4.29 (dd, 1H, *J* = 10.2, 1.4 Hz), 3.81 (s, 3H), 3.78 (dd, 1H, *J* = 8.5, 4.5 Hz), 3.71 (ddd, 1H, *J* = 10.7, 9.2, 2.8 Hz), 2.14 (ddd, 1H, *J* = 13.9, 10.2, 5.9 Hz), 2.03 - 1.85 (m, 2H), 1.71 (dddd, 1H, *J* = 14.1, 3.7, 2.8, 1.3 Hz), 1.46 (s, 3H), 1.39 (d, 3H, *J* = 6.6 Hz).

^{13}C{^{1}H} NMR ($CDCl_3$, 101 MHz): δ 175.1, 159.6, 129.6, 129.5, 114.1, 79.4, 73.5, 73.4, 70.3, 64.1, 55.4, 51.0, 33.1, 31.8, 22.5, 17.5.

HRMS (ESI): calculated for $C_{18}H_{27}O_6$ $[M+H]^+$: 339.1808, found: 339.1812.

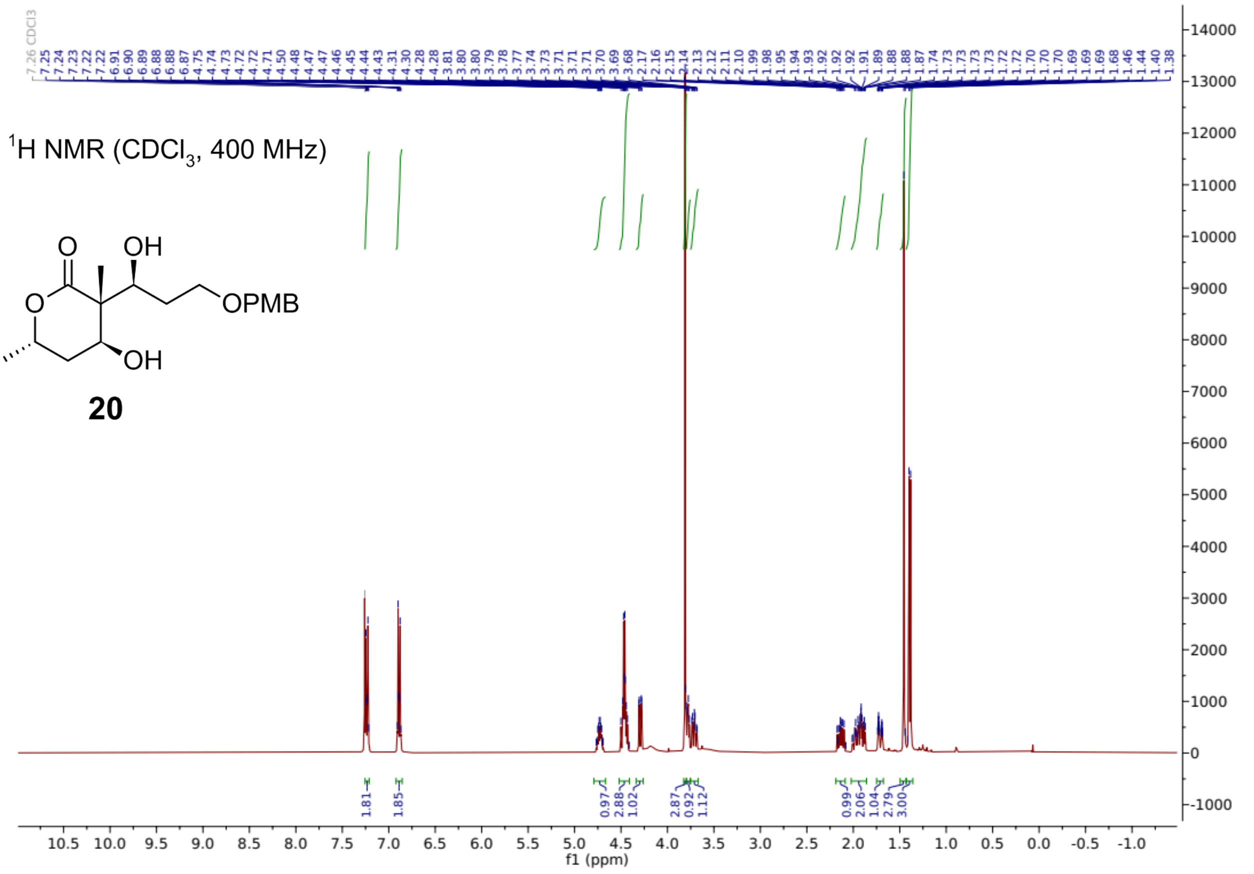
1H NMR (CDCl3, 400 MHz)
O
OH
O
OPMB
OH
20
f1 (ppm)

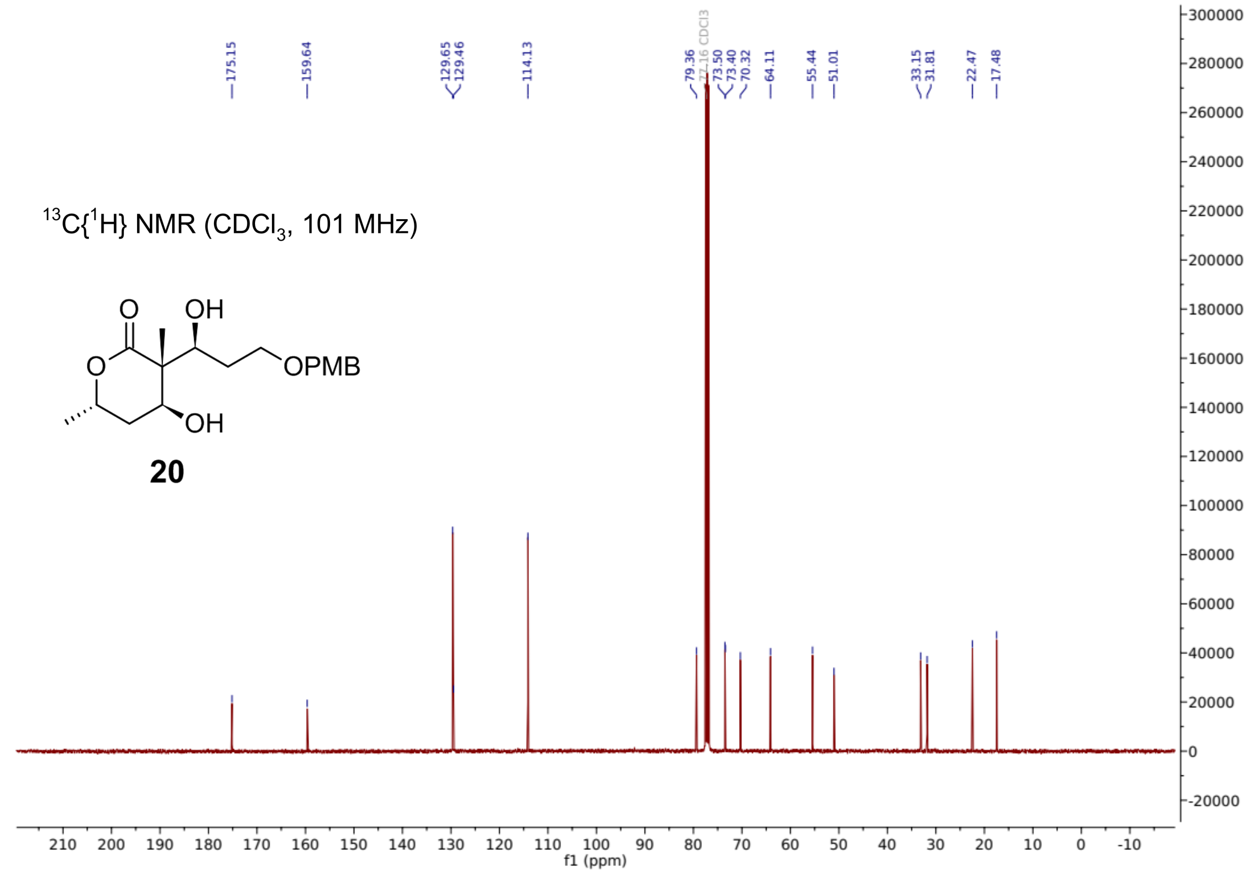
13C{1H} NMR (CDCl3, 101 MHz)
O
OH
O
OPMB
OH
20
f1 (ppm)

(3*R*,4*S*,6*S*)-4-((*tert*-butyl(dimethyl)silyl)oxy)-3-((1*S*)-3-((4-methoxybenzyl)oxy)-1-((triethylsilyl)oxy)propyl)-3,6-dimethyloxan-2-one (i-22)

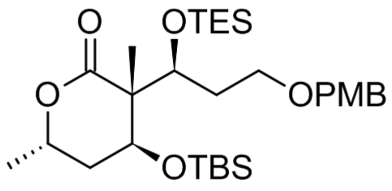

i-22

A solution of compound **17** (632 mg, 1.39 mmol, 1.0 eq.) in anhydrous dichloromethane (5.2 ml) is cooled to -78 °C under nitrogen atmosphere. DIPEA (0.48 ml, 2.79 mmol, 2.0 eq.) and TESOTf (0.45 ml, 1.95 mmol, 1.4 eq.) are added sequentially. After stirring for 2 h at -78 °C the reaction mixture is quenched with a saturated aqueous $NaHCO_3$ solution (2.5 ml) at -78 °C and diluted with dichloromethane (10 ml). The mixture is allowed to warm to ambient temperature, the organic phase is separated and the aqueous layer is extracted with dichloromethane (3x15 ml). The combined organic layers are dried over $MgSO_4$, filtered and concentrated under reduced pressure. After column chromatography (*n*-pentane/Et_2O, 9:1) compound **i-22** (696.3 mg, 1.23 mmol, 88.4 %) is obtained as a colorless oil.

General data: R_f = 0.45 (*n*-pentane/Et_2O, 3:1), $[\alpha]^{20}_D$ = -5.585 (c=0.376, $CHCl_3$),
MW = 566.92 g/mol.

IR (neat): 2953 (m), 2934 (m), 2876 (m), 2857 (m), 1745 (m), 1513 (m), 1461 (m), 1248 (s), 1183 (m), 1096 (s), 1071 (s), 998 (s), 830 (s), 776 (s), 739 (s) cm^{-1}.

^{1}H NMR (C_6D_6, 600 MHz): δ 7.28 - 7.22 (m, 2H), 6.84 - 6.79 (m, 2H), 4.69 (dqd, 1H, *J* = 12.8, 6.4, 3.5 Hz), 4.42 (dd, 1H, *J* = 9.0, 2.3 Hz), 4.38 - 4.30 (m, 2H), 3.99 (dd, 1H, *J* = 3.8, 2.0 Hz), 3.55 (td, 1H, *J* = 9.3, 4.8 Hz), 3.44 (ddd, 1H, *J* = 9.1, 6.1, 3.9 Hz), 3.31 (s, 3H), 1.84 - 1.79 (m, 1H), 1.80 - 1.73 (m, 1H), 1.58 (dddd, 1H, *J* = 13.9, 8.9, 4.8, 3.9 Hz), 1.47 - 1.43 (m, 4H), 1.13 (d, 3H, *J* = 6.4 Hz), 1.08 (t, 9H, *J* = 8.0 Hz), 0.87 (s, 9H), 0.83 - 0.70 (m, 6H), 0.01 (s, 3H), -0.09 (s, 3H).

^{13}C{^{1}H} NMR (C_6D_6, 151 MHz): δ 173.9, 159.9, 130.9, 129.8, 128.3, 114.1, 73.8, 73.0, 72.0, 70.3, 66.9, 54.8, 53.7, 36.8, 34.5, 25.9, 21.8, 18.2, 17.5, 7.5, 5.8, -4.4, -4.8.

HRMS (ESI): calculated for $C_{30}H_{58}NO_6Si_2$ $[M+NH_4]^+$: 584.3803, found: 584.3810.

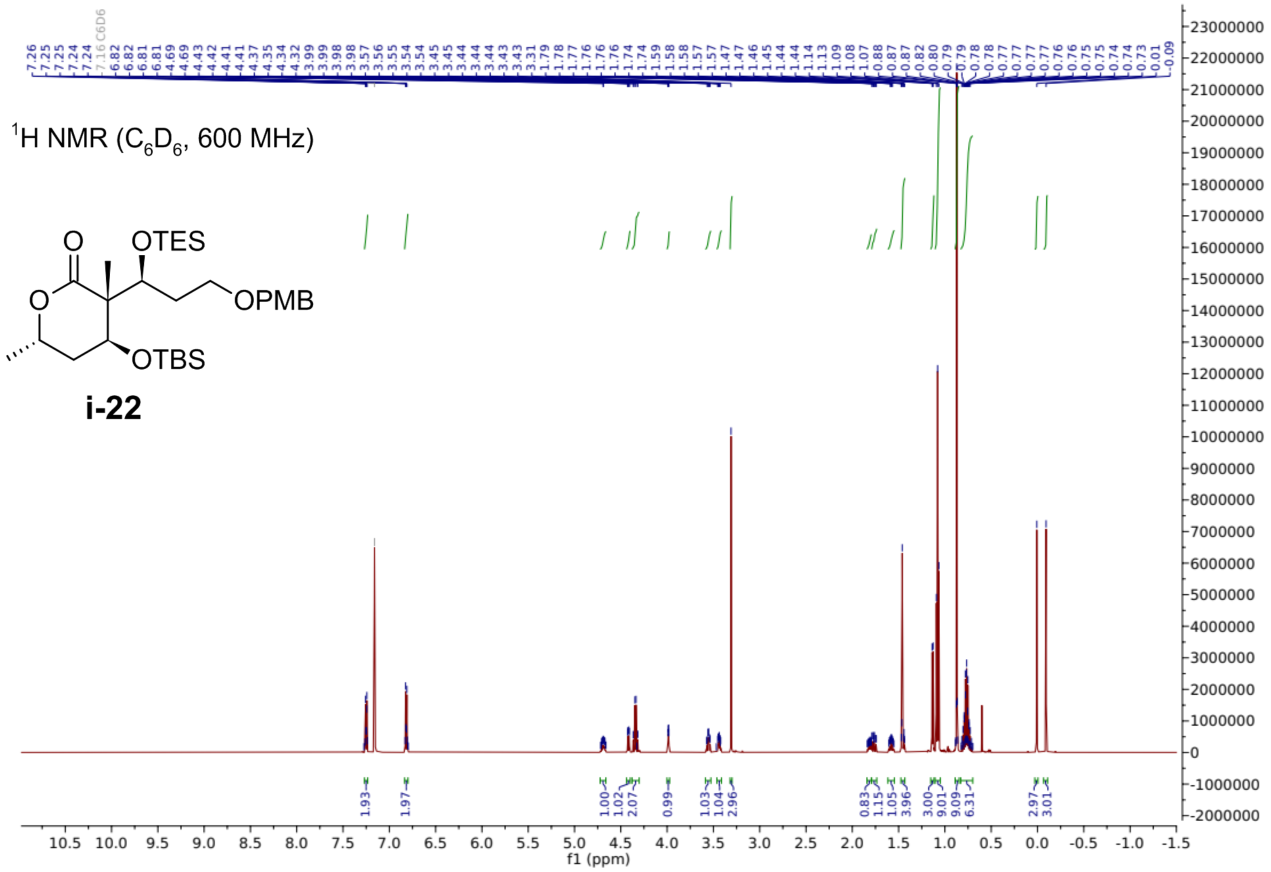

^{1}H NMR (C_6D_6, 600 MHz)
OTES
OPMB
OTBS
f1 (ppm)

i-22

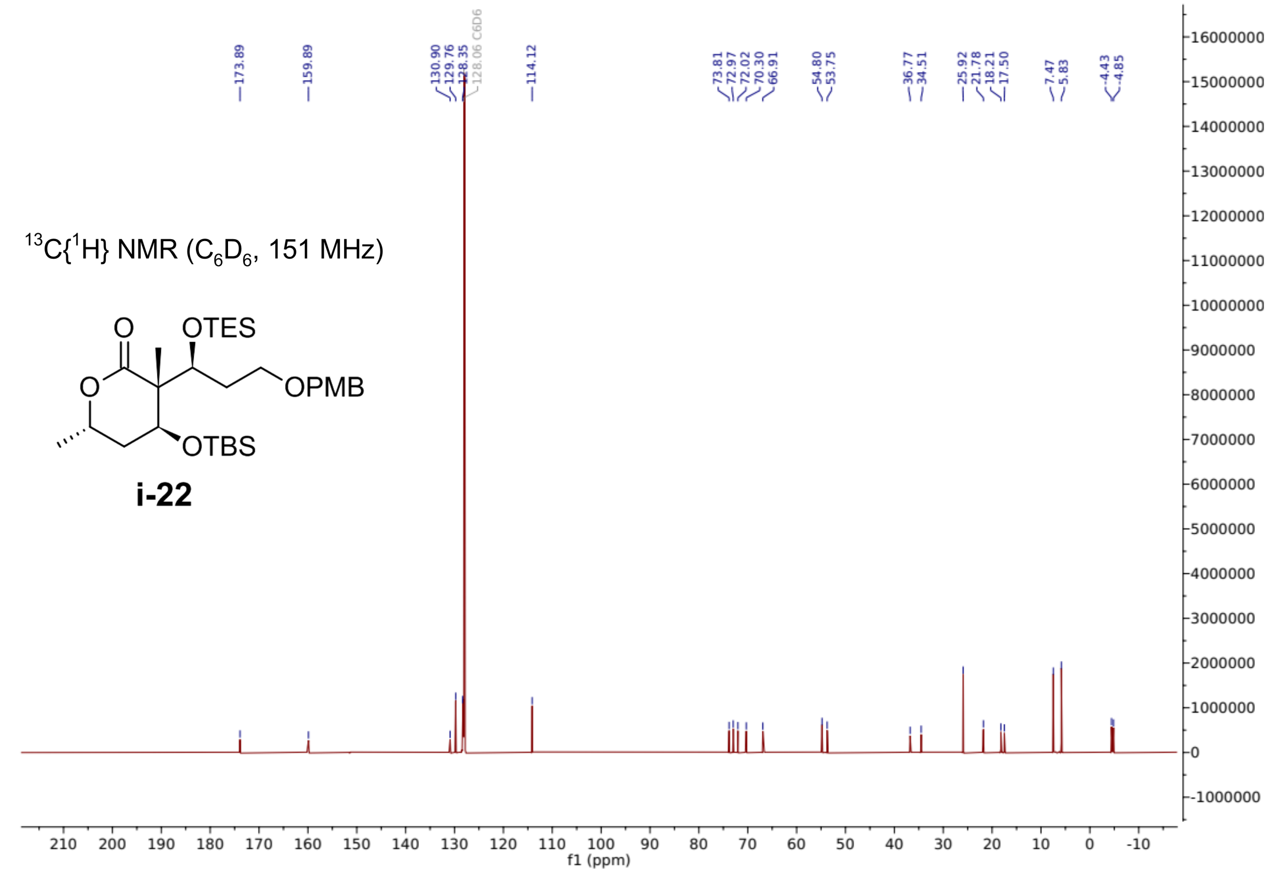

^{13}C{^{1}H} NMR (C_6D_6, 151 MHz)
OTES
OPMB
OTBS
f1 (ppm)

i-22

Methyl (2*R*,3*S*,5*S*)-3-((*tert*-butyl(dimethyl)silyl)oxy)-5-hydroxy-2-((1*S*)-3-((4-methoxybenzyl)oxy)-1-((triethylsilyl)oxy)propyl)-2-methylhexanoate (22)

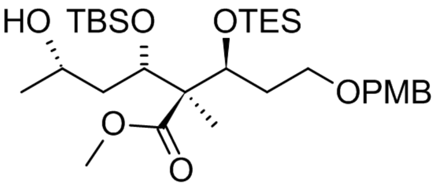

22

Note: The KOH solution in anhydrous methanol is prepared with 86.2 % potassium hydroxide pellets.

In a 25 ml pointed flask lactone **i-22** (293 mg, 0.517 mmol, 1.0 eq.) is dissolved in anhydrous THF (0.3 ml) and anhydrous methanol (0.1 ml). A 1.01 M KOH solution in anhydrous methanol (0.67 ml, 0.6767 mmol, 1.31 eq.) is added slowly at 0 °C and the reaction solution is allowed to stir for further 4.5 h in the ice bath. After stirring for 26 h at room temperature the clear bright yellow solution is monitored by TLC (*n*-pentane/Et_2O, 1:1). Another portion of the same 1.01 M KOH solution (0.15 ml, 0.1515 mmol, 0.29 eq.) is injected slowly at room temperature and again the reaction solution is stirred for additional 22 h. A solution of camphorsulfonic acid (191.9 mg, 0.826 mmol, 1.6 eq.) in anhydrous methanol (2.5 ml) is added slowly dropwise at 0 °C. After stirring for 20 min at 0 °C diethyl ether (7 ml) is added. Then the reaction mixture is allowed to stir further 10 min at 0 °C before it is warmed to room temperature. The reaction solution becomes a white suspension which must be stirred more vigorous to keep it homogeneous. $TMSCH_2N_2$ solution in hexane is injected in portions until TLC shows full conversion of the carboxylic acid intermediate (R_f: 0.6 (*n*-pentane/Et_2O, 1:1)). Excess of $TMSCH_2N_2$ represented by a yellow suspension must be destroyed by slowly dropwise addition of acetic acid glacial at 0 °C. To the resulting white suspension triethylamine (1.1 ml) is added immediately. After warm up the suspension, dichloromethane (10 ml) and a saturated aqueous $NaHCO_3$ solution (2 ml) are poured in a separating funnel. After shaking water (3 ml) is added and the organic layer is separated. The aqueous layer is extracted with dichloromethane (3x10 ml) and the combined organic layers are dried over $MgSO_4$, filtered and concentrated under reduced pressure. The residue is purified by column chromatography (*n*-pentane/ethyl acetate, 95:5 -> 9:1) to afford product **22** (210 mg, 0.35 mmol, 68 %) as a colorless oil.

CAUTION! *$TMSCH_2N_2$ (trimethylsilyldiazomethane) is fatal, may cause cancer and causes damage to organs. Solutions in hexane (0.5M to 2M) were purchased. The solutions were handled using needle and syringe techniques under a well ventilated fume hood.*

General data: R_f = 0.45 (*n*-pentane/Et_2O, 2:1), $[\alpha]^{20}_D$ = -22.642 (c=0.318, DCM), MW = 598.96 g/mol.

IR (neat): 2953 (m), 2932 (m), 2876 (m), 2856 (m), 1723 (m), 1513 (m), 1462 (m), 1245 (s), 1073 (s), 1037 (m), 1004 (s), 835 (s), 774 (s), 733 (s) cm^{-1}.

^{1}H NMR (C_6D_6, 400 MHz): δ 7.30 - 7.22 (m, 2H), 6.88 - 6.80 (m, 2H), 4.75 (dd, 1H, *J* = 9.8, 1.1 Hz), 4.40 - 4.27 (m, 2H), 4.07 - 3.99 (m, 1H), 3.79 (t, 1H, *J* = 5.6 Hz), 3.60 (ddd, 1H, *J* = 10.1, 9.0, 4.0 Hz), 3.48 (s, 3H), 3.44 (ddd, 1H, *J* = 9.0, 5.6, 3.7 Hz), 3.32 (s, 3H), 2.16 (dt, 1H, *J* = 14.8, 5.1 Hz), 2.10 - 1.98 (m, 2H), 1.79 (dddd, 1H, *J* = 11.3, 6.7, 5.8, 3.0 Hz), 1.54 (ddt, 1H, *J* = 13.9, 9.8, 3.8 Hz), 1.47 (s, 3H), 1.11 (d, 3H, *J* = 6.1 Hz), 1.06 (t, 9H, *J* = 8.0 Hz), 0.99 (s, 9H), 0.84 - 0.60 (m, 6H), 0.14 (s, 3H), 0.12 (s, 3H).

^{13}C{^{1}H} NMR (C_6D_6, 101 MHz): δ 174.9, 159.9, 130.8, 129.7, 114.2, 76.3, 73.2, 72.0, 67.0, 64.6, 58.7, 54.8, 51.1, 43.6, 33.9, 26.2, 24.3, 18.5, 15.6, 7.4, 6.0, -3.3, -4.1.

HRMS (ESI): calculated for $C_{31}H_{59}O_7Si_2$ $[M+H]^+$: 599.3799, found: 599.3789.

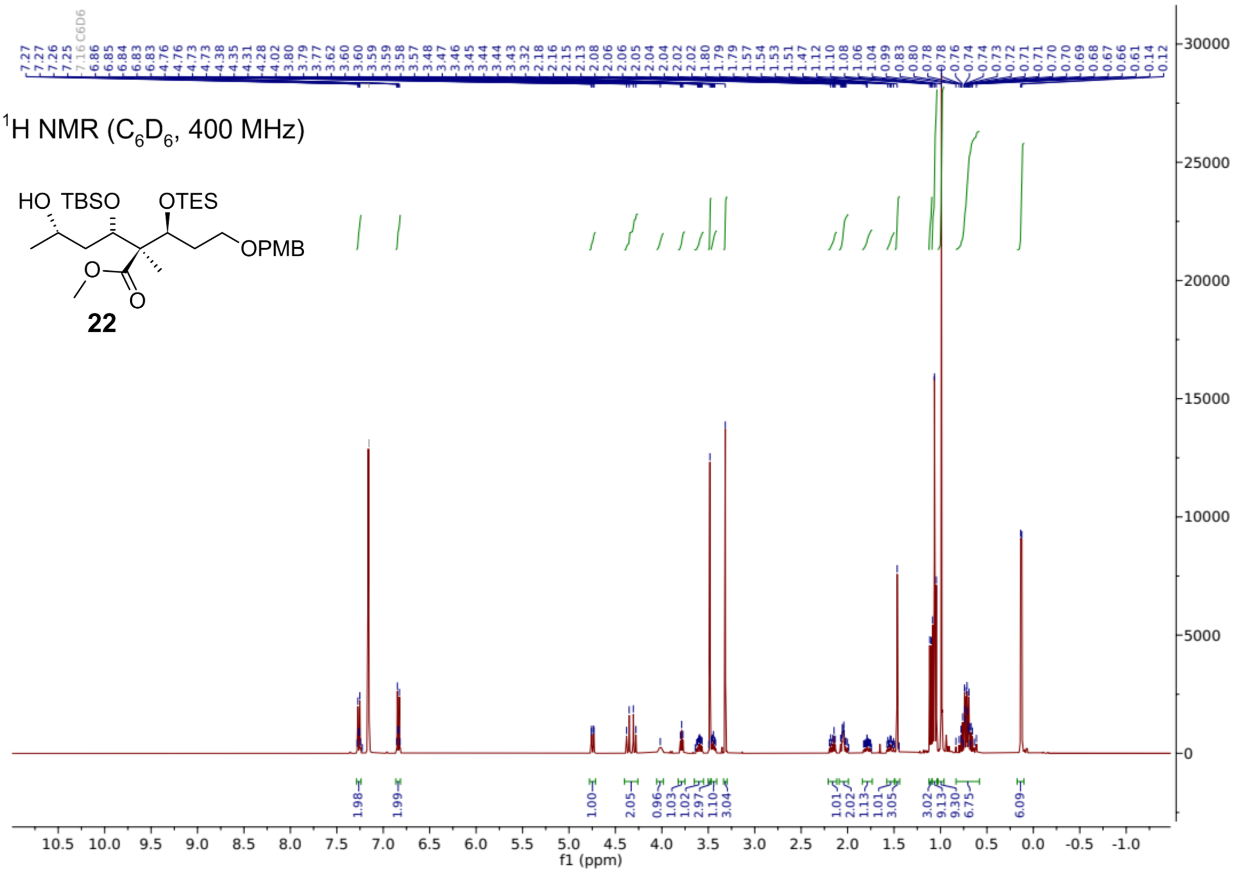

^{1}H NMR (C_6D_6, 400 MHz)
HO TBSO OTES
OPMB
O
O
O
22
f1 (ppm)

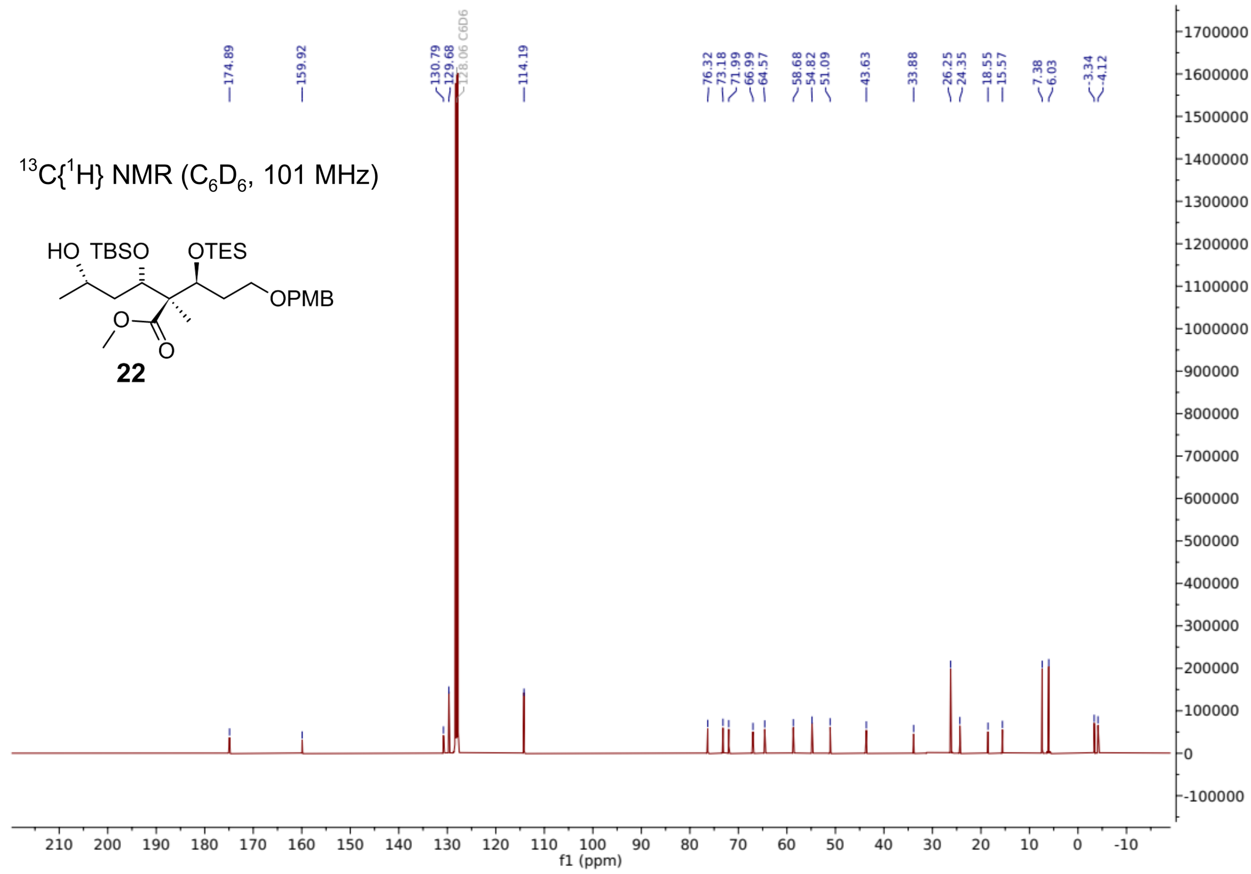

^{13}C{^{1}H} NMR (C_6D_6, 101 MHz)
HO TBSO OTES
OPMB
O
O
O
22
f1 (ppm)

(2*R*,3*S*,5*R*)-1-((4*S*)-4-benzyl-2-thioxo-1,3-thiazolidin-3-yl)-5-((*tert*-butyl(dimethyl)silyl)oxy)-3-hydroxy-2-methylhexan-1-one (13)

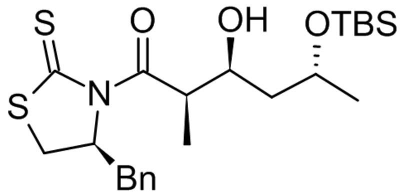

13

Under nitrogen atmosphere (*S*)-*N*-propionyl-thiazolidinethione **4** (for preparation see ref. S1) (2 g, 7.55 mmol, 1.0 eq.) is dissolved in anhydrous dichloromethane (60 ml) and cooled to 0 °C. Titanium tetrachloride (0.96 ml, 8.74 mmol, 1.16 eq.) is added slowly and the orange suspension is stirred for 5 min. Then DIPEA (1.49 ml, 8.74 mmol, 1.16 eq.) is introduced slowly which leads to a red/black solution. After stirring for 20 min at 0 °C the reaction mixture is cooled to -78 °C. (3*R*)-3-((*tert*-butyl(dimethyl)silyl)oxy)butanal **(11)** (1.8 g, 8.91 mmol, 1.18 eq.) is injected within two minutes and stirred for further 1 h at -78 °C. The reaction is quenched under vigorous stirring with saturated aqueous NH_4Cl solution (28 ml) at -78 °C. While warming to room temperature water (28 ml) is poured in. The organic layer is separated and the aqueous phase is extracted with dichloromethane (3x55 ml). The combined organic layers are dried over $MgSO_4$, filtered and the solvent is removed. The residue is purified by column chromatography (n-pentane/Et_2O, 9:1) to afford product **13** (2.8 g, 6 mmol, 79 %) as a yellow sticky oil, which crystallizes slowly at room temperature.

General data: R_f = 0.26 (*n*-pentane/Et_2O, 3:1), $[\alpha]^{20}_D$ = +140.076 (c=0.262, $CHCl_3$), m. p. = 85 °C, MW = 467.75 g/mol.

IR (neat): 3591 (m), 2977 (w), 2951 (m), 2927 (m), 2886 (w), 2850 (m), 1679 (s), 1251 (s), 1158 (s), 1132 (s), 1059 (s), 1039 (s), 1009 (m), 984 (s), 832 (s), 805 (m), 774 (s), 750 (s), 706 (s) cm^{-1}.

^{1}H NMR ($CDCl_3$, 600 MHz): δ 7.35 - 7.27 (m, 5H), 5.39 (dddd, 1H, *J* = 11.3, 7.3, 3.9, 1.0 Hz), 4.65 (qd, 1H, *J* = 7.0, 3.7 Hz), 4.39 (ddt, 1H, *J* = 10.3, 4.0, 2.1 Hz), 4.18 (pd, 1H, *J* = 6.3, 3.4 Hz), 3.43 (dd, 1H, *J* = 2.2, 0.6 Hz), 3.35 (ddd, 1H, *J* = 11.5, 7.2, 1.0 Hz), 3.23 (dd, 1H, *J* = 13.2, 3.9 Hz), 3.04 (dd, 1H, *J* = 13.2, 10.5 Hz), 2.87 (dd, 1H, *J* = 11.5, 1.0 Hz), 1.73 (ddd, 1H, *J* = 13.9, 10.3, 3.5 Hz), 1.51 - 1.44 (m, 1H), 1.23 (d, 3H, 3H, *J* = 6.7 Hz), 0.89 (s, 9H), 0.10 (s, 3H), 0.08 (s, 3H).

^{13}C{^{1}H} NMR ($CDCl_3$, 151 MHz): δ 201.5, 177.6, 136.6, 129.6, 129.0, 127.3, 69.1, 68.3, 66.9, 43.7, 42.0, 37.1, 31.8, 26.0, 23.5, 18.1, 11.6, -4.3, -4.8.

HRMS (ESI): calculated for $C_{23}H_{37}NO_3S_2SiNa$ $[M+Na]^+$: 490.1882, found: 490.1868.

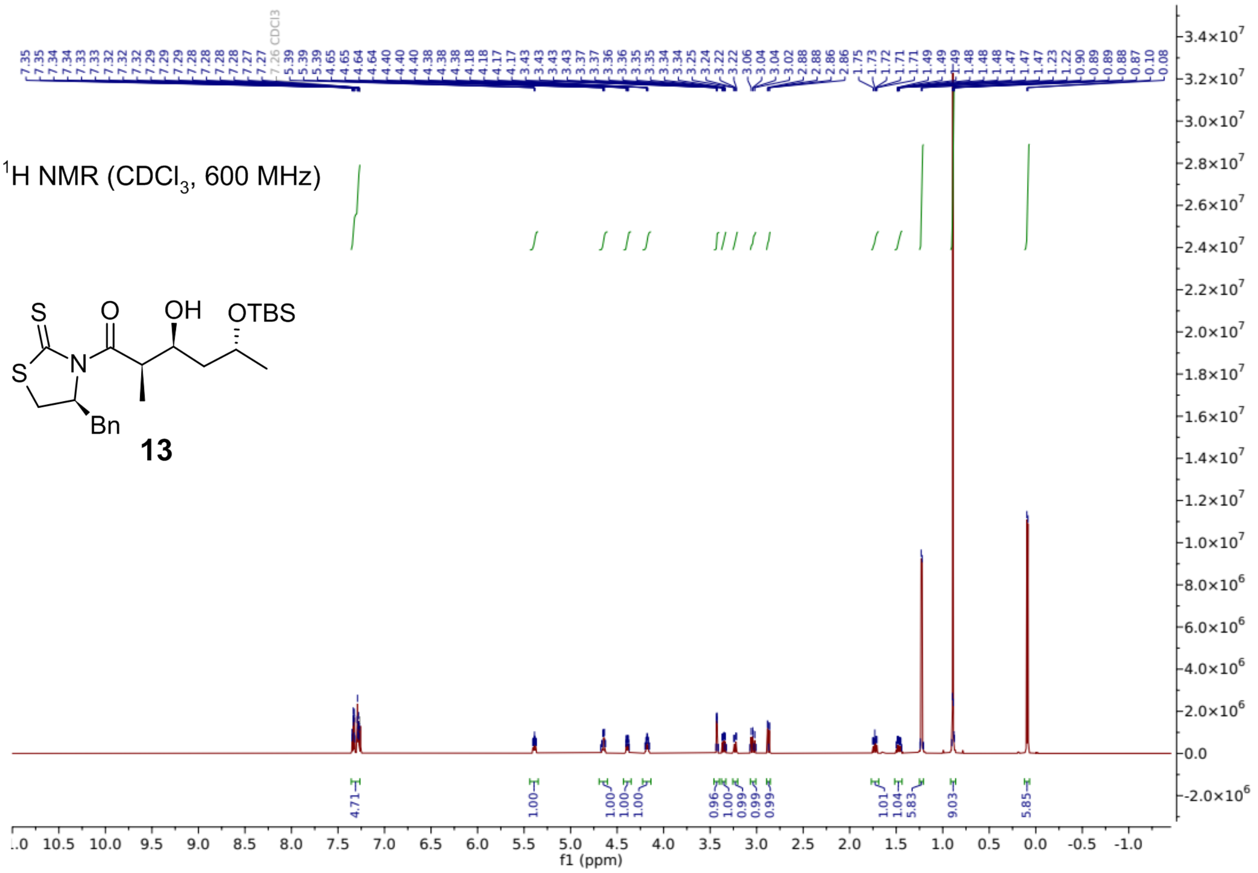

1H NMR (CDCl3, 600 MHz)
S
O
OH
OTBS
N
S
Bn
13
f1 (ppm)

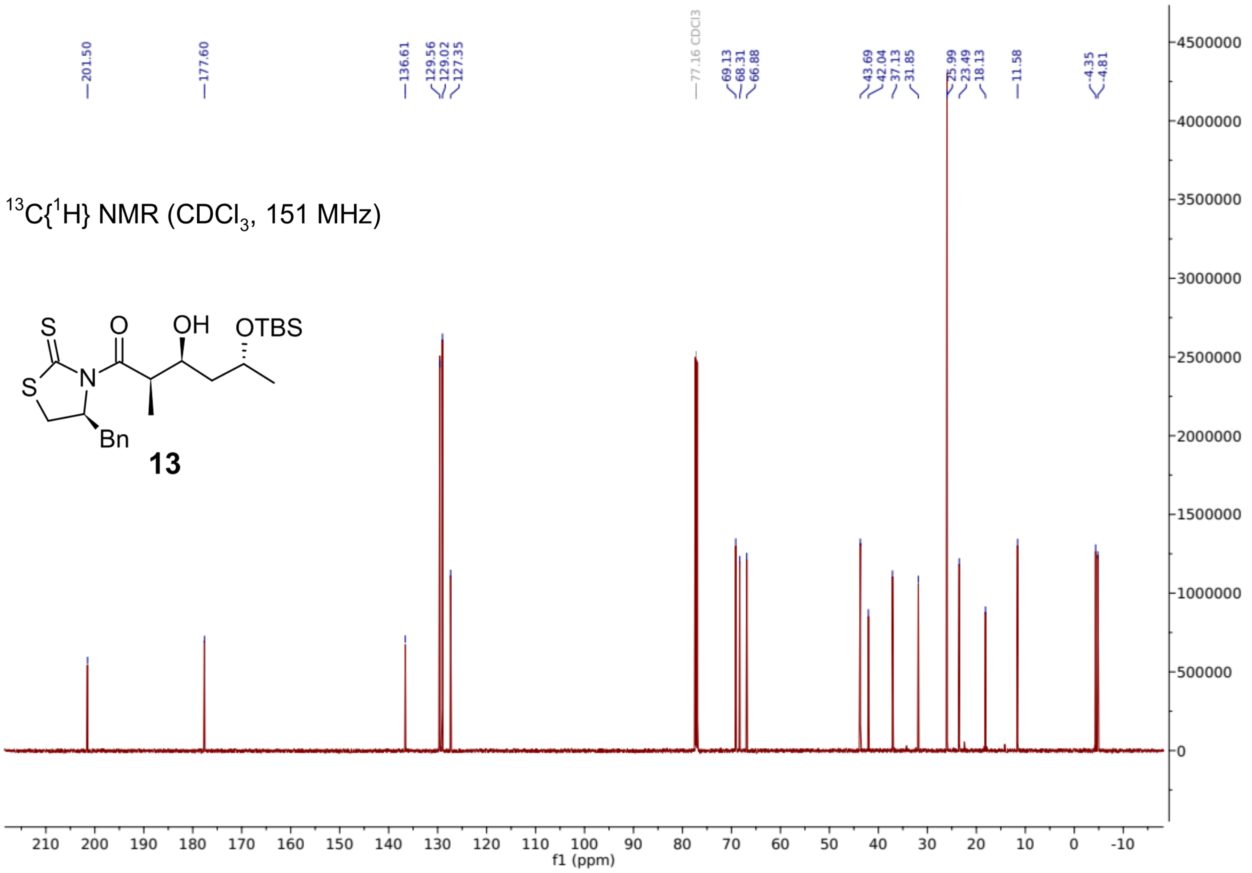

13C{1H} NMR (CDCl3, 151 MHz)
S
O
OH
OTBS
N
S
Bn
13
f1 (ppm)

(3*R*,4*S*,6*R*)-4-((*tert*-butyl(dimethyl)silyl)oxy)-3,6-dimethyloxan-2-one (15)

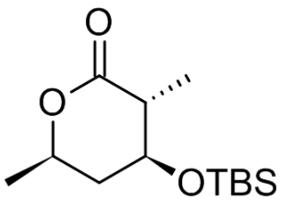

15

The aldol product **13** (2.544 g, 5.45 mmol, 1.0 eq.) is dissolved in dry dichloromethane (18.2 ml) and cooled to 0 °C. *Para*-toluenesulfonic acid monohydrate (553 mg, 2.9 mmol, 0.53 eq.) is added in portions and the solution is stirred for 1 h at 0 °C. After 30 h stirring at room temperature the colorless solution is again cooled to 0 °C and imidazole (1.11 g, 16.34 mmol, 3.0 eq.) and TBSCl (1.23 g, 8.17 mmol, 1.5 eq.) are introduced sequentially in portions. The reaction mixture is allowed to stir 10 min at 0 °C before it is warmed to room temperature and continues for another 20 h. Under vigorous stirring saturated aqueous $NaHCO_3$ solution (11 ml) is added to the solution, which is extracted with dichloromethane (3x10 ml). The organic layer is washed with water (2x15 ml), dried over $MgSO_4$, filtered and concentrated under reduced pressure. After column chromatography (*n*-pentane/Et_2O, 9:1) lactone **15** is provided (1.13 g, 4.376 mmol, 80 %) as a colorless crystalline solid.

General data: R_f = 0.3 (*n*-pentane/Et_2O, 3:1), $[\alpha]^{20}_D$ = +42.233 (c=0.206, $CHCl_3$),
m. p. = 41 °C, MW = 258.43 g/mol.

IR (neat): 2978 (w), 2954 (m), 2929 (m), 2887 (w), 2856 (m), 1725 (m), 1252 (m), 1231 (m), 1193 (m), 1105 (s), 1090 (s), 1060 (m), 869 (s), 836 (s), 803 (m), 775 (s), 671 (m) cm^{-1}.

^{1}H NMR ($CDCl_3$, 600 MHz): δ 4.32 (dqd, 1H, *J* = 12.5, 6.3, 2.9 Hz), 3.68 (ddd, 1H, *J* = 10.9, 9.6, 4.0 Hz), 2.33 (dq, 1H, *J* = 9.1, 7.1 Hz), 2.06 (ddd, 1H, *J* = 13.4, 4.0, 2.9 Hz), 1.64 (dt, 1H, *J* = 13.3, 11.3 Hz), 1.37 (d, 3H, *J* = 6.3 Hz), 1.32 (d, 3H, *J* = 7.1 Hz), 0.88 (s, 9H), 0.08 (s, 6H).

^{13}C{^{1}H} NMR ($CDCl_3$, 151 MHz): δ 173.9, 72.9, 70.9, 45.4, 40.9, 25.8, 22.0, 18.0, 13.8, -4.1, -4.7.

HRMS (ESI): calculated for $C_{13}H_{27}O_3Si$ $[M+H]^+$: 259.1729, found: 259.1733.

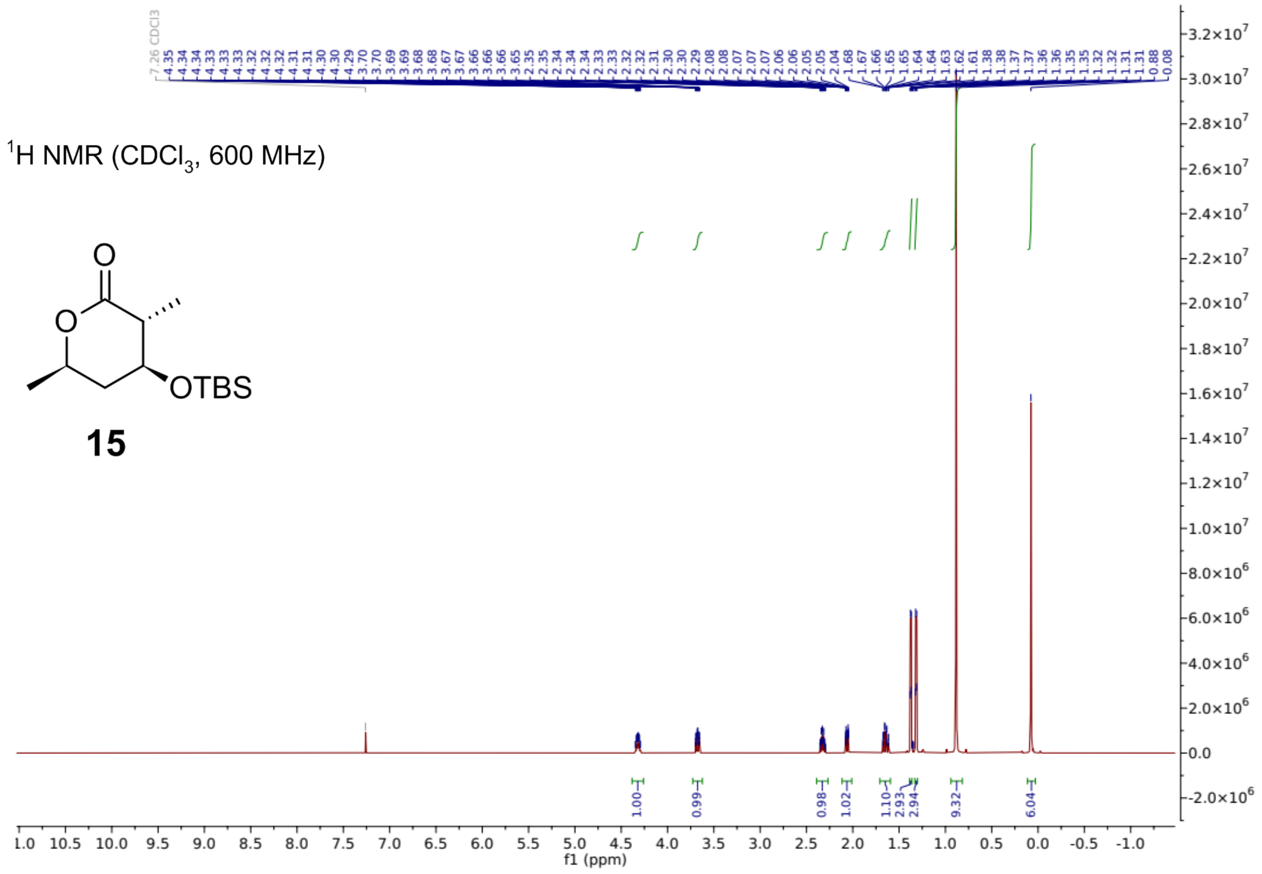
1H NMR (CDCl3, 600 MHz)
O
O
OTBS
15
1.00
0.99
0.98
1.02
1.10
2.93
2.94
9.32
6.04
f1 (ppm)

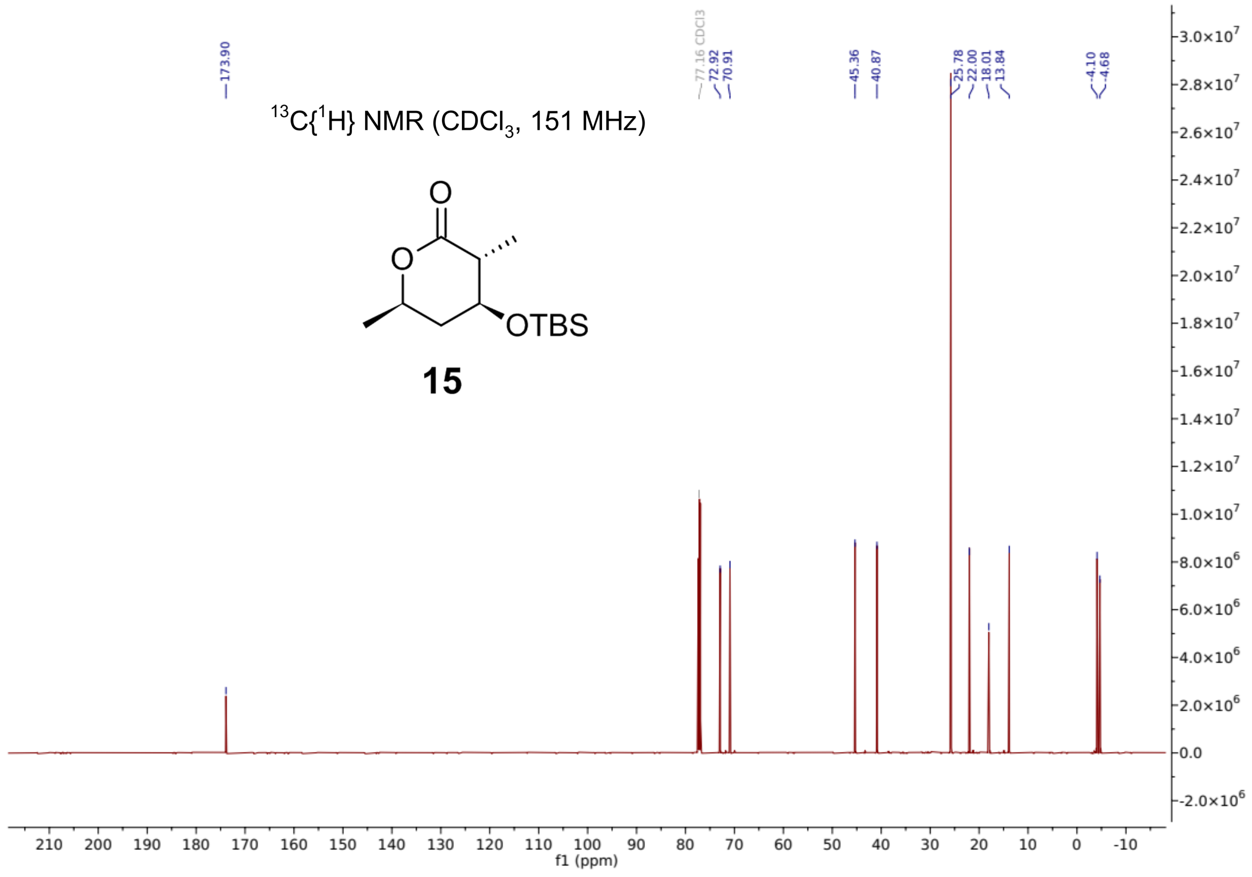
13C{1H} NMR (CDCl3, 151 MHz)
173.90
77.16 CDCl3
72.92
70.91
45.36
40.87
25.78
22.00
18.01
13.84
-4.10
-4.68
O
O
OTBS
15
f1 (ppm)

(3*S*,4*S*,6*R*)-4-((*tert*-butyl(dimethyl)silyl)oxy)-3-((1*R*)-1-hydroxy-3-((4-methoxybenzyl)oxy)propyl)-3,6-dimethyloxan-2-one (18)

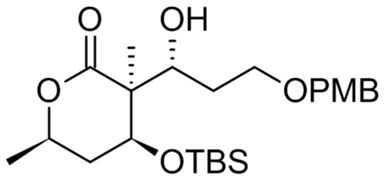

18

To a solution of lactone **15** (517 mg, 2.0 mmol, 1.0 eq.) in dry dichloromethane (9.2 ml) 1M Bu_2BOTf in dichloromethane (2.2 ml, 2.2 mmol, 1.1 eq.) is injected at -78 °C under nitrogen atmosphere. After stirring of the yellow solution for 20 min at -78 °C triethylamine (0.36 ml, 2.6 mmol, 1.3 eq.) is added slowly which leads to a colorless solution. The reaction mixture is allowed to stir 1 h at this temperature. Then a mixture of 3-((4-methoxybenzyl)oxy)propanal **(16)** (for preparation see ref. S1) (777 mg, 4.0 mmol, 2.0 eq.) with anhydrous dichloromethane (1.0 ml) is added slowly dropwise within 20 min. After stirring of the colorless solution for 3 h at -78 °C the cold reaction mixture is quenched sequentially with 20 mM aqueous phosphate buffer (8 ml), methanol (4 ml) and 30 % H_2O_2 (2 ml) at -78 °C. The cooling bath is removed and vigorous stirring is continued for additional 1.5 h. Dichloromethane (10 ml) is added and the organic layer is separated. The aqueous layer is extracted with dichloromethane (3x20 ml) and the combined organic layers are washed once with an aqueous saturated Na_2SO_3 solution (13 ml). After drying over $MgSO_4$, filtration and removing of the solvent the residue is purified by column chromatography (*n*-pentane/Et_2O, 2:1) to afford product **18** (582 mg, 1.28 mmol, 64 %) as a colorless oil.

General data: R_f = 0.2 (*n*-pentane/Et_2O, 1:1), $[\alpha]^{20}_D$ = +8.911 (c=0.202, $CHCl_3$),
MW = 452.66 g/mol.

IR (neat): 3496 (w br), 2954 (m), 2931 (m), 2886 (w), 2856 (m), 1716 (s), 1612 (w), 1513 (s), 1464 (m), 1388 (m), 1245 (s), 1214 (m), 1173 (m), 1102 (s), 1075 (s), 1034 (m), 836 (s), 776 (s) cm^{-1}.

^{1}H NMR ($CDCl_3$, 600 MHz): δ 7.25 - 7.21 (m, 2H), 6.88 - 6.84 (m, 2H), 4.51 - 4.40 (m, 3H), 4.28 (dd, 1H, *J* = 11.0, 1.9 Hz), 3.95 (dd, 1H, *J* = 9.4, 4.6 Hz), 3.80 (s, 3H), 3.65 (dd, 2H, *J* = 6.5, 5.8 Hz), 2.31 - 2.22 (m, 1H), 1.97 (dt, 1H, *J* = 13.5, 4.8 Hz), 1.91 (ddt, 1H, *J* = 14.1, 11.0, 6.5 Hz), 1.77 (dtd, 1H, *J* = 13.9, 5.8, 1.9 Hz), 1.40 (d, 3H, *J* = 6.3 Hz), 1.25 (s, 3H), 0.89 (s, 9H), 0.11 (s, 3H), 0.08 (s, 3H).

^{13}C{^{1}H} NMR ($CDCl_3$, 151 MHz): δ 175.3, 159.3, 130.4, 129.4, 113.9, 74.0, 73.2, 72.9, 72.2, 69.0, 55.4, 51.2, 37.0, 31.7, 25.9, 22.7, 19.1, 18.1, -3.7, -4.8.

HRMS (ESI): calculated for $C_{24}H_{41}O_6Si$ $[M+H]^+$: 453.2672, found: 453.2670.

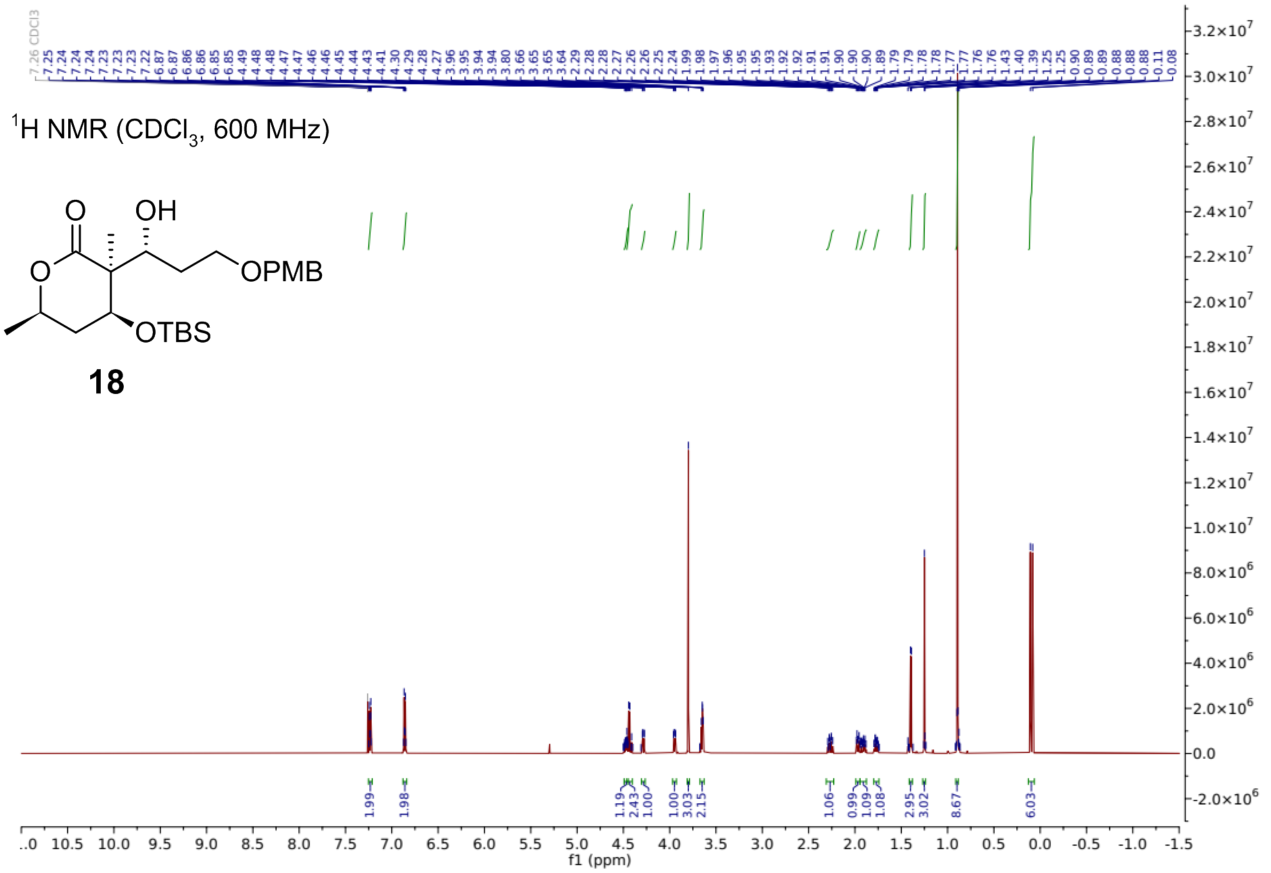

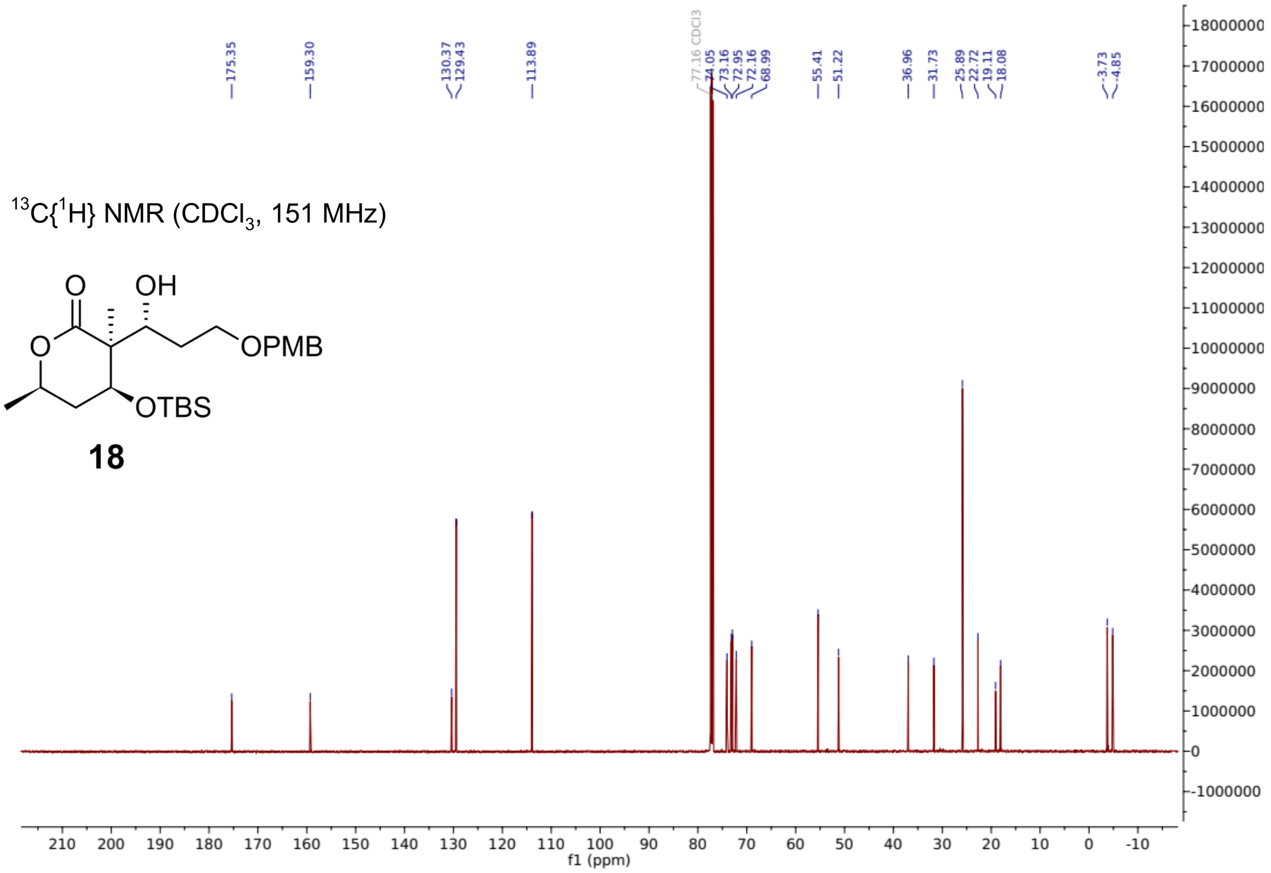

(3*S*,4*S*,6*R*)-4-hydroxy-3-((1*R*)-1-hydroxy-3-((4-methoxybenzyl)oxy)propyl)-3,6-dimethyloxan-2-one (21)

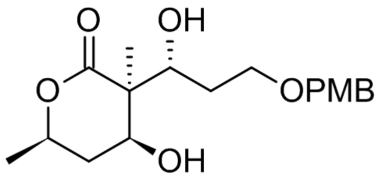

21

Compound **18** (36.4 mg, 0.08 mmol, 1.0 eq.) is dissolved in anhydrous THF (1 ml), cooled to 0 °C and 1M TBAF in THF (0.1 ml, 0.1 mmol, 1.25 eq.) is added. After 30 min stirring at 0 °C the colorless reaction solution is quenched with water (1.5 ml) and extracted with ethyl acetate (3x2 ml). The combined organic layers are dried over $MgSO_4$, filtered and concentrated under reduced pressure. After purification by column chromatography (freshly distilled diethyl ether, 100%) diol **21** is provided (15 mg, 0.044 mmol, 55 %) as a colorless crystalline solid.

In order to form crystals, which can be measured in X-ray, the crystalline solid is dissolved with some drops of freshly distilled anhydrous dichloromethane to create a high concentrated solution at room temperature. Then freshly distilled *n*-pentane is added dropwise until the solution becomes a suspension. At that point the *n*-pentane addition is stopped to let the floating crystals grow while the solvent is allowed to evaporate slowly at room temperature. After that short thin crystals like needles are obtained.

General data: R_f = 0.25 (Et_2O), $[\alpha]^{20}_D$ = +22.115 (c=0.104, $CHCl_3$), m. p. = 107 °C, MW = 338.4 g/mol.

IR (neat): 3327 (m br), 3272 (m br), 2970 (m), 2919 (m), 2858 (w), 1720 (m), 1514 (m), 1303 (m), 1249 (m), 1222 (m), 1129 (m), 1081 (s), 1057 (m), 1041 (m), 1028 (m), 954 (m), 882 (m), 815 (m), 619 (m), 574 (m), 522 (m) cm^{-1}.

^{1}H NMR ($CDCl_3$, 600 MHz): δ 7.25 - 7.20 (m, 2H), 6.91 - 6.85 (m, 2H), 4.53 (dqd, 1H, *J* = 9.2, 6.4, 4.9 Hz), 4.47 - 4.45 (m, 2H), 4.25 (dd, 1H, *J* = 9.2, 1.8 Hz), 4.01 (dd, 1H, *J* = 7.9, 5.3 Hz), 3.81 (s, 3H), 3.72 (ddd, 1H, *J* = 9.1, 6.7, 3.7 Hz), 3.62 (ddd, 1H, *J* = 9.1, 7.8, 3.6 Hz), 2.24 (dt, 1H, *J* = 14.0, 5.1 Hz), 2.03 - 1.93 (m, 2H), 1.85 (dddd, 1H, *J* = 14.9, 6.7, 3.6, 1.8 Hz), 1.43 (d, 3H, *J* = 6.4 Hz), 1.32 (s, 3H).

^{13}C{^{1}H} NMR ($CDCl_3$, 151 MHz): δ 176.0, 159.6, 129.64, 129.60, 114.1, 75.7, 73.44, 73.36, 73.33, 69.7, 55.4, 49.7, 36.9, 32.1, 22.8, 19.5.

HRMS (ESI): calculated for $C_{18}H_{27}O_6$ $[M+H]^+$: 339.1808, found: 339.1812.

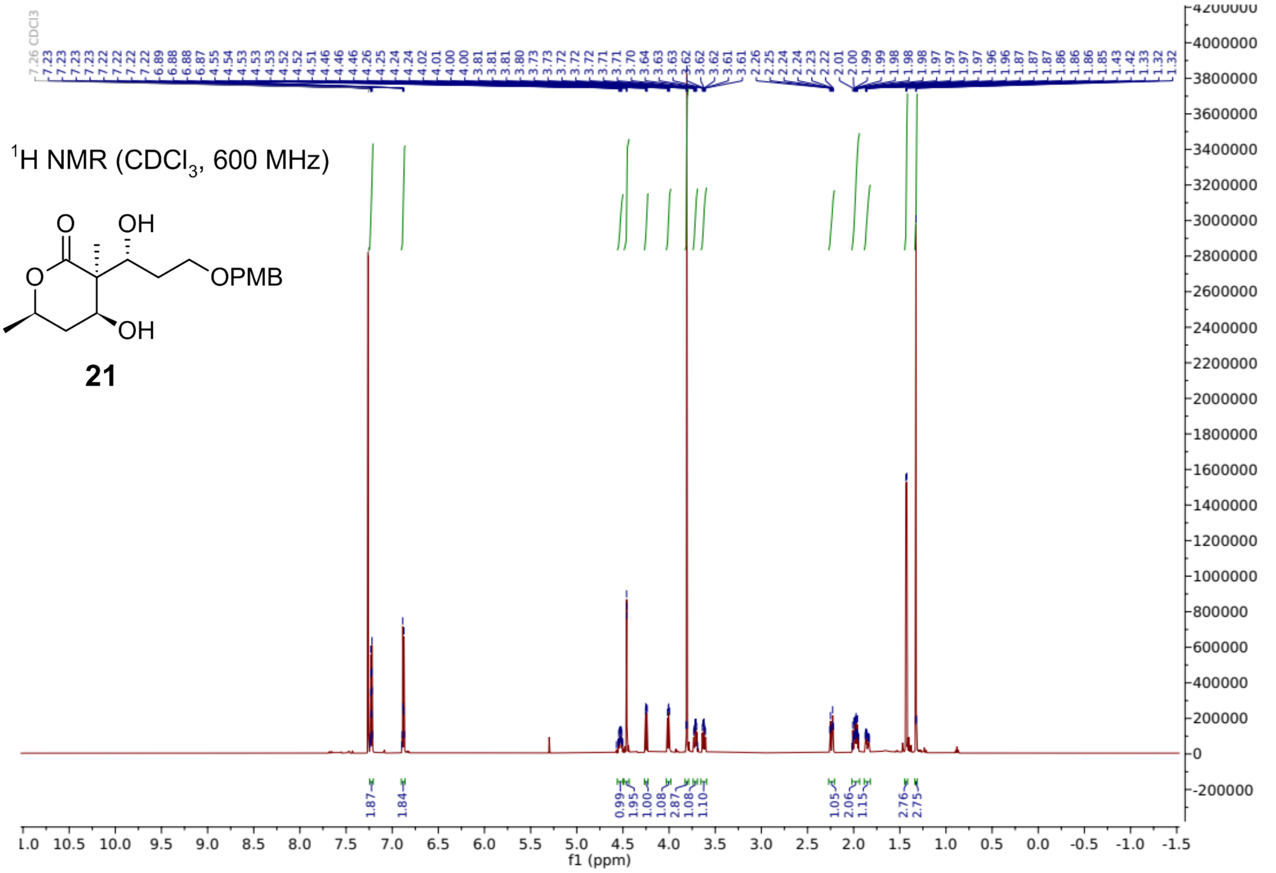

^{1}H NMR (CDCl$_3$, 600 MHz)
O
OH
O
OPMB
OH
21
f1 (ppm)

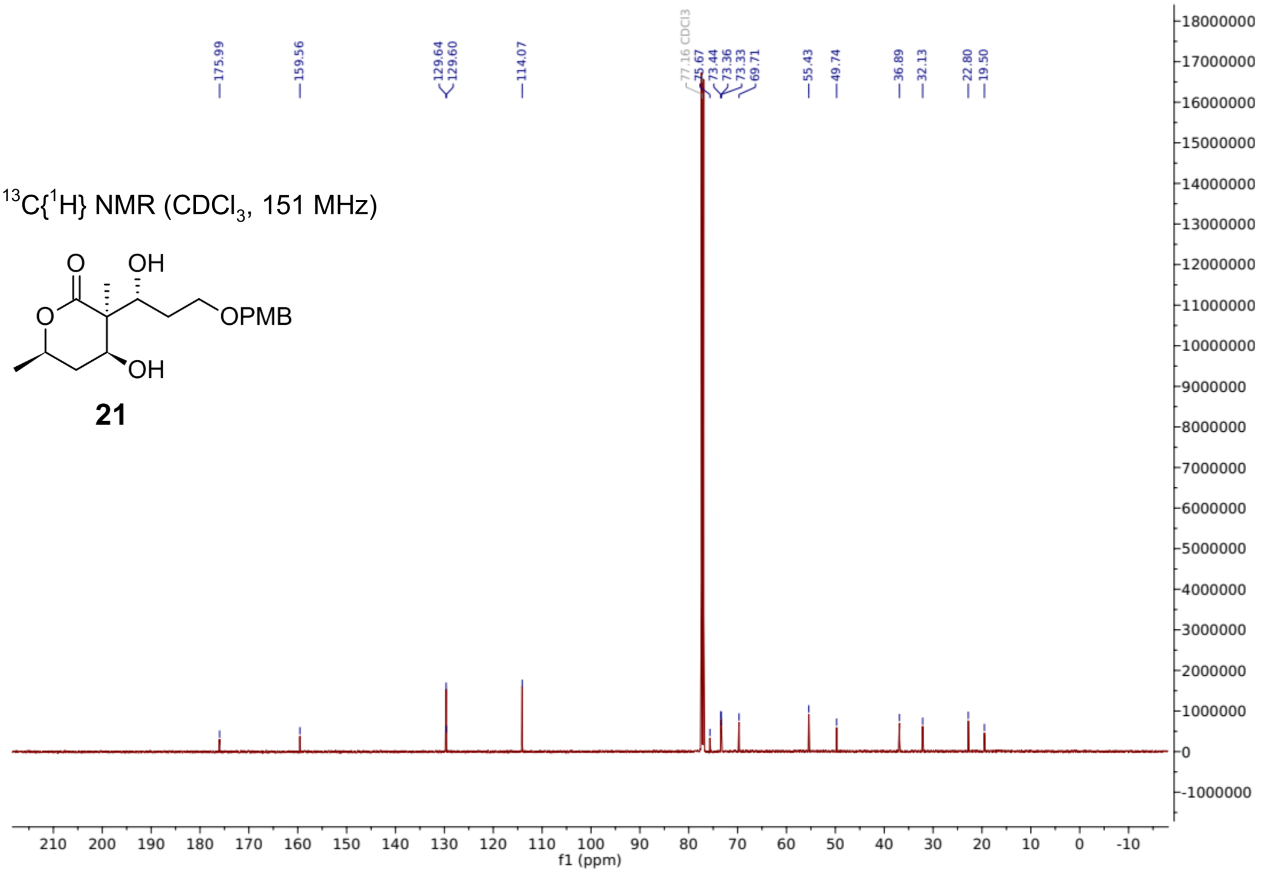

175.99
159.56
129.64
129.60
114.07
77.16 CDCl3
75.67
73.44
73.36
73.33
69.71
55.43
49.74
36.89
32.13
22.80
19.50
^{13}C{^{1}H} NMR (CDCl$_3$, 151 MHz)
O
OH
O
OPMB
OH
21
f1 (ppm)

Methyl (2*R*,3*S*,5*R*)-3-((*tert*-butyl(dimethyl)silyl)oxy)-5-hydroxy-2-((1*S*)-3-((4-methoxybenzyl)oxy)-1-((triethylsilyl)oxy)propyl)-2-methylhexanoate (25)
Methyl (2*R*,3*S*,5*S*)-3-((*tert*-butyl(dimethyl)silyl)oxy)-5-hydroxy-2-((1*S*)-3-((4-methoxybenzyl)oxy)-1-((triethylsilyl)oxy)propyl)-2-methylhexanoate (22)

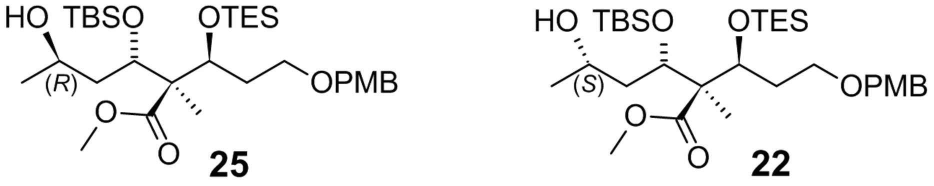

At room temperature mercury(II) acetate (35.1 mg, 0.11 mmol, 1.1 eq.) is dissolved in water (0.24 ml) and the flask is wrapped in aluminum foil. Addition of THF (0.36 ml) leads to an orange suspension. Then the terminal alkene **24** (58.1 mg, 0.1 mmol, 1.0 eq.) in THF (0.4 ml) is added dropwise and the resulted yellow suspension is stirred for 18 h. After that sodium borohydride (76 mg, 2 mmol, 20 eq.) is added in two portions which leads to a white suspension at room temperature and the mixture is allowed to stir for further 3 h. The reaction is quenched with brine (0.8 ml) and extracted with ethyl acetate (5x1.5 ml). The combined organic layers are dried over $MgSO_4$, filtered and concentrated under reduced pressure. The residue is purified by column chromatography (*n*-pentane/Et_2O, 6:1) to obtain an inseparable isomeric mixture **25** and **22** (15 mg, 0.025 mmol, 25%, R/S 3:1) as a colorless oil.

CAUTION! *Mercury(II) acetate is fatal and may cause damage to organs. We used a scale placed under a well ventilated fume hood for determining the quantity transferred to the reaction vessel.*

^{1}H NMR (C_6D_6, 400 MHz): δ 7.26 (d, 2H, *J* = 8.7 Hz), 7.23 (d, 6H, *J* = 8.6 Hz), 6.84 (d, 2H, *J* = 8.7 Hz), 6.80 (d, 6H, *J* = 8.6 Hz), 4.76 (dd, 4H, *J* = 9.5, 7.4 Hz), 4.39 - 4.26 (m, 8H), 4.10 (dd, 3H, *J* =8.7, 1.0 Hz), 4.05 - 3.99 (m, 1H), 3.93 - 3.84 (m, 3H), 3.79 (t, 1H, *J* =5.6 Hz), 3.64 - 3.53 (m, 4H), 3.51 (s, 9H), 3.48 (s, 3H), 3.45 (ddd, 1H, *J* =9.1, 5.5, 3.7 Hz), 3.37 (ddd, 3H, *J* =8.8, 4.2, 3.3 Hz), 3.31 (s, 3H), 3.31 (s, 9H), 2.22 - 1.99 (m, 9H), 1.86 (ddd, 3H, *J* =15.2, 8.7, 1.9 Hz), 1.83 - 1.74 (m, 1H), 1.59 (d, 3H, *J* =8.8 Hz), 1.54 (s, 10H), 1.47 (s, 3H), 1.45 - 1.39 (m, 3H), 1.11 (d, 3H, *J* =6.1 Hz), 1.07 (t, 36H, *J* =8.0 Hz), 1.00 (s, 27H), 0.99 (s, 9H), 0.95 (d, 9H, *J* =6.2 Hz), 0.83 - 0.59 (m, 24H), 0.22 (s, 9H), 0.16 (s, 9H), 0.13 (d, 6H, *J* =5.5 Hz).

^{13}C{^{1}H} NMR (C_6D_6, 101 MHz): δ 174.9, 174.7, 160.1, 159.9, 130.8, 130.4, 130.1, 129.7, 114.3, 114.2, 76.35, 76.32, 73.24, 73.18, 72.8, 72.0, 67.2, 67.0, 64.9, 64.6, 58.7, 57.6, 54.8, 51.1, 50.9, 43.6, 43.1, 33.9, 33.2, 26.4, 26.2, 25.1, 24.3, 18.8, 18.5, 15.6, 15.2, 7.38, 7.36, 6.03, 5.96, -3.32, -3.35, -3.9, -4.1.

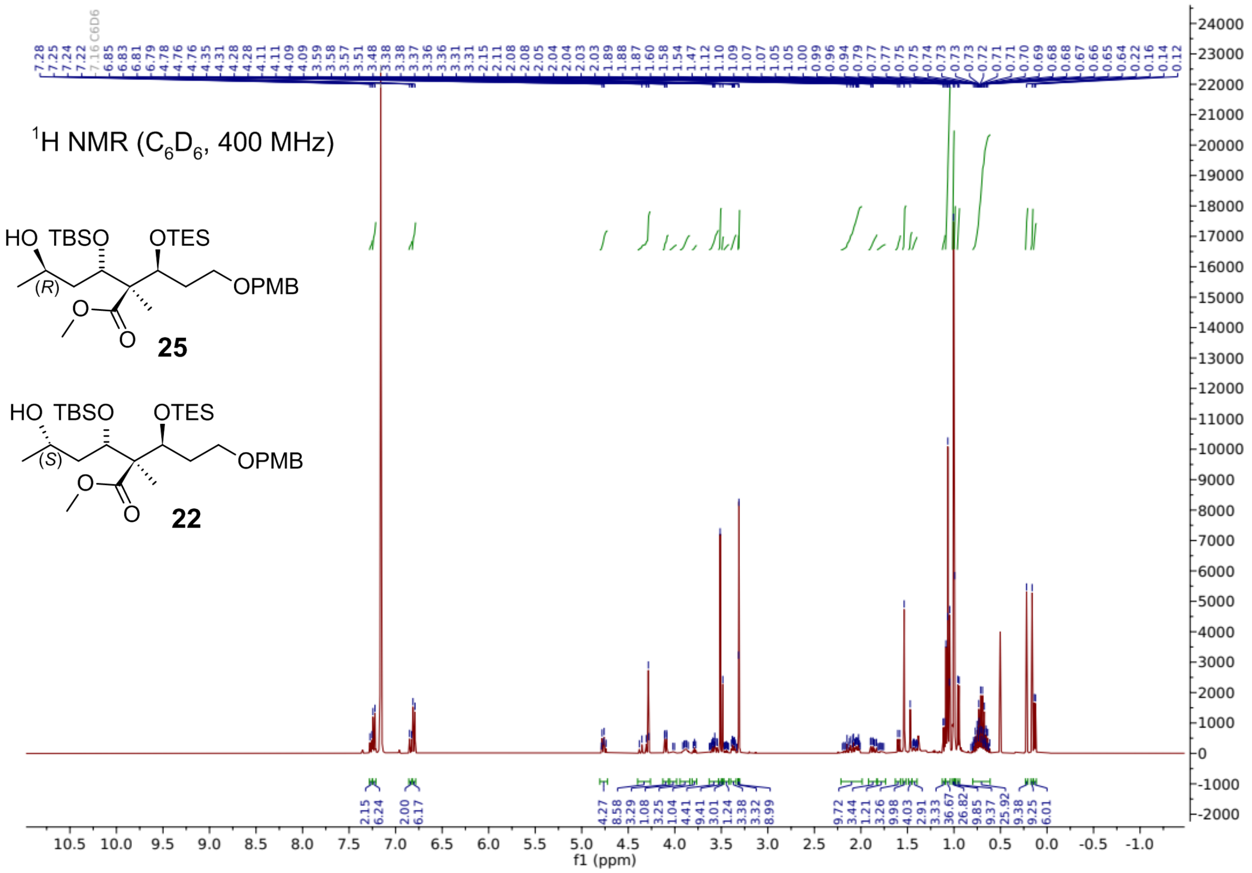

^{1}H NMR (C_6D_6, 400 MHz)
HO TBSO OTES
(R)
OPMB
25
HO TBSO OTES
(S)
OPMB
22
f1 (ppm)

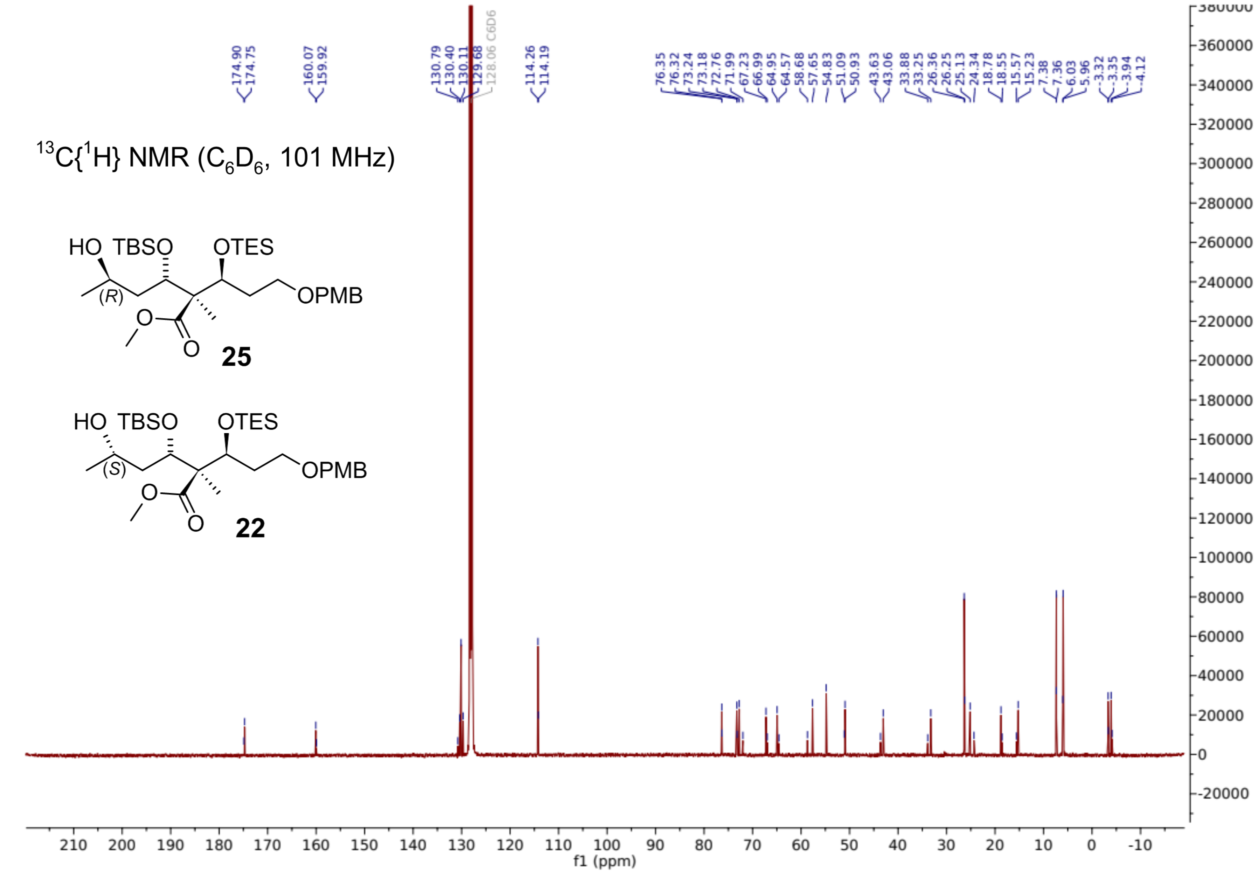

^{13}C{^{1}H} NMR (C_6D_6, 101 MHz)
HO TBSO OTES
(R)
OPMB
25
HO TBSO OTES
(S)
OPMB
22
f1 (ppm)

Table S1. Comparison of the $^{13}C\{^{1}H\}$-NMR data for structure proof of <u>22</u>			
<u>R/S mixture 25/22</u>	<u>Compound 22</u>	<u>R/S mixture 25/22</u>	<u>Compound 22</u>
174.90	174.89	51.09	51.09
174.75		50.93	
160.07		43.63	43.63
159.92	159.92	43.06	
130.79	130.79	33.88	33.88
130.40		33.25	
130.11		26.36	
129.68	129.68	26.25	26.25
114.26		25.13	
114.19	114.19	24.34	24.35
76.35		18.78	
76.32	76.32	18.55	18.55
73.24		15.57	15.57
73.18	73.18	15.23	
72.76		7.38	7.38
71.99	71.99	7.36	
67.23		6.03	6.03
66.99	66.99	5.96	
64.95		-3.32	
64.57	64.57	-3.35	-3.34
58.68	58.68	-3.94	
57.65		-4.12	-4.12
54.83	54.82		

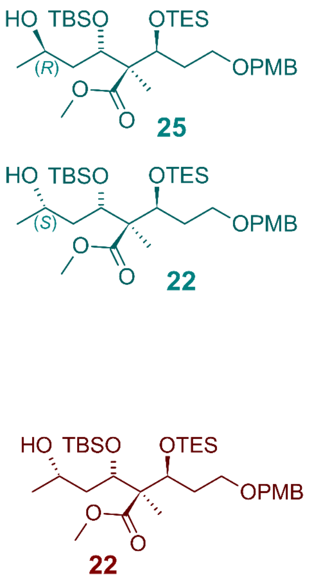
HO TBSO OTES
(R)
OPMB
O
O
25
HO TBSO OTES
(S)
OPMB
O
O
22
HO TBSO OTES
OPMB
O
O
22

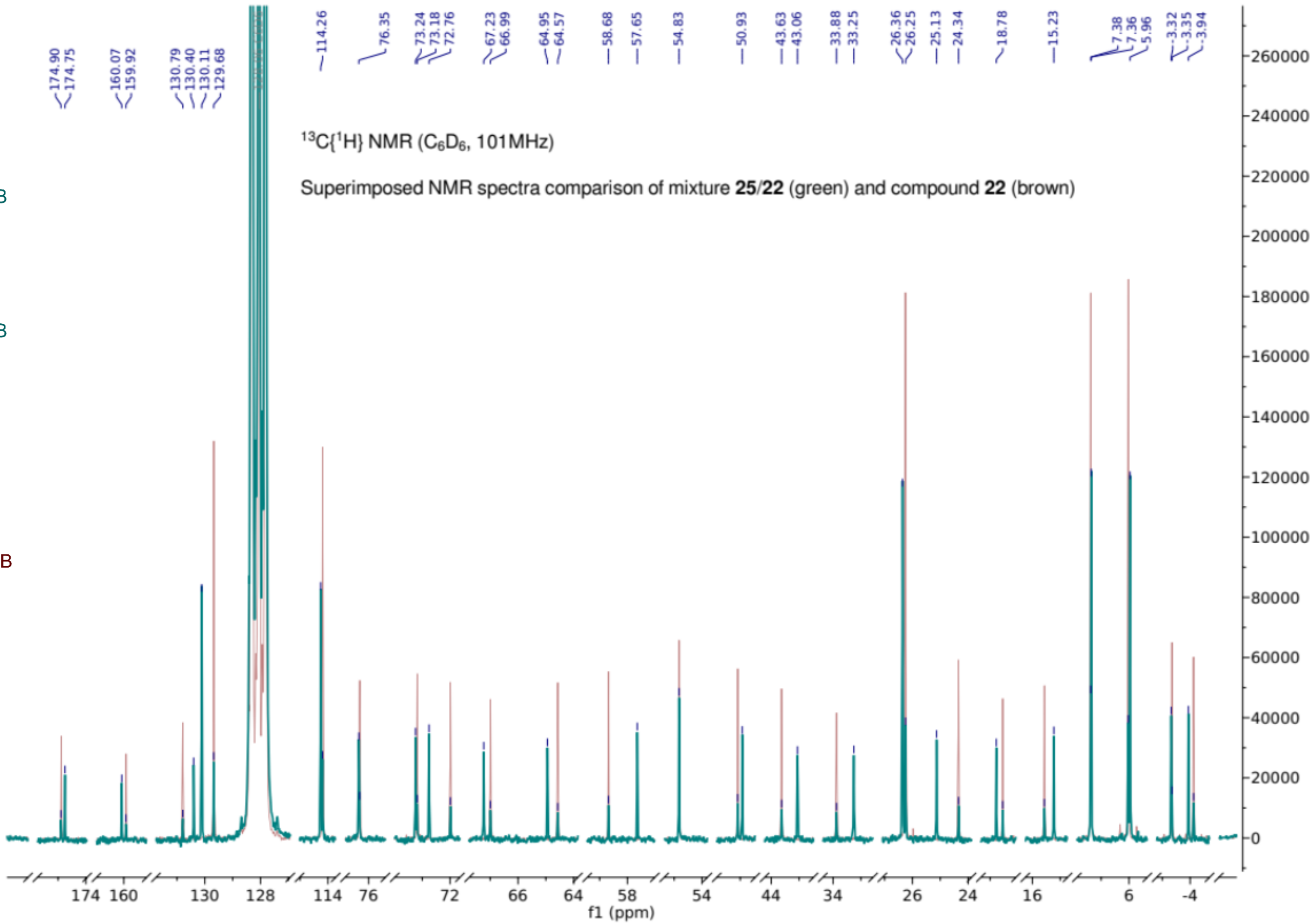
$^{13}C\{^1H\}$ NMR (C_6D_6, 101MHz)
Superimposed NMR spectra comparison of mixture **25**/**22** (green) and compound **22** (brown)
174.90
174.75
160.07
159.92
130.79
130.40
130.11
129.68
114.26
76.35
73.24
73.18
72.76
67.23
66.99
64.95
64.57
58.68
57.65
54.83
50.93
43.63
43.06
33.88
33.25
26.36
26.25
25.13
24.34
18.78
15.23
7.38
7.36
5.96
-3.32
-3.35
-3.94
260000
240000
220000
200000
180000
160000
140000
120000
100000
80000
60000
40000
20000
0
174
160
130
128
114
76
72
66
64
58
54
44
34
26
24
16
6
-4
f1 (ppm)

(3*R*,4*S*,6*R*)-4-((*tert*-butyl(dimethyl)silyl)oxy)-3-((1*S*,2*E*)-1-hydroxybut-2-en-1-yl)-3,6-dimethyloxan-2-one (19)

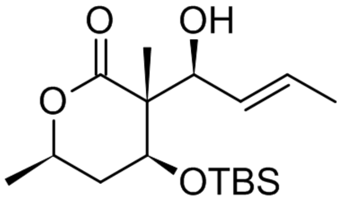

19

To a solution of lactone **15** (517 mg, 2.0 mmol, 1.0 eq.) in dry dichloromethane (9.2 ml) 1M Bu_2BOTf in dichloromethane (2.2 ml, 2.2 mmol, 1.1 eq.) is injected at -78 °C under nitrogen atmosphere. After stirring of the yellow solution for 20 min at -78 °C triethylamine (0.36 ml, 2.6 mmol, 1.3 eq.) is added slowly which leads to a colorless solution. The reaction mixture is allowed to stir 1 h at this temperature. Then a mixture of crotonaldehyde (0.33 ml, 4.0 mmol, 2.0 eq.) with anhydrous dichloromethane (0.67 ml) is added slowly dropwise within 20 min. After stirring of the colorless solution for 3 h at -78 °C the cold reaction mixture is quenched sequentially with 20 mM aqueous phosphate buffer (8 ml), methanol (4 ml) and 30 % H_2O_2 (2 ml) at -78 °C. The cooling bath is removed and vigorous stirring is continued for additional 1.5 h. Dichloromethane (10 ml) is added and the organic layer is separated. The aqueous layer is extracted with dichloromethane (3x20 ml) and the combined organic layers are washed once with an aqueous saturated Na_2SO_3 solution (11 ml). After drying over $MgSO_4$, filtration and removing of the solvent the residue is purified by column chromatography (*n*-pentane/Et_2O, 3:1) to afford product **19** (354 mg, 1.1 mmol, 54 %) as colorless crystalline solid.
In order to measure in X ray, please see method of lactone **8** to make crystals.

General data: R_f = 0.4 (*n*-pentane/Et_2O, 1:1), $[\alpha]^{20}_D$ = +3.237 (c=0.278, $CHCl_3$),
m. p. = 75 - 76 °C, MW = 328.52 g/mol.

IR (neat): 3443 (m br), 2929 (m), 2856 (m), 1715 (s), 1212 (m), 1125 (m), 1107 (s), 1083 (s), 969 (s), 854 (s), 834 (s), 776 (s), 610 (m) cm^{-1}.

^{1}H NMR ($CDCl_3$, 400 MHz): δ 6.0 (ddq, 1H, *J* = 15.2, 8.0, 1.6 Hz), 5.7 (dqd, 1H, *J* = 15.3, 6.5, 0.9 Hz), 4.4 (dd, 1H, *J* = 11.7, 4.4 Hz), 4.4 - 4.3 (m, 1H), 4.1 (d, 1H, *J* = 8.0 Hz), 1.9 (ddd, 1H, *J* = 13.4, 4.4, 3.2 Hz), 1.9 - 1.8 (m, 2H), 1.7 (dd, 3H, *J* = 6.6, 1.6 Hz), 1.4 (d, 3H, *J* = 6.3 Hz), 1.1 (s, 3H), 0.9 (s, 9H), 0.1 (s, 3H), 0.1 (s, 3H).

^{13}C{^{1}H} NMR ($CDCl_3$, 101 MHz): δ 175.4, 130.8, 130.2, 76.6, 72.9, 68.4, 53.4, 36.8, 25.8, 21.9, 18.1, 17.9, 17.1, -3.7, -4.8.

HRMS (ESI): calculated for $C_{17}H_{33}O_4Si$ $[M+H]^+$: 329.2148, found: 329.2139.

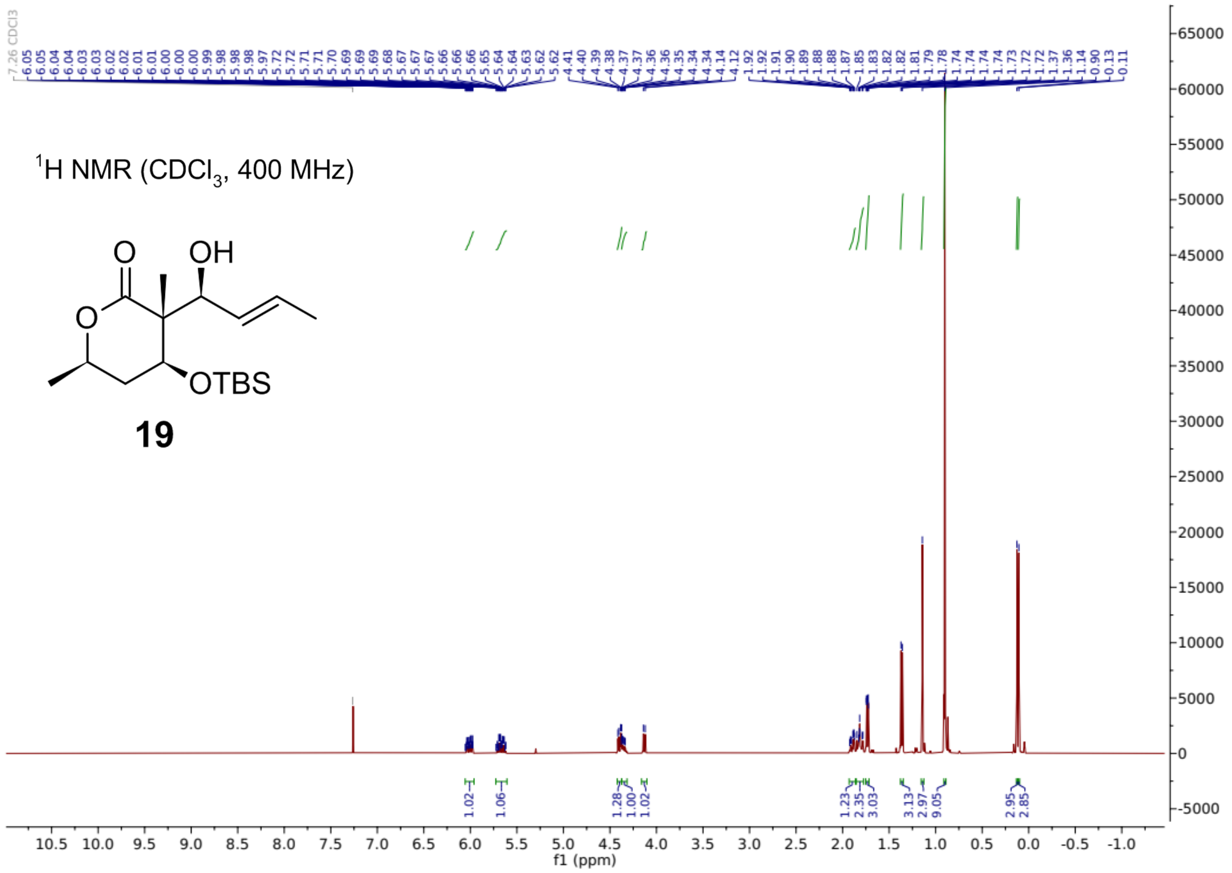

^{1}H NMR ($CDCl_3$, 400 MHz)
OTBS
19
f1 (ppm)

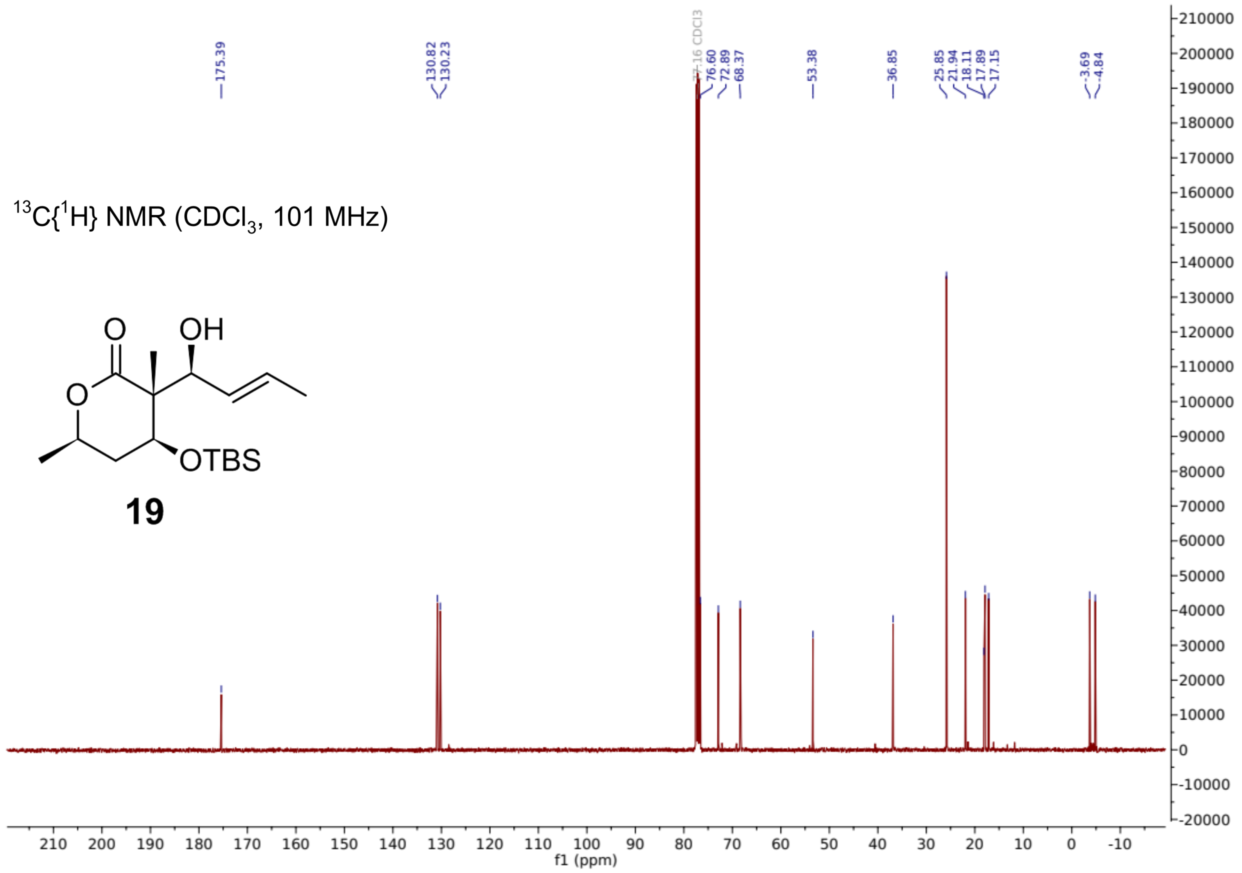

^{13}C{^{1}H} NMR ($CDCl_3$, 101 MHz)
OTBS
19
f1 (ppm)

Single-crystal X-ray structural analyses

The single-crystal X-ray intensity data for the reported compounds were collected on diffractometers with Mo-Kα or Cu-Kα sources (see Table S2). The crystal structures were solved with SHELXT-2018/3[S4] and refined by full matrix least-squares methods on F^2 with SHELXL-2018/3,[S5] using the Olex2 environment.[S6] Multi-scan absorption correction was applied to the intensity data.[S7,S8] Restraints on the O-H distance within hydroxy groups were used for compounds **8**, **13**, **19**, **20**, and **21**.[S5] For **8**, **13**, and **14**, the absolute structure could be determined unambiguously by anomal dispersion.[S9] This was not possible for **20** and **21** due to the absence of heavy atoms, but the correctness of the refined absolute structures could be ensured though the knowledge of unchanged stereocenters during chemical synthesis. The same is true for compound **19**, where a reliable absolute-structure determination was disrupted by twinning. The unit cell parameters suggested that this structure is orthorhombic, but the structure could not be solved and refined in this crystals system. The structure was solved in P1, transformed to $P2_1$,[S10] and refined to good R values by applying the twin law (1 0 0 0 -1 0 0 0 -1).[S6] CCDC 2472860-2472865 contain the supplementary crystallographic data for this paper (see Table S2). These data can be obtained free of charge from The Cambridge Crystallographic Data Center (CCDC; http://www.ccdc.cam.ac.uk).

Note: The CheckCIF report for compound **20** possesses a level-B alert due to a low precision on the determined C-C bond lengths, which could not be eliminated during structure refinement. This can be addressed to the limited data quality due to crystallization of the compound as very fine needles and the absence of heavy atoms. However, the interatomic connectivity and the relative configuration of the stereocenters could be determined reliably.

Table S2. Crystal data and details on structure refinement for the reported compounds.

**Compound**	**8**	**13**	**14**	**19**	**20**	**21**
CCDC deposition number	2472860	2472861	2472862	2472863	2472864	2472865
Molecular formula sum	$C_{16}H_{30}O_4Si$	$C_{23}H_{37}NO_3S_2Si$	$C_{13}H_{26}O_3Si$	$C_{17}H_{32}O_4Si$	$C_{18}H_{26}O_6$	$C_{18}H_{26}O_6$
Formula weight / g mol^{-1}	314.49	467.74	258.43	328.51	338.39	338.39
Crystal system	orthorhombic	orthorhombic	orthorhombic	monoclinic	monoclinic	monoclinic
Space group	$P2_12_12_1$	$P2_12_12_1$	$P2_12_12_1$	$P2_1$	$P2_1$	$P2_1$
Cell metric a / Å	8.1091(2)	7.595(2)	6.3430(8)	7.7530(9)	10.084(1)	10.1578(6)
b / Å	9.5282(3)	13.631(3)	11.700(2)	26.363(4)	6.6564(4)	5.8895(5)
c / Å	23.4743(6)	50.35(1)	20.923(3)	9.541(1)	13.528(1)	14.0596(8)
α / deg.	90	90	90	90	90	90
β / deg.	90	90	90	90.07(1)	107.940(8)	93.879(5)
γ / deg.	90	90	90	90	90	90
Cell volume / Å^3	1813.74(9)	5212.5(19)	1552.8(3)	1950.0(5)	863.8(1)	839.2(1)
Molecules per cell z	4	8	4	4	2	2
Electrons per cell F_{000}	688	2016	568	720	364	364
Calcd. density ρ / g cm^{-3}	1.152	1.192	1.105	1.119	1.301	1.339
μ / mm^{-1} (radiation)	0.140 (Mo-K_α)	0.273 (Mo-K_α)	0.148 (Mo-K_α)	0.134 (Mo-K_α)	0.801 (Cu-K_α)	0.824 (Cu-K_α)
Crystal shape and color	colorless plate	yellow plate	colorless prism	colorless plank	colorless needle	colorless plate
Crystal size / mm	0.31×0.28×0.11	0.24×0.05×0.03	0.21×0.19×0.12	0.22×0.09×0.03	0.62×0.05×0.02	0.42×0.09×0.04
Diffractometer	STOE IPDS 2T	Bruker KappaCCD	Bruker KappaCCD	Bruker KappaCCD	STOE IPDS 2T	STOE IPDS 2T
T / K	133(2)	120(2)	120(2)	120(2)	130(2)	130(2)
θ range / deg.	2.307 … 27.382	1.548 … 28.282	1.947 … 25.098	0.772 … 27.289	3.434 … 66.565	3.151 … 66.591
Reflections collected	12506	52543	14946	17542	8059	8589
Reflections unique	4088	12727	2755	7768	2981	2883
Reflections with $I>2\sigma(I)$	3688	11864	2123	6316	1802	2590
Completeness of dataset	99.9 %	99.9 %	99.6 %	99.3 %	99.1 %	98.5 %
R_{int}	0.0508	0.0275	0.0623	0.0544	0.0506	0.0199
Parameters; Restraints	200; 1	561; 2	161; 6	420; 3	223; 1	227; 1
R_1 (all data, $I>2\sigma(I)$)	0.0476; 0.0395	0.0334; 0.0295	0.0920; 0.0617	0.0644; 0.0472	0.1513; 0.0719	0.0448; 0.0365
wR_2 (all data, $I>2\sigma(I)$)	0.0988; 0.0932	0.0703; 0.0688	0.1617; 0.1483	0.0878; 0.0821	0.1731; 0.1257	0.0905; 0.0836
GooF (F^2)	1.081	1.058	1.202	0.985	1.105	1.094
Max. residual peaks	–0.235; 0.214	–0.181; 0.305	–0.300; 0.281	–0.246; 0.302	–0.318; 0.296	–0.172; 0.149
Flack parameter[S9]	–0.09(8)	0.02(1)	0.08(9)	0.0(1)	–0.2(5)	0.3(2)

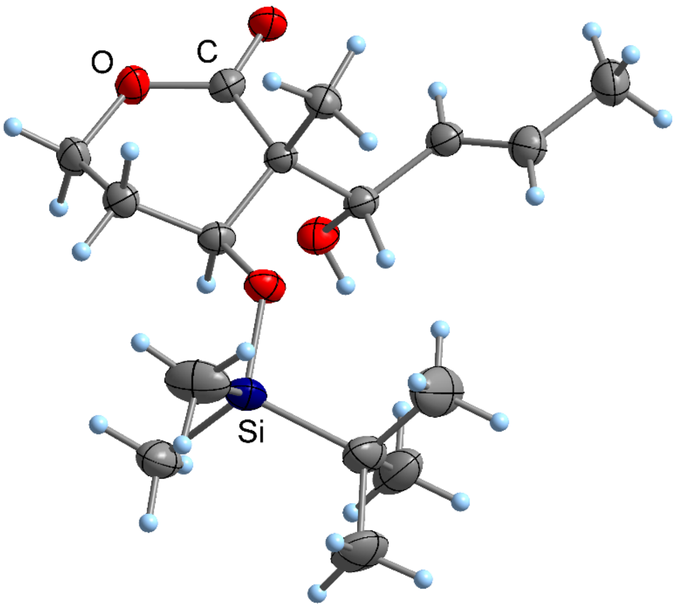

Figure S1. Molecular structure of compound **8** in the crystal. Displacement ellipsoids are drawn at the 50% probability level, H atoms as spheres of arbitrary size.

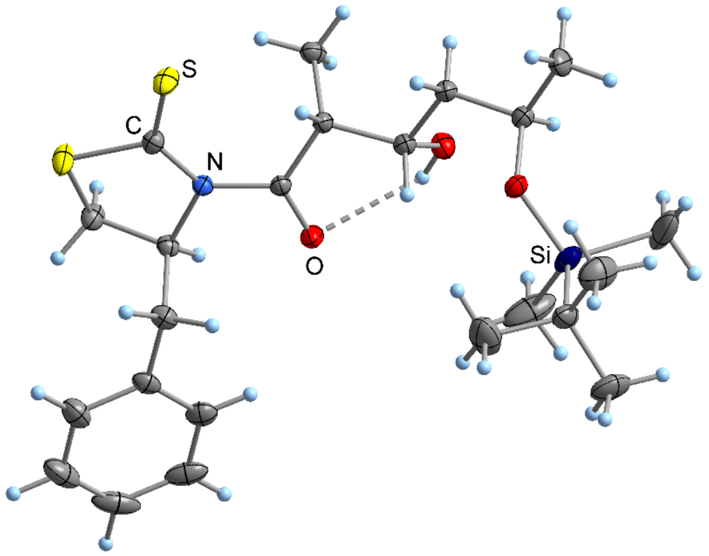

Figure S2. Molecular structure of compound **13** in the crystal. Displacement ellipsoids are drawn at the 50% probability level, H atoms as spheres of arbitrary size.

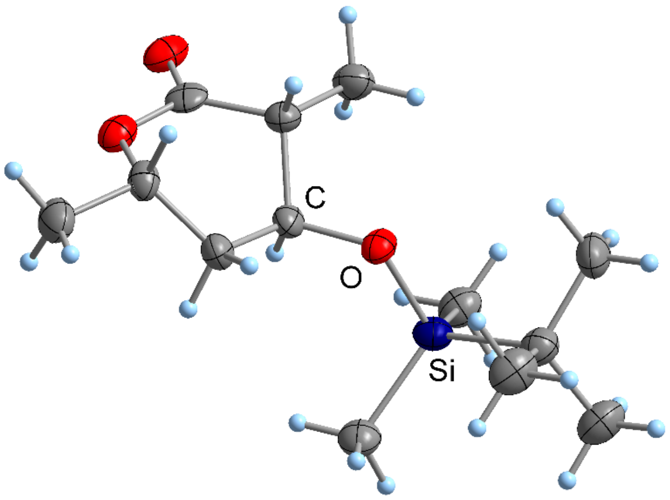

Figure S3. Molecular structure of compound **14** in the crystal. Displacement ellipsoids are drawn at the 50% probability level, H atoms as spheres of arbitrary size.

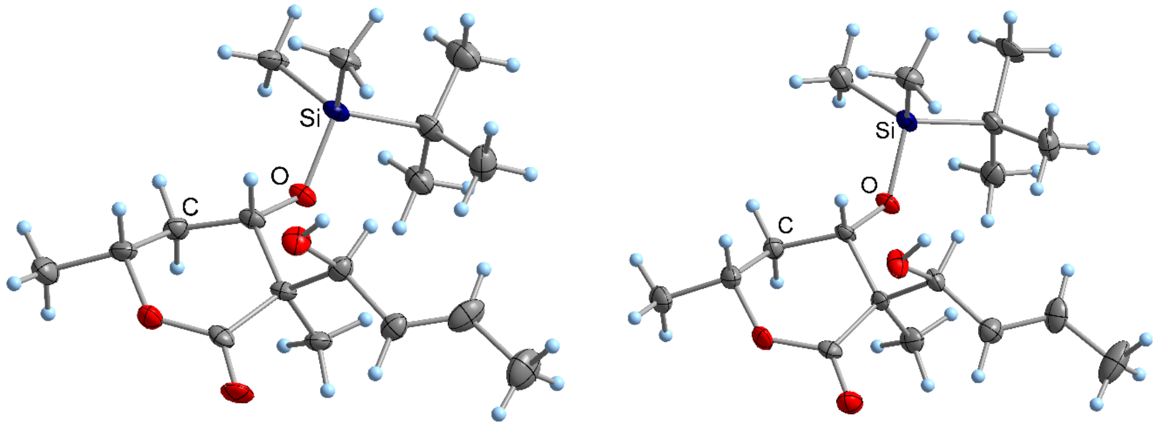

Figure S4. Molecular structure of compound **19** in the crystal (two molecules in the asymmetric unit). Displacement ellipsoids are drawn at the 50% probability level, H atoms as spheres of arbitrary size.

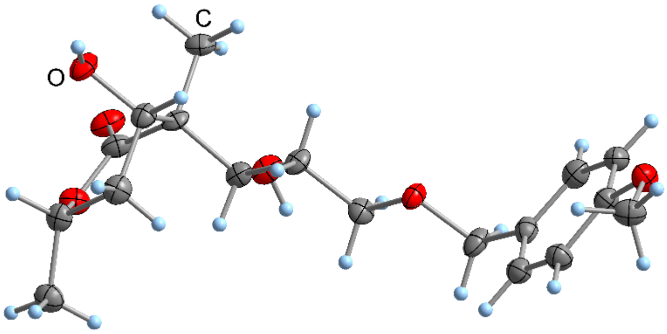

Figure S5. Molecular structure of compound **20** in the crystal. Displacement ellipsoids are drawn at the 50% probability level, H atoms as spheres of arbitrary size.

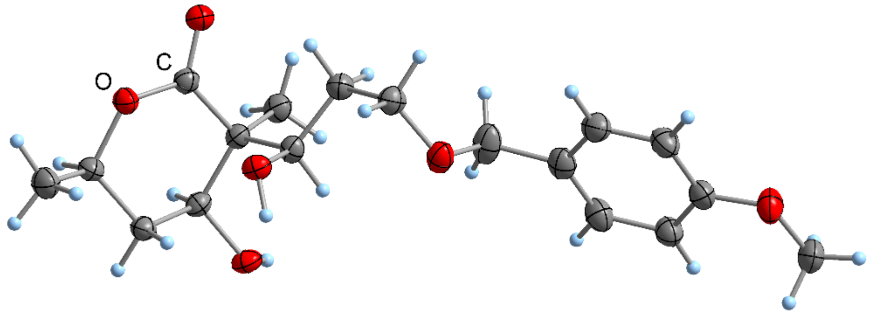

Figure S6. Molecular structure of compound **21** in the crystal. Displacement ellipsoids are drawn at the 50% probability level, H atoms as spheres of arbitrary size.

References

(S1) Bauer, T.J., Aminian, S., Spieß, O., Schinzer, D., Total synthesis of (+)-Disorazole Z1. *Chem. Eur. J.* **2025**, 31, e202501452.

(S2) Morozova, V., Skotnitzki, J., Moriya, K., Karaghiosoff, Knochel, P., Preparation of optically enriched secondary alkyllithium and alkylcopper reagents – Synthesis of (-)-Lardolure and Siphonarienal. *Angew. Chem. Int. Ed.* **2018**, 57, 5516-5519.

(S3) LeClair, C.A., Boxer, M.B., Thomas, C.J., Maloney, D.J., Total synthesis of LL-Z1640-2 utilizing a late stage intramolecular Nozaki-Hiyama-Kishi reaction. *Tetrahedron Letters* **2010**, 51, 6852-6855.

(S4) Sheldrick, G.M., SHELXT-integrated space-group and crystal-structure determination. *Acta Crystallogr.* **2015**, *A71*, 3–8.

(S5) Sheldrick, G.M., Crystal structure refinement with SHELXL. *Acta Crystallogr.* **2015**, *C71*, 3–8.

(S6) Dolomanov, O.V., Bourhis, L.J., Gildea, R.J., Howard, J.A.K., Puschmann, H., OLEX2: Complete structure solution, refinement and analysis program. *J. Appl. Crystallogr.* **2009**, *42*, 339–341.

(S7) Bruker AXS **2001**, *Apex4 and SADABS*, Bruker AXS Inc., Madison, Wisconsin, USA.

(S8) Stoe & Cie **2002**, *X-Area, X-Red and LANA*, Stoe & Cie GmbH, Darmstadt, Germany.

(S9) Parsons, S., Flack, H.D., Wagner, T., Use of intensity quotients and differences in absolute structure refinement. *Acta Cryst.* **2013**, *B69*, 249–259.

(S10) Spek, A.L., Structure validation in chemical chrystallography. *Acta Cryst.* **2009**, *D65*, 148–155.
